# Application of Artificial Neural Networks and Factorial Design Analysis for Predicting the Interaction of Influencing Process Parameters in CO_2_ Mineralization of Magnesium-Rich Mining Materials

**DOI:** 10.1021/acsomega.5c06358

**Published:** 2025-10-09

**Authors:** Iris Samputu, Hamid Radfarnia, Kourosh Zanganeh

**Affiliations:** Natural Resources Canada, 6314 CanmetENERGY, 1 Hannel Drive, Ottawa On K1a 1m1, Canada

## Abstract

Ex situ aqueous-based CO_2_ mineralization of magnesium-bearing mine wastes presents a promising pathway for carbon sequestration and resource recovery, relying on both direct and indirect carbonation approaches. However, their efficiency depends on optimizing key process parameters, such as solid/liquid particle size, pretreatment, temperature, pressure, pH, and their interactions. This study provides a comprehensive analysis of CO_2_ mineralization in magnesium-based mine wastes, utilizing an extensive data library from existing literature. Both direct and indirect mineralization approaches, including extraction and carbonation, were assessed to understand key process parameter interactions. Utilizing artificial neural networks (ANN) and a 3^k^ full factorial design coupled with Analysis of Variance (ANOVA), the study investigated nonlinear relationships and the statistical significance of influencing factors for these processes. Key findings indicated that for the extraction process, optimization is driven by feedstock material pretreatment, extraction agent, temperature, and particle size. For direct CO_2_ carbonation, prioritization of pretreatment, CO_2_ concentration, and particle size reduction are most important, with a secondary focus on solution chemistry. However, for the indirect carbonation approach, the dominance of carbonation assisting agents and solution pH highlights the importance of solution chemistry in aqueous carbonation rather than the physical properties of the feedstocks. These insights provide a robust framework for understanding the complex relationships between different process variables that play a pronounced role in the CO_2_ mineralization of magnesium-rich mining materials. Understanding influential factors enables better design and optimization of processes for enhanced efficiency and sustainability.

## Graphical abstract

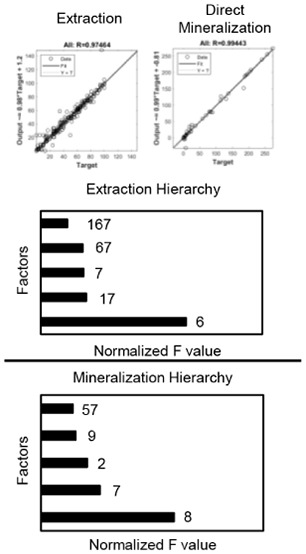

## 1 Introduction

Increasing greenhouse gas (GHG) emissions from human activities has motivated researchers to explore new pathways for mitigating their emission, specifically the one associated with the carbon dioxide (CO_2_) as the primary GHG. Carbon Capture and Storage/Sequestration (CCS) has been introduced as a viable technology option to manage the emissions of CO_2_ from the use of fossil fuels in industrial plants.^1^ Carbon capture involves the reduction in the net CO_2_ emission intensity through the implementation of active capture technologies, whereas carbon storage/sequestration involves trapping the captured CO_2_ in the suitable substrates, such as reservoirs, in the form of structural, residual, solubility, and mineral tarps.^2^ Physical simple storage of the captured CO_2_ has some associated risks including groundwater pollution, potential CO_2_ leakage, and the creation of ecological imbalance—this is in addition to the limited suitable site availabilities for underground CO_2_ sequestration.^3,4^ Among these trapping mechanisms, carbon mineralization is preferred because it is a more secure means of immobilizing the captured CO_2_ by reacting it with Mg and Ca-bearing materials to form their stable solid carbonates, preventing its later release into the atmosphere.^5–8^

Carbon mineralization is generally categorized into in situ and ex situ mineralization. In situ CO_2_ mineralization occurs when a CO_2_-rich solution (gas or liquid) is injected into a geological formation to facilitate the formation of stable solid carbonates. It also typically occurs over a long duration of time.^9–14^

In ex situ or above-ground carbonation, mineral carbonates are formed by reacting the CO_2_ with a suitable alkaline-based mineral under controlled process conditions in order to promote the carbonation rate of reactions. Hence, this process is typically faster than in situ carbonation.^3,15,16^ Example of suitable materials for mineralization includes serpentine-rich mine wastes or tailings (Mg-bearing), natural serpentine rocks rich in Mg, as well as steel slags and fly ash rich in Ca.^3,17–20^ Ex situ carbonation is then subcategorized into direct and indirect carbonation methods, where direct carbonation occurs in a one-step carbonate formation and indirect carbonation occurs in two steps: (i) metal extraction, and (ii) their conversion to carbonate products,^3,7,15,18^ which are described in detail in subsequent sections.

Mining activities produce a large quantity of waste materials rich in CO_2_-reactive alkaline minerals, which are known as a secure source for sinking the CO_2_.^21,22^ This paper therefore briefly reviews the research works on ex situ CO_2_ mineralization of serpentine-rich minerals (waste materials and natural rocks) with special attention to the parameters affecting both the extraction and carbonation stages in both the direct and indirect pathways and the potential interactions between these parameters during the carbonation process. Most of the available experimental studies involve the use of a one-factor-at-a-time experimental approach from a single cation source. This approach is generally very time-consuming and neglects the influence of interaction between participating factors, consequently leading to a poor process optimization. The application of statistical design of experiments such as three-level factorial design, Analysis of Variance (ANOVA), and neural network optimization was researched as an alternative methodology as it allows the study of the impact of multiple factors simultaneously.^23–29^

Therefore, an ANN model was developed to determine the relationship between the process parameters, extraction efficiencies, and CO_2_ mineralization capacities. Among various machine learning approaches, artificial neural networks (ANNs) outperform other models in CCUS applications due to their ability to capture complex, nonlinear relationships. ANNs excel at modeling high-dimensional data and handling noisy or incomplete data sets, which are common in mineralization studies. Their flexible architecture supports diverse data types and scales, enabling deeper insights into kinetics and thermodynamics. In contrast, traditional models like linear regression, decision trees, or SVMs often struggle with the nonlinearities and intricate interdependencies, leading to less accurate predictions. For example, Fathalian et al.^30^ made a comparison between ANN and support vector machine (SVM), gradient boosting, and random forest for CO_2_ capture prediction performance and demonstrated its superior performance. Hoonyoung Jeong et al.^31^ also compared SVM and ANN machine learning algorithms for predicting the cumulative injection of a carbon reservoir storage project and found the nonlinear functions used in ANN models to be superior in performance. Hence, it can be stated that ANN’s adaptability and predictive power make it a particularly effective tool for modeling and optimizing CO_2_ mineralization strategies. To the best of our knowledge, no prior study has explored this specific approach. Similar to this study, Song et al.^32^ developed ANN-GCS models for CO_2_ carbonation of saline aquifers achieving an *R* ^2^ of 0.9847 and an RMSE of 0.0082. The observed errors are comparable to those of the performance achieved by our model. More importantly, the approach adopted in this study focuses on mineral-specific reactions rather than relying on generalized geological storage methodologies. While a few factorial design studies exist in the context of CO_2_ mineralization,^33–36^ they primarily emphasize experimental work within constrained boundary conditions. In contrast, this study is not limited by such constraints, thereby enabling a comprehensive exploration of the entire parameter space. The trained model was then applied to the full three-level factorial design (3^k^) to predict the extraction efficiencies and CO_2_ mineralization capacities for each k-predictor variable (factor) and their interaction effects. N-way ANOVA analysis was thereafter used to study the significance of the different combinations of factors.

A similar approach was applied by Ghosal et al.^37^ for analyzing the impact of process variable changes on a water distribution network. Others have similarly used ANN and factorial designs for process optimization.^38–40^ This work provides critical insight into the prediction of the effect of different combinations of process factors, which is typically not feasible to investigate experimentally. Because the extensive experimental data library assembled for the processing of different serpentine-rich sources included the materials from different geographical locations and under a wide range of treatment conditions, the generated data are likely reliable and less prone to bias. Furthermore, this paper briefly discusses the other process considerations affecting the system performance, including regeneration pathways of extraction agents used, as well as brief economic and environmental assessments.

### 1.1 Direct Carbonation

There are two main types of direct carbonation: gas–solid (dry) carbonation and aqueous mineral carbonation. For gas–solid carbonation, a CO_2_ rich gas source is reacted directly with the alkaline-based solid to form alkaline carbonate. Because gas–solid carbonation reactions are generally slow, modifications on process conditions, such as decrease in the particle size of the solid feedstock as well as increase in process temperature and pressure, are the approaches applied to accelerate the carbonation.^3,7,41^ The most successful gas–solid carbonation studies with high mineralization extents to date have been achieved at typically high temperatures (typically above 200 °C) and high pressures (typically above 20 bar).^7,42–47^ An example for this approach is the CO_2_ mineralization study that was conducted using a pressurized fluidized bed reactor at 20–40 bar and 450–550 °C. It was found that more than 50% carbonation rates were achieved in 10 min for 170–340 μm particles.^44^ However, the gas–solid carbonation approach is outside the scope of this work.

The other direct carbonation method is the direct aqueous carbonation route. This route involves a one-step metal ion extraction and subsequent carbonation in an aqueous phase, which has been shown to increase the reaction kinetic rate compared to the gas–solid approach. This is typically a three-step simultaneous reaction process involving the dissolution and dissociation of CO_2_ in water, the extraction of metal ions specifically calcium or magnesium ions, and finally, the precipitation of calcium and magnesium elements in their carbonate forms. Usually, additives such as extraction agents are added to the aqueous system to enhance the reaction efficiency.^7,48^ Other methods of enhancing the reaction efficiency include pretreatment of the solids, decreasing the particle size, optimizing the solid/liquid ratio, and increasing the reaction time, reaction temperature, and pressure. The quality of solid feedstocks, i.e., having a higher Mg amount, is also a determining factor for enhancing the mineralization process performance.^3,7,41,49^

### 1.2 Indirect Carbonation

Indirect carbonation occurs mainly in two steps: alkaline metal ion extraction (Mg and/or Ca) followed by an individual carbonation of the leachate. Indirect carbonation is known as an aqueous-phase reaction process. A pH swing method is typically employed in this approach, where a lower pH is used for extraction and a higher pH is used for carbonation. The extraction stage is generally a reaction rate-limiting process that needs to be accelerated with the use of suitable chemical agents, such as acids. The second step is highly favored under alkaline conditions to form and precipitate the metal carbonates. This allows a high efficiency to be achieved under moderate temperature and pressure conditions.^7,50^

For solids rich in Mg, during the extraction stage, magnesium dissolves through polarization, where the Mg–O and Mg–OH bonds on the reaction surface are weakened and the Mg is carried away by the aqueous medium resulting in the subsequent ionization of non-leached layers.^3,7,41^ The factors that affect the extraction step include the particle size, Mg content of the solid feedstocks used, the solid/liquid ratio, residence time for the extraction, the extraction agent used, and the reaction temperature. Whereas for the carbonation step, other than the determining factors for the extraction step, the alkalinity of solution, CO_2_ pressure, carbonation temperature, and reaction time also affect the carbonation extent and therefore the amount of CO_2_ sequestered.^3,7,41^

### 1.3 Serpentine-Rich Mine Wastes

The serpentine-rich wastes for mineralization purposes are typically sourced from metal mines such as nickel mines, which generally contain a high Mg amount.^49,51^ Other sources for mineralization studies are natural serpentine ores. Serpentines are mainly categorized with three main crystalline phases of chrysotile, lizardite, and antigorite, which will be discussed in more detail later. Typically, the cost of dealing with this mine waste is also estimated to be about 1.5–3% of the total mine operation costs; therefore cheaper and more economical methods of dealing with this mine waste have been sought, for example, through CO_2_ mineralization processes.^18,51^ The majority of the serpentine mine wastes reported in the literature and used for CO_2_ carbonation have a MgO content that falls within the 30–50% range. The serpentine minerals are highly stable in structure and their pretreatment is generally needed to achieve high degrees of carbonation. A summary of the composition of different serpentine-rich wastes is shown in Table 1.

**Table 1:** Properties of Some Serpentine-Rich Feedstocks Sourced from Various Locations

Geographic location of waste	Mineral phase	Oxide composition	Reference
Quebec, Canada	78% serpentine, 12.6% brucite, 4.8% fortestite, 3.6% magnetite	43.07% MgO, 33.74% SiO_2_, 7.81% Fe_2_O_3_	^17^
Hitura, Finland	83% serpentine, 14% magnetite Fe_3_O_4_	38.1% MgO, 32.60% SiO_2_, 13.4% FeO	^52^
Goias state, Brazil	92% serpentine, 6.3% brucite, 1.09% magnesite, 1.96% magnetite	43.33% MgO, 40.64% SiO_2_, 12.61% Fe_2_O_3_, 1.17% Al_2_O_3_, 1.02% Cr_2_O_3_	^53^
Washington state, USA	84% serpentine, rest unknown	45.8% MgO, 0.8% CaO, 40.7% SiO_2_, 1.1% Al_2_O_3_, 4.6% Fe	^54^
Ballantrae, Scotland	92.3% lizardite, 7.5% chrysotile	38.56% MgO, 37.70% SiO_2_, 1.40% Al_2_O_3_, 7.48% Fe_2_O_3_	^55^
Cornwall, UK	98% serpentine, 2% hematite, trace of calcite	40% MgO, rest undisclosed	^56^

## 2 Materials and Methods

The objective of this study is to develop predictive models for CO_2_ mineralization using serpentine wastes and to employ these models within a factorial design and ANOVA framework to quantify the interactions among the key parameters. This approach enabled the generation of actionable insights for process design and operation through parameter sensitivity analysis. The general methodology used in this study commenced with the creation of a data library from an extensive review of the literature. An ANN model was then developed and trained using this library and validated. Once a satisfactory performance of the model was achieved, the model was applied to a full factorial design for predicting extraction efficiencies using input data of 7 parameters and to predict CO_2_ carbonation performance with input data of 9 parameters for direct and indirect mineralization processes, respectively. Combined parametric studies were then established from a statistical analysis using both a weighted average and an ANOVA method. ANOVA assumptions were tested using multiple methods: normality was assessed through visual inspection of quantile–quantile (Q-Q) plots, while homoscedasticity was evaluated using Levene’s test and analysis of studentized residuals. The resulting trends were reported. The overall steps for the methodology used are presented in Figure 1.

**Figure 1:**
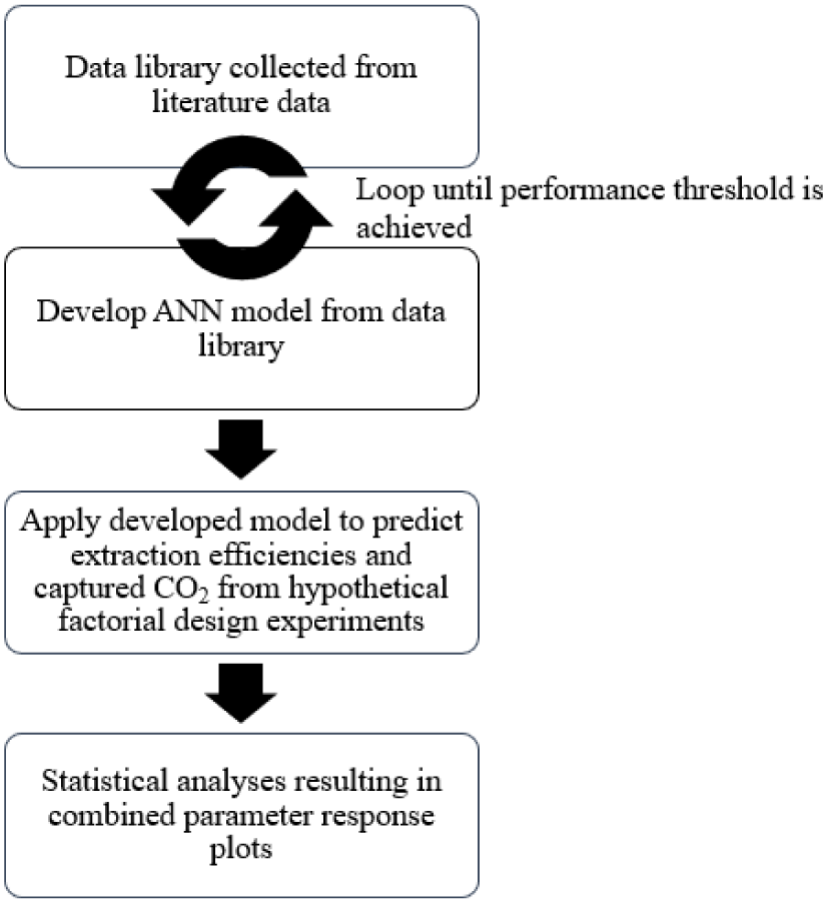
Design methodology for analysis in this work.

A similar methodology of analysis has been previously proposed for other chemical processes in the literature.^37–40,57–60^ For example, Ghosal et al.^37^ applied both a 3^k^ factorial design and ANN analysis for studying the influencing parameters in the multivariate modeling of a water distribution network.

### 2.1 Literature Data Collection

Recent literature on ex situ CO_2_ mineralization increasingly explores various feedstocks, including steel slags, tailings, and fly ashes, with a particular emphasis on mine wastes, as illustrated in Figure 2. This figure also highlights the rising number of studies specifically utilizing mine wastes for mineralization.

**Figure 2:**
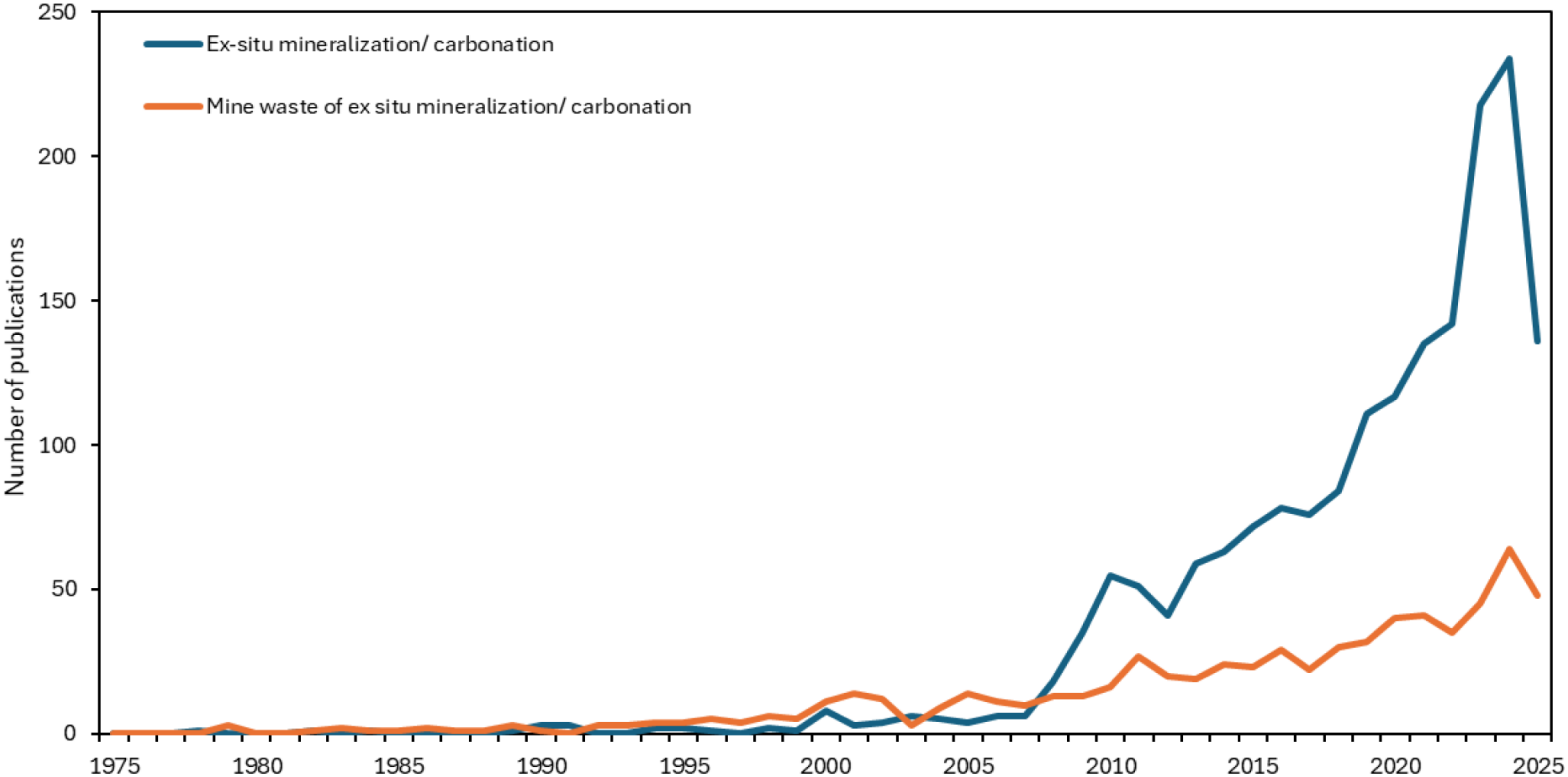
Trends of publications on ex situ CO_2_ mineralization studies, as of June 2025 (extracted from the Scopus database).

A comprehensive library for this work was compiled using Scopus, providing access to numerous peer-reviewed publications. The primary focus of this work was on publications related to Mg-bearing mine wastes and their use as feedstocks in ex situ CO_2_ mineralization processes. This includes experimental studies, simulations, kinetic modeling, and literature reviews. A bibliometric map was created using VOSviewer software^61^ to visualize the similarities of major relevant keywords from journal articles (Figure 3). This visualization was based on the keyword search used to generate Figure 2. This map groups relevant keywords and their co-occurrence in the literature, providing useful insights into the publication and citation trends for the studied subject. Similar research topics or frequently appearing keywords are grouped into the same-colored clusters. The sizes of the circles represent the number of keyword occurrences. Co-citations between articles are represented by lines connecting the circles. The thickness of the lines corresponds to the number of cocitations between keywords within the same cluster. There are four main clusters. The first cluster in red highlights words associated with the relationship between different metal mining in mineralization areas and their environmental impacts. The next cluster in green includes keywords associated with mineralization research, specifically from mine wastes. The other smaller cluster in yellow highlights the relationship between research on geochemistry and the mineralogy of materials in this area. Finally, the blue cluster centers on the environmental chemistry and wastewater treatments associated with mine wastes. These last clusters represent overlapping domains of a complex geochemical mineralization system: the yellow cluster focuses on resource characterization and economic evaluation of mineral deposits, while the blue cluster addresses environmental chemistry and remediation processes that must be managed throughout the entire resource lifecycle. All four clusters operate as interconnected components of the CO_2_ mineralization of mine waste system.

**Figure 3:**
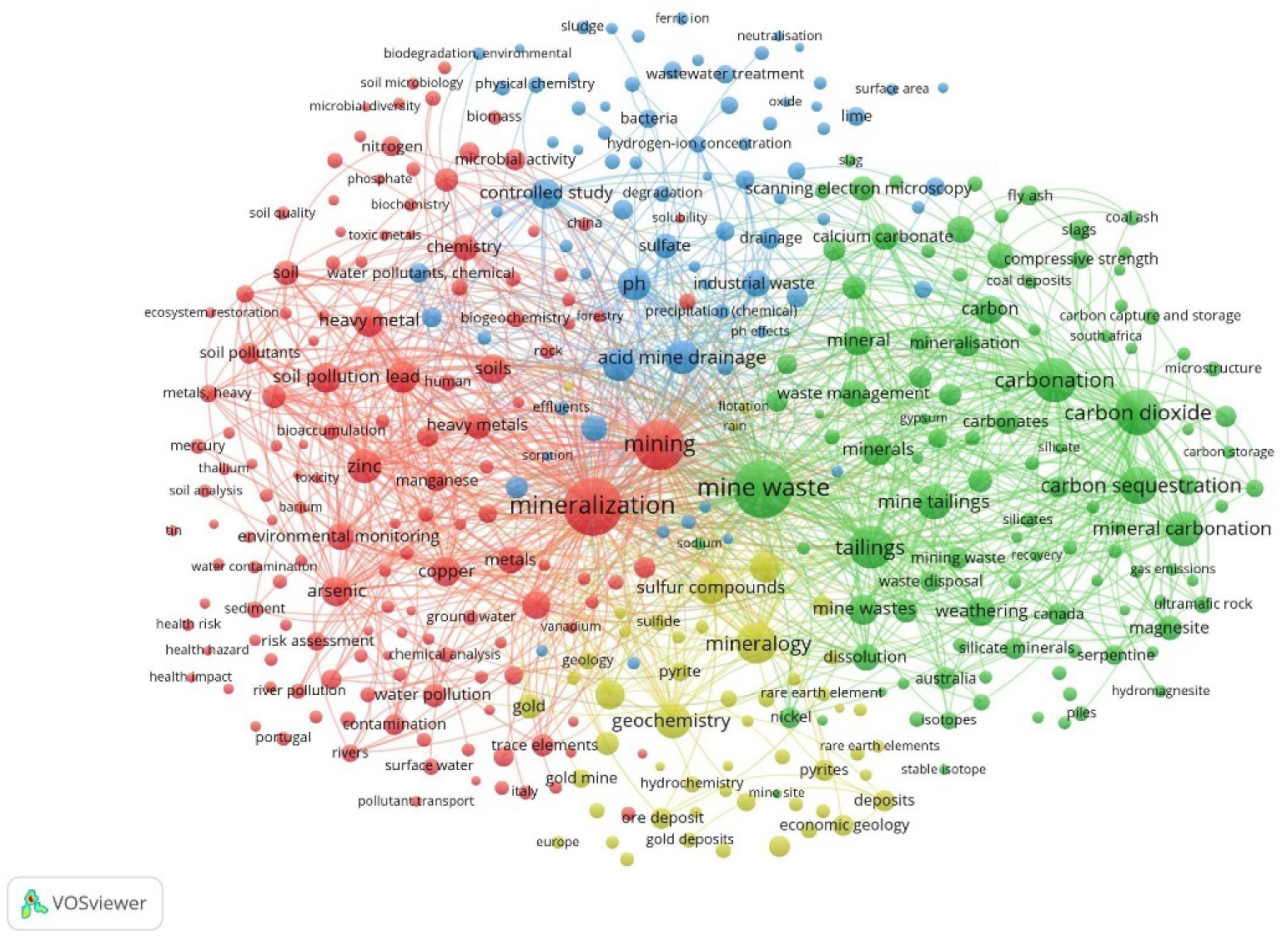
Bibliometric map of keywords created with VOSviewer software, based on the articles including experimental and modeling works for ex situ direct and indirect mineralization of various mining-based feedstocks from 1968 to 2025.

The initial data search identified over 400 literature articles that were used to generate the bibliometric map. These articles were then manually screened and narrowed down to a selection of 36 primary research articles for detailed analysis (Table S1). The inclusion criteria focused on studies employing serpentine-type mine wastes and presenting experimental results relevant to our model development, specifically those investigating direct and indirect aqueous carbonation processes conducted at temperatures below 200 °C. Of the collected works, 15 focused on direct carbonation studies, 6 covered indirect carbonation topics (extraction and mineralization), and 30 focused solely on the extraction process. Additionally, 11% of these studies were published before 2010, 32% between 2010 and 2015, 18% between 2015 and 2020, and 39% after 2020. All 36 papers were treated equally in this study. While equal weighting is a simplification, it avoids subjective bias and remains appropriate given the absence of standardized quality metrics in CO_2_ mineralization literature. This approach also preserves the statistical robustness through ensemble effects.

### 2.2 Artificial Neural Network Development

The ANN models have been successfully used as experimental predictive tools for optimization in the design of experiments across a wide range of applications.^23–29^ Such models have generally been applied in studies where it is hard to determine the governing mathematical relationships between the parameters.^62^ It can be used to identify both linear and nonlinear relationships between the studied parameters. Details of the ANN model developed in this work are presented in Section S1. The resulting ANN model was trained by using different training algorithms. The performance of these algorithms was compared and assessed using the mean squared error (MSE) and correlation coefficient *R*-values. The training algorithms used include Levenberg–Marquardt, BFGS quasi-Newton, scaled conjugate gradient, Fletcher–Powell conjugate gradient, variable learning rate backpropagation, Polak–Ribiere conjugate gradient, and one-step secant algorithms. Among these, the Levenberg–Marquardt algorithm demonstrated the best performance and is used in this work. Additionally, 10, 20, 50, and 100 layers were used to enhance model performance. The data points were split into 80%, 10%, and 10% for training, validation, and testing purposes, respectively, for extraction and direct carbonation. For indirect carbonation, 50%, 25%, and 25% split was used due to the lack of sufficient data. Overall, three methods of validation were used; internal validation during training using the validation data set to monitor overfitting, external validation using the independent test data set never seen during training, and finally validation through the ANOVA method also featured statistical significance testing adding an extra layer of validation. It is worth noting that there were 208 experimental data points for extraction, whereas the carbonation experiments had only 68 data points. This difference in the data quantity affected the result quality. Once a trained model with a high *R*-value and a low MSE was obtained, it was used to predict extraction efficiencies and sequestration performances for hypothetical full factorial design experiments. To make sure that the predicted value was in the range of 0 to 100 for extraction and above 0 for carbonation while preserving the established relationship by the neural network, a min and max normalization method was used as is standard in machine learning and data science research. Input perturbation sensitivity was applied to the ANN model, alongside a bootstrap confidence interval (*n* = 500). While the primary trained model demonstrated strong performance (*R*_test >0.9), the bootstrap results revealed substantial prediction uncertainty across the factorial design space, with a coefficient of variation averaging 76.7 ± 23.8%. This reflects the inherent variability in literature-derived data sets, which encompass diverse experimental sources, methodologies, and conditions. The findings underscore the importance of interpreting individual ANN predictions as estimates with associated uncertainty bounds. Importantly, this uncertainty analysis supports our methodological choice to use ANN outputs as inputs for ANOVA-based factorial design analysis rather than relying on individual predictions. The ANOVA framework effectively captures factor significance and interaction effects while remaining robust to the prediction uncertainties highlighted by the bootstrap analysis.^58,59^

### 2.3 3^k^ Factorial Experiment Design

In order to understand individual and combined relations between affecting parameters, a 3^k^ factorial experiment design was used for the design and analysis of the hypothetical experiments in conjunction with the ANN model. This technique has been previously used in other parametric experimental studies for mineralization as well as other ANN modeled studies.^33,37–40,63,64^ The 3^k^ factorial design is typically employed when two levels of variation (maximum and minimum probabilities) of nominated factors are not sufficient to model the factor’s impact.

The 3^k^ factorial design includes three levels: low, medium, and high for each studied factor. For the extraction and carbonation studies, the levels were defined based on their effect on the process. A low-level factor was found to have the least effect on extraction or carbonation, while a high-level factor had the most effect. These levels are represented by an additional beta coefficient variable, valued at 0, 1, and 2 for low, medium, and high levels, respectively.

For extraction, the nine parameters considered were: MgO content in feedstock, type of extraction agent used, operating temperature, reaction time, particle size, feedstock pretreatment (i.e., heat/microwave methods), and solid/liquid ratio. For direct carbonation, the affecting parameters were: MgO content in feedstock, CO_2_ gas pressure, CO_2_ concentration, solid/liquid ratio, use of a carbonation assisting agent, solution pH, reaction temperature, feedstock pretreatment (i.e., thermal or microwave methods), and particle size. For indirect carbonation, the influencing parameters were: extracted Mg concentration (from the extraction step), solution pH, reaction temperature, CO_2_ gas pressure, and type of carbonation assisting agent.

With these influencing parameters, the hypothetical experimental design resulted in 3^7^ (2187) different experiments for extraction, as well as 3^9^ (19,863) experiments for direct carbonation and 3^5^ (243) experiments for indirect carbonation. The low, medium, and high levels used to design the experiments were specified based on the collected experimental data library. Two methodologies were employed to define low-to-high levels in the factorial analysis. The first approach incorporated the entire data set (100%), thereby capturing the full spectrum of values reported in the literature. The second approach limited the analysis to the central 80% of the data, by excluding values below the 10th percentile and above the 90th percentile, effectively reducing the influence of outliers. The use of the 10th and 90th percentile thresholds aligns with Grubbs’ Test and other percentile-based outlier techniques.^65–67^ This approach was adopted based on statistical theory, which indicates that extreme outliers can disproportionately affect the training of neural networks and the outcomes of factorial analysis, potentially resulting in models that underperform under typical operating conditions. Therefore, the latter method was applied to attenuate the influence of such outliers, given that some experimental data points fell outside the expected range. The low, medium, and high levels for factorial analysis are shown in Tables 2–4 for extraction and carbonation (direct and indirect), respectively. For example, no heating or microwave pretreatment was indexed as 0, thermal pretreatment was indexed as 1 and microwave pretreatment was indexed as 2. For extraction agents, inorganic acids were indexed as 2, organic acids, weak acidic salts of ammonium, and other reagents (including chelating agents and carbonic acids) were indexed as 1, and no additive/water was indexed as 0. For carbonation assisting agents (indirect), strong bases were indexed as 2, a combination of a carbon source, a buffer base, and/or chelating agent (such as NaHCO_3_ with NaCl), tiron, and triethylamine) as 1, and no additive/water as 0. This classification was based on the expected performance on the extraction efficiency and the amount of CO_2_ captured. For carbonation assisting agents (direct), a high index (2) was assigned to the carbon source with buffers/additives (such as NaHCO_3_ with NaCl or EDTA). A medium index (1) was assigned to the use of the carbon source alone (NaHCO_3_), and a low index (0) was assigned to the use of just water. The models developed in this study are based on literature data that primarily involve pure CO_2_ or CO_2_/N_2_ mixtures, which may not fully capture the complexity of industrial flue gas. Trace impurities such as NO_*x*_ and SO_*x*_ could affect carbonation through competitive reactions. Therefore, the predictions should be viewed as baseline estimates under controlled conditions, with further validation needed for real-world applications.

**Table 2:** 3^k^ Factorial Design Parameters for the 2187 Extraction Experiments

			Values			
	Low (0)		Medium (1)		High (2)	
Influencing factors	100% data	80% data	100% data	80% data	100% data	80% data
Factor 1-MgO feed content (%)	0.85	27.1	29.405	36.45	57.96	45.8
Factor 2-Particle size (μm)	1000	250	505	152.1	10	54.2
Factor 3-Solid/liquid ratio (g/L)	200	50	100.5	27.5	1	5
Factor 4-Operating temperature (°C)	20	22	97.5	61	175	100
Factor 5-Time duration (h)	0.3	0.7	25.15	2.35	50	4
Factor 6-Pretreatment	No heat		Thermal		Microwave	
Factor 7- Extraction agent	No extraction agent		organic acids, and weak acidic salts of ammonium		Strong inorganic acids	

**Table 3:** 3^k^ Factorial Design Parameters for the 19,863 Carbonation Experiments—Direct

	Values					
	Low (0)		Medium (1)		High (2)	
Influencing factors	100% data	80% data	100% data	80% data	100% data	80% data
Factor 1-MgO feed content (%)	9.47	9.47	30.535	28.095	51.6	46.72
Factor 2-Particle size (μm)	850	150	427.5	87.992	5	25.984
Factor 3-Solid/liquid ratio (g/L)	502	150	253.513	80	5.025	10
Factor 4-Reaction temperature (°C)	20	22	120.5	103.5	220	185
Factor 5-Reaction pH	6	6.47	7.805	7.433	9.61	8.4
Factor 6-CO_2_ pressure (MPa)	0.0096	0.01	10.0048	7.505	20	15
Factor 7-CO_2_ concentration (%)	9.41	10	54.705	55	100	100
Factor 8-Pretreatment	No heat		Thermal		Microwave	
Factor 9- Carbonation assisting agent	None used		NaHCO_3_		Carbon source/buffer added (NaHCO_3_, with NaCl or EDTA)	

**Table 4:** 3^k^ Factorial Design Parameters for the 243 Carbonation Experiments—Indirect^a^

	Values					
	Low (0)		Medium (1)		**High (2)**	
Influencing factors	100% data	80% data	100% data	80% data	100% data	80% data
Factor 1-Extracted Mg concentration (molar)^b^	0.11	0.36	0.54	0.65	0.96	0.94
Factor 2-Reaction temperature (°C)	18	28.8	86.5	62.65	155	96.5
Factor 3-Reaction pH	8	8	9.5	9.5	11	11
Factor 4-CO_2_ pressure (MPa)	0.1	0.1	7.55	6.38	15	12.66
Factor 5- Carbonation assisting agent	None used		Buffer/chelating agent and carbon source or amine agent (NaHCO_3_, with NaCl, Tiron, triethylamine, etc.)		Strong bases (e.g., NaOH)	

a Literature data available included data with 100% CO_2_ only, thereby, the CO_2_ concentration was not a parameter, due to the lack of information.

b The amount Mg in the leachate for carbonation was obtained from the extraction step.

### 2.4 ANN Application and Statistical Analysis

The previously developed ANN model was applied to predict responses, that is, extraction efficiencies and CO_2_ sequestration capacities, for all hypothetical experiments obtained from the 3^k^ factorial design methodology. Thereafter, two methods were used to analyze the effects of individual and combined factors. The first method was a weighted average method based on a modular arithmetic approach, and the second was an N-way Analysis of Variance (ANOVA).^37,68^ The results from the latter method will be the focus and are presented here due to its more thorough approach. ANOVA was conducted using the statsmodels module in Python to evaluate the statistical significance of our experimental results. The analysis using the ANOVA method was done based on the *F*-value, *p*-values, and degrees of freedom value (D.f.). The *F*-value is the variance between the groups versus the variance within the groups. The groups, in this case, are the different factors and factor combinations. The *p*-value is derived from the *F*-value. This *F*-value is used to test the statistical significance of results using a *p*-value equal to the confidence margin.^69^ In this case, the respective statistical significance of the contribution of the main and interacting parameters was inferred at a significance level of *p* ≤ 0.05 (95% confidence), where parametric contributions with *p*-values greater than the 0.05 were discarded.^69,69–71^ The calculation of these values was also based on Cochran’s theorem.^69,71^ This method has been used in the process parametric optimization of ammonia-coal cofiring in a pilot-scale fluidized bed reactor,^71^ as well as in the optimization of gasification process parameters in COVID-19 medical masks,^72^ among applications in other industries.^70,73,74^

### 2.5 Factors Affecting the Extraction Process

#### 2.5.1 Pretreatment

Ultramafic mine tailings are usually composed of serpentine minerals. The serpentine mineral is part of the layered magnesium phyllosilicate group and is generally defined by the chemical formula Mg_3_Si_2_O_5_(OH)_4_ (lizardite and chrysotile) or Mg_48_Si_34_O_85_(OH)_62_ (Antigorite).^75–77^ Natural serpentine minerals typically have a highly stable structure, as they are composed of a highly stable 1:1 TO layer configuration, where T is the tetrahedral layer and O represents the octahedral layer. The tetrahedral layer is composed of silica (Si) and three oxygen (O) atoms, which are shared with adjacent tetrahedrons. This octahedral layer also contains another oxygen atom that binds with the Magnesium ion, imparting high stability. Other impurities such as Ni, Fe and Al can also be found in place of Mg in the structure of natural serpentine.^75–77^ Regarding the mineral varieties of serpentine, antigorite and lizardite have high SiO_2_ concentrations, with antigorite having a low H_2_O concentration.^78,79^ Conversely, chrysotile has high H_2_O and MgO contents, while lizardite has a low FeO concentration in addition to high SiO_2_ content.^78,79^ This imparts the difference in their physical structures, where lizardite and chrysotile have a planar structure of different plane shapes, whereas antigorite has a corrugated structure.^75,77–79^ Due to the highly stable nature of serpentine, preactivation favors the extraction of Mg ions during the extraction process. Three main methods of preactivation are applied: mechanical, chemical, and thermal. Thermal activation is favored above the dehydroxylation temperature of serpentine, where dehydroxylation reaction occurs, releasing hydroxyls as water vapor from its structure.^78^ The improvement in the solubility and dissolution rate of serpentine attributed to thermal preactivation has already been addressed in the literature.^78,80–82^ One can expect that, due to differences in physical structure, the thermal stability of the serpentine types varies. At elevated temperatures over 550 °C, serpentine begins to undergo thermal decomposition.^83,84^ Chrysotile is stable up to 654 °C, while lizardite and antigorite, on the other hand, are stable up to about 715 °C.^79,80,85^ Besides dehydroxylation, thermal activation can also change other physical properties such as porosity, specific surface area, and degree of crystallinity, which may further improve the reactivity of serpentine-rich minerals with CO_2_ gas.^77,78,84,85^ Microwave heating has also been researched as an alternative to high temperature calcination. Although the operational costs are lower with microwave treatment, due to lower processing duration and temperatures, the cost associated with equipment and difficulty in scale-up may limit its industrial application.^75,86^ Another preactivation strategy is mechanical activation. This is typically done through milling and grinding, where these actions introduce pressure and shear forces, leading to the activation of serpentine by reducing the particle size of minerals and disordering their crystal structure.^13,75,87,88^ Moreover, mechanical activation accelerates dehydroxylation by disordering the serpentine crystal structure and delaying the recrystallization of activated serpentine, as confirmed by microscopical analyses.^75,77,78^ The degree of activation of serpentine depends on the mill type used, the grinding temperature (if not done at room temperature), and the use of grinding aids such as water. Li and Hitch^89^ compared the use of the most popularly available mills, namely, planetary, vibratory, and stirred mills, and found that the stirred mill was more effective than the others.^89^ The use of water as a milling agent, on the other hand, has been shown to have little effect on material activation.^77,78,90^ Although mechanical activation increases the surface area and extent of extraction by disordering the crystal structure, the energy consumption for the process is a serious challenge, leading to increased operational cost and CO_2_ emissions in the carbonation processes.^75,77,78,90^ A recent study by Rim et al.^48^ investigated structural changes in heat-treated Mg-bearing silicates during direct CO_2_ carbonation process using ^29^Si MAS NMR, XRPD, and ICP-OES characterizations. They found that natural serpentine was composed of a single crystalline silicate structure (dehydroxylate II and serpentine), while the heat-treated serpentine was a mixture of amorphous (dehydroxylate I, enstatite, and silica) and crystalline phases (forsterite, dehydroxylate II, and serpentine). For the extraction process, it was demonstrated that both Mg and Si in the amorphous silicate structures were more soluble than those in the confined crystalline structures. It was also noted that the extraction of Mg is limited by the formation of a Si-rich passivation layer on some mineral particles, where early dissolved silicon undergoes reprecipitation. It was recommended that in situ heat treatment and mechanical grinding can lead to the formation of more soluble layers by removing the insoluble passivation layer and distorting the highly stable crystalline phases. As a cheaper alternative to heat and mechanical activation methods, chemical treatment coupled with the earlier discussed methods was proposed to lower the total energy consumption.^48^ Liu et al.^75^ investigated the chemical activation of serpentine-rich material by adding chemicals such as fluorite powder. They found that the addition of 5 wt.% fluorite powder improved the extraction efficiency of magnesium by about 36% due to an F–Si complex formation, which disordered the TO-layered structure of the serpentine.^75^

#### 2.5.2 Magnesium Content

The magnesium content in the feed is one of the most important factors affecting extraction performance, as it determines the amount of Mg cations that will be leached out. Typically, the Mg amount in serpentine-rich feedstock is expressed in the form of MgO and generally ranges from about 30% to 50% (according to this work’s data library). The presence of other leachable metals, such as Fe and Al, can also influence the efficiency of Mg extraction.^49^ A study conducted by Wang and Dreisinger^91^ compared metal extraction and CO_2_ mineralization with three different Mg-bearing wastes: olivine, saprolite, and limonite, with 45.5, 24.2, and 13.4 wt.% of MgO, respectively. The authors found that more Mg was leached from limonite compared to saprolite (2.5% compared to 0.68%), although saprolite contained more MgO. This was explained by the presence of other leachable metal ions in saprolite that competed with Mg, namely iron (Fe) and aluminum (Al).^91^

#### 2.5.3 Particle Size

The particle size has also been studied extensively as one of the important parameters determining the extraction efficiency. Generally, a smaller particle size leads to a higher Mg extraction efficiency. Sanna et al.^56^ studied the effect of particle size on Mg extraction from a natural serpentine rock containing 98 wt.% serpentine and 2 wt.% hematite.^56^ In this study, the influence of other parameters, including temperature, extraction agent concentration, solid/liquid ratio, and pressure, was also investigated. Regarding the effect of the particle size, four different ranges were investigated: <75, 75–150, 150–300, and 500–700 μm. The operating temperature was 100 °C, with a solid/liquid ratio of 50 g/L and a chemical reagent (NH_4_HSO_4_) concentration of 1.4 M. It was found that reducing particle sizes increased the Mg extraction efficiency: approximately 90% Mg extraction efficiency with <75 μm particles versus 78% for 75–150 and 150–300 μm particles, and 61% for 500–700 μm. In another study, Arce et al.^92^ investigated the variation of Mg extraction efficiency from a serpentine-based material (∼43 wt.% MgO) with critical parameters, namely particle size, temperature, and extraction agent (HCl) concentration. The authors concluded that a decrease in particle size increased the Mg extraction efficiency; however, the particle size had less impact on the extraction of Mg than did the temperature. Other researchers have also noted that decreasing particle size increases the Mg extraction rate.^55,93–95^

#### 2.5.4 Solid/Liquid Ratio

The solid/liquid ratio is another factor that influences the magnesium extraction efficiency. This parameter affects not only capital expenditure but also operating costs because it determines the throughput volume of feed solution in the system. Sanna et al.^56^ studied the effect of the solid/liquid ratio on the extraction of Mg from natural serpentine rock and concluded that a decrease in the solid/liquid ratio from 100 to 25 g/L increased the extraction efficiency from 72% to 90% at 140 °C and using 2.8 M NH_4_HSO_4_ within 60 min of treatment.^56^ In another study, Steel et al.^96^ reported a different trend, where the Mg extraction efficiency was decreased from 85% to 60% when the solid/liquid ratio was changed from 50 to 5 g/L for a natural rock serpentine feedstock treated by 0.025 M HCl solution. On the other hand, Gao et al.^94^ reported that a decrease in the solid/liquid ratio from 40 to 10 g/L led to an increase in the Mg extraction efficiency from 13% and 16% when the extraction of calcined serpentine mineral was performed for 200 min at 70 °C with NH_4_Cl extraction agent. Therefore, one can say that the synergy between various parameters can change the level of contribution to extraction efficiency for an individual studied parameter, such as the solid/liquid ratio. Hence, the impacts of other parameters must be considered in such studies.

#### 2.5.5 Extraction Agent

The type of the extraction agent used during the extraction stage is also determined for extraction efficiency. There are generally three main categories of extraction agents used for CO_2_ mineralization studies: inorganic acids, organic acids, and ammonium salts. Other less commonly used extraction agents include chelating agents and carbonic acid. From the literature data, one can conclude that inorganic acids have better extraction performance than organic acids and ammonium salts. However, they are less easy to regenerate. Teir et al.^97^ compared the performance of HCl (4 M) with HNO_3_ (4 M) for extracting Mg ions from natural serpentine rock at 70 °C and found that HCl-treated mineral exhibited better efficiency than the one treated with HNO_3_.^99^ In another study, Teir et al.^95^ compared the use of both inorganic (HCl, HNO_3_, H_2_SO_4_) and organic (CH_3_COOH and HCOOH) acids as extraction agents for Mg extraction at 20 °C from natural serpentine rock. They found that H_2_SO_4_ was the most effective agent, followed by HCl, HNO_3_, HCOOH, and then CH_3_COOH.^95^ Nevertheless, few studies have focused on the use of H_2_SO_4_, as its regeneration is more difficult compared to that of HCl and HNO_3_, due to the excessive energy required for its high boiling point.^52,97^ Park et al.^98^ also compared the extraction efficiency of different acids, namely HCl, acetic acid, a mixture of acetic acid and sodium acetate, ascorbic acid, potassium hydrogen phthalate (KHP), ethylenediaminetetraacetic acid (EDTA), and a mixture of orthophosphoric acid, oxalic acid, and EDTA, for Mg extraction from natural serpentine rock. The highest Mg extraction efficiency was achieved by the latter, i.e., the mixture of orthophosphoric acid, oxalic acid, and EDTA. Bobicki et al.^54^ investigated the extraction performance with catechol, citrate, EDTA, oxalate, and tiron chelating reagents for serpentine-based nickel mine waste and reported that the efficiency was higher with EDTA than with citrate, followed by oxalate, tiron, and lastly catechol. Ammonium salts are another category of solvents that have been widely investigated in the literature. Ammonium chloride (NH_4_Cl), ammonium nitrate (NH_4_NO_3_), ammonium acetate (CH_3_COONH_4_) and ammonium bisulfate (NH_4_HSO_4_) are examples of these chemicals. Wang and Maroto-Valer^99^ compared the performance of various ammonium salts for the extraction of naturally sourced serpentine. They concluded that NH_4_HSO_4_ with 50% extraction efficiency was the best-performing agent when experimentally compared to ammonium chloride (∼3%), ammonium sulfate (∼3%), and sulfuric acid (∼45%). This study was conducted using 2 M concentrated ammonium salts at 70 °C for 3 h on 40.1% MgO non-pretreated serpentine with a 75–150 μm particle size and a solid–liquid ratio of 50 g/L. Another study reported that NH_4_HSO_4_ outperformed other salts including NH_4_Cl, NH_4_NO_3_, and CH_3_COONH_4_ with a Mg extraction efficiency of up to 23% compared to 15% for NH_4_NO_3_ and 12% for CH_3_COONH_4_ during the extraction of chrysotile.^100^ It is important to highlight that these results are from extraction experiments of waste slate on chrysotile, where chrysotile was the magnesium source. Moreover, this study was conducted using nonpretreated slate with less than 425 μm particle size with a 2.2% elemental Mg concentration and a 25.2% Ca concentration using 1 M ammonium salt with a 20 g/L solid/liquid ratio for 4 h at room temperature.

The extraction efficiency of the extraction collected from the literature formed the basis of the order of the extraction agents used in the model for the extraction efficiency.

#### 2.5.6 Reaction Time

The reaction time also affects the extraction efficiency, where a longer duration generally increases the extraction efficiency until it reaches a plateau.^56,101–104^ This plateau point depends on other influencing factors, including the extraction agent, particle size, reaction temperature, and solid/liquid ratio. Sanna et al.^56^ conducted a parametric extraction study with NH_4_HSO_4_ and reported that the extraction efficiency typically reached the plateau by 180 min at temperatures above 100 °C, with a particle size of under 300 μm and a solid/liquid ratio of 50 g/L, while the NH_4_HSO_4_ concentration was varied. On the other hand, at a constant NH_4_HSO_4_ concentration of 1.4 M and with varying particle sizes of 75 to 700 μm, the extraction efficiency did not reach a plateau and continuously increased during 180 min of operation. A similar observation has been noted by others, where the extraction system conditions, such as the extraction agent and concentration, particle size, and solid/liquid ratio, determined the time for reaching the extraction plateau.^102,103^

#### 2.5.7 Operating Temperature

The last influencing parameter is the operating temperature. Compared to other affecting parameters, such as mineral particle size and acid concentration, it was shown that the operating temperature has more influence on extraction efficiency.^56,92,96,102^ For example, Sanna et al.^56^ achieved about 27% Mg extraction efficiency at 50 °C versus over 75% at 100–140 °C for the extraction of natural serpentine rock that was chemically treated with 1.4 M NH_4_HSO_4_ solution. A similar trend was observed for the processing of laterite nickel waste with 2.5 M HCl solution, where Mg extraction efficiencies of 30% and 42% were obtained at 50 and 100 °C, respectively.^102^ Arce et al.^92^ also reported an increase in Mg extraction efficiency from 40% to 97% with an increase in temperature from 25 to 100 °C when serpentine mine waste was chemically treated with 2 M HCl. Such observations can be attributed to an increase in the solution energy with a rise in temperature. This resulted in a faster Mg dissolution rate, as Mg dissolution is a temperature-dependent chemical reaction with a diffusion control mechanism.^95,105,106^ The true activation energy for natural serpentine dissolution in organic acids HCl, HNO_3_, and H_2_SO_4_ was estimated to be around 140–150 kJ/mol, making it an endothermic process that is facilitated by an input of energy for dissolution to occur.^95^ Another reason for the enhanced dissolution rate at elevated temperatures could be structural modifications and changes in the material’s degree of crystallinity, promoting amorphization. For instance, lizardite-rich serpentine undergoes facilitated amorphization at 140 °C, as noted in ref. 54.

### 2.6 Factors Affecting the Carbonation Process

#### 2.6.1 Mg Content

Similar to the extraction process, a higher Mg amount in the feedstock typically results in a higher amount of CO_2_ sequestered. This relationship, however, is also affected by the presence of other metal impurities. Wang and Dreisinger^91^ compared the carbonation of olivine, saprolite, and limonite with MgO content of 45.5%, 38.3%, and 13.4%, respectively, and found that CO_2_ sequestration capacity decreased as the Mg content decreased (242, 207, and 131 g/kg of CO_2_).^91^ A similar observation was also reported by Assima et al.,^101^ where a higher carbonation efficiency for a nickel tailing with more MgO content was obtained than for material with lower MgO.^101^ For indirect carbonation, a higher amount of MgO in the feedstock generally results in a greater amount of Mg extracted in the leachate, which subsequently favors carbonation.

#### 2.6.2 Particle Size

A smaller particle size is also anticipated to result in an increased amount of CO_2_ sequestered during the direct carbonation process. Although this has not been widely studied with serpentine-based wastes, it is hypothesized that in situ extraction of Mg species increases with a decrease in solid particle size, comparable to observations noted for individual extraction studies in Section 2.5.3. A similar observation was reported by Eikeland et al.,^107^ where the extraction efficiency of naturally sourced olivine (Mg_2_SiO_4_) in a direct carbonation study was increased from <5% to approximately 100%, when the particle size was reduced from 100 to 10 μm in the presence of NaCl and NaHCO_3_ solution.^107^ Similarly, another study on the carbonation of natural serpentine rock in a gas–solid system found that reducing the particle size of the mineral increased carbonation in a fluidized bed reactor operating at 20–40 bar and 450–550 °C.^44^

#### 2.6.3 Solid/Liquid Ratio

For the direct carbonation system, the solid/liquid ratio can also potentially affect the carbonation performance. Although none of the reviewed direct carbonation works studied the effect of the solid/liquid ratio solely on the amount of CO_2_ sequestered, it is presumed that a decrease in the solid/liquid ratio would lead to an increased amount of CO_2_ sequestered. However, the degree of its impact depends on the range of solid:liquid ratios used and other influencing parameters. A similar conclusion has already been drawn for the carbonation of industrial slags, such as carbide and steel slags.^108,109^ For instance, one study reported that the amount of CO_2_ sequestered increased from 82 to approximately 105 g-CO_2_/kg-slag with a decrease in the solid/liquid ratio from 150 to 50 g/L. This was observed when the carbonation of <75 μm steel slag was conducted at 60 °C with a CO_2_ concentration of 15 vol.%.^109^

#### 2.6.4 Carbonation Assisting Agent

The carbonation reaction is promoted under alkaline conditions, generally achieved by adding alkaline chemical agents. Ammonium salts, sodium bicarbonate (NaHCO_3_), sodium carbonate (Na_2_CO_3_), and sodium hydroxide (NaOH) are examples of chemical reagents that have been widely used for this purpose in the literature.^54,91,101,104,110–113^ NaHCO_3_, a carbon carrier alkaline chemical, in particular, is one of the most popular agents used for both direct and indirect carbonations.^54,91,104,110,111^ The buffering effect of NaHCO_3_ in aqueous solutions and the reduced need for excessive injection of the CO_2_ gas stream for carbonation are the major advantages.^104,107,114,115^ Ammonium-based salts are weak-base reagents that have been addressed in the literature as carbonation assisting agents. These agents usually include NH_4_OH, and NH_4_HCO_3_. Teir et al.^95^ and Zhang et al.^116^ demonstrated the economic advantage and ease of recovery when using such weak bases. Nevertheless, few studies are available on their application in serpentine mine wastes. Ferrufino et al.^53^ applied the NH_4_OH agent for the carbonation of lizardite in a pH swing system (HCl/NH_4_OH), successfully producing high-purity hydromagnesite. In another work, Wang and Maroto-Valer^117^ studied the mineralization of a natural serpentine using NH_3_ and NH_4_HCO_3_, as carbonation agents. According to their study, approximately 7.48 tonnes of ammonium bicarbonate (NH_4_HCO_3_) and 0.04 tonnes of ammonia (NH_3_) are required to store 1 tonne of CO_2_ in 2.63 tonnes of serpentine, primarily as hydromagnesites.

#### 2.6.5 CO_2_ Concentration and Pressure

The feed gas CO_2_ concentration and operating pressure are also determining factors for the CO_2_ sequestration system. The feed gas can be either a flue gas stream typically with a CO_2_ concentration of a 5 to 20 vol.% or a pure source of CO_2_.^81,82,102,118,119^ When considering the economics of using pure CO_2_ as a feed gas source, the associated transportation and the need for upstream purification treatment costs need to be considered.^120,121^ From the literature, one can conclude that a higher concentration of CO_2_ in the feed gas results in a higher amount of the CO_2_ mineralization capacity. On the other hand, the pressure of CO_2_-containing gas stream is also important, and generally a higher pressure results in a higher amount of mineralization.^54,101,104,122^ Gadikota et al.^104^ reported an increased extent of direct carbonation of olivine with pure CO_2_ from 39.3% to 83.9% when the CO_2_ pressure was increased from 6.4 to 16.4 MPa after 3 h of carbonation at 185 °C in the presence of 1 M NaCl and 0.64 M NaHCO_3_.^104^ Galina et al.^112^ also noted an enhanced extent of the carbonation reaction from 66% to 90% with an increase in CO_2_ pressure from 0.1 to 15 MPa for the indirect carbonation of natural serpentine at a pH of 11 using NaOH.^112^ A similar observation has also been addressed for the direct carbonation of other Ca-based alkaline wastes.^123,124^ For example, Kim and Azimi^124^ noted an increase in the amount of CO_2_ sequestered from 128.2 to 145.1 g-CO_2_/kg-slag with an increase in the CO_2_ pressure from 9 to 11 MPa. The authors, however, noted that CO_2_ pressure was not the only affecting parameter, and temperature as well as particle size were also imperative.^124^

#### 2.6.6 Reaction Temperature

Reaction temperature is one of the most affecting parameters on the extent of carbonation; the higher the reaction temperature, the greater the extent of carbonation. Gadikota et al.^104^ indicated that the carbonation extent of olivine material increased from about 3% to 83% with an increase in temperature from 90 to 185 °C, with a CO_2_ pressure of 13.9 MPa and in the presence of 1 M NaCl and 0.64 M NaHCO_3_. A similar observation was reported by Werner et al.^14^ for the direct carbonation of preactivated natural serpentine material. The authors noted an increase in the amount of carbonates formed from 12.6% to 16.2% with an increase in temperature from 30 to 60 °C when experiments were conducted at a partial CO_2_ pressure of 0.1 MPa and with a solid/liquid ratio of 100 g/L.^128^ Similar studies using Ca-based alkaline wastes indicated an increase in the extent of CO_2_ mineralization specifically with increased reaction temperatures in direct carbonation experiments.^123,124^ In this context, Kim and Azimi^124^ reported that when the temperature was increased from 50 to 70 °C, the amount of CO_2_ sequestrated increased from 128.2 to 172.1 g-CO_2_/kg-slag for experiment with 105 μm sample, at 9 MPa CO_2_ pressure and a solid/liquid ratio of 2500 g/L.

#### 2.6.7 Reaction pH

The solution pH needs to be in the appropriate range to allow the carbonation reaction to proceed. For indirect carbonation, the optimal pH to ensure the carbonation reactions take place is about 9–10.^98,104,114,125,107^ For direct carbonation, a pH of around 6–7 is typically preferred to ensure both extraction and carbonation are simultaneously advanced.^91,104^ Therefore, a pH swing is typically employed with indirect carbonation operations to facilitate the optimized low-pH extraction of Mg and the high-pH formation of carbonates.^7,54,100,104,131,132^ ^,^

## 3 Results and Discussion

### 3.1 Extraction Analysis

#### 3.1.1 Literature Data ANN Modeling

The ANN methodology was used for the model creation and training as explained earlier in Section 2. The model’s performance was assessed through the *R* and MSE values. Also, the collected experimental data were used in the order of 80% for training, 10% for validation and 10% for testing. The resulting model used for extraction had an average MSE and *R* values of 39.7854 and 0.97464, respectively, for combined training, validation, and testing. The resulting percentage root-mean-squared error (RMSE) was about 6.308%, which indicates an adequate model fit with the experimental data collected. The model’s performance is shown in Figure 4.

**Figure 4:**
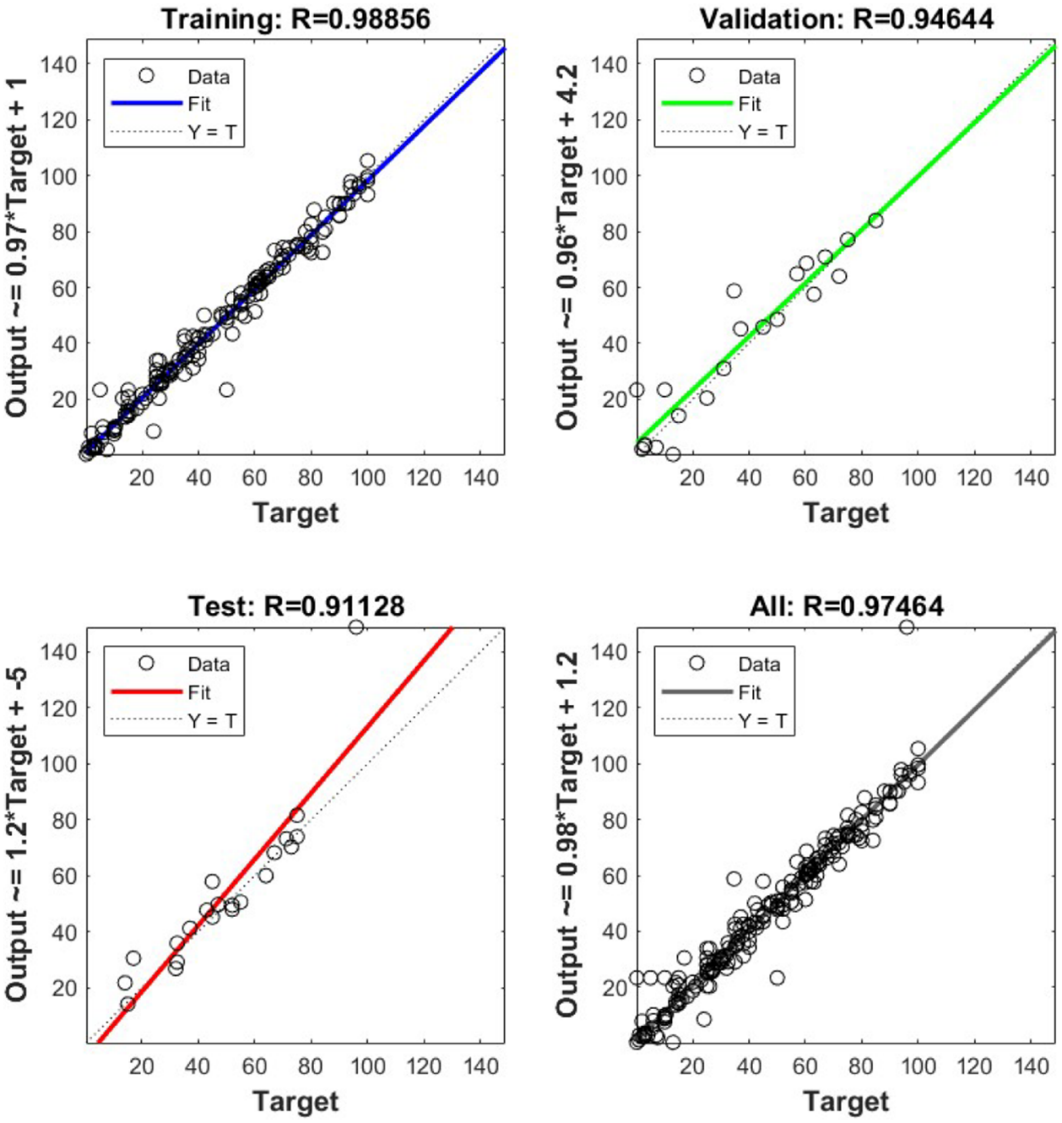
Model correlation performance for the extraction efficiency results.

The input perturbation sensitivity analysis was also performed on the chosen model to assess the overall effect of a change in each individual parameter in the predicted efficiency results by the ANN-assisted developed model. The input perturbation algorithm includes the introduction of a change in the input and then assessing the effect of this change on the output through the calculation of RMSE or MSE.^129^ The perturbation introduced to the model ranged between 0.1% and 50%.

As can be seen in Figure 5, perturbation in Factor 1 had the most influence on the error in extraction efficiency; therefore, the model is very sensitive to the MgO content in the feedstock. This is followed by Factors 3 (solid/liquid ratio) and 4 (temperature). The factors with the least perturbation effect on the extraction efficiency are Factors 5 and 6 (time duration and pretreatment). This can be justified as the time-related changes in extraction efficiencies occur over a longer period compared to the changes caused by variations in the MgO amount. Moreover, there were also a few numbers of studies available that applied the pretreatment methods (heat and microwave). It should be noted that the detailed individual and combined parameter effects for the developed model were conducted through a full factorial and statistical ANOVA analysis, as presented in the next section, using set low, medium, and high levels based on collected literature data.

**Figure 5:**
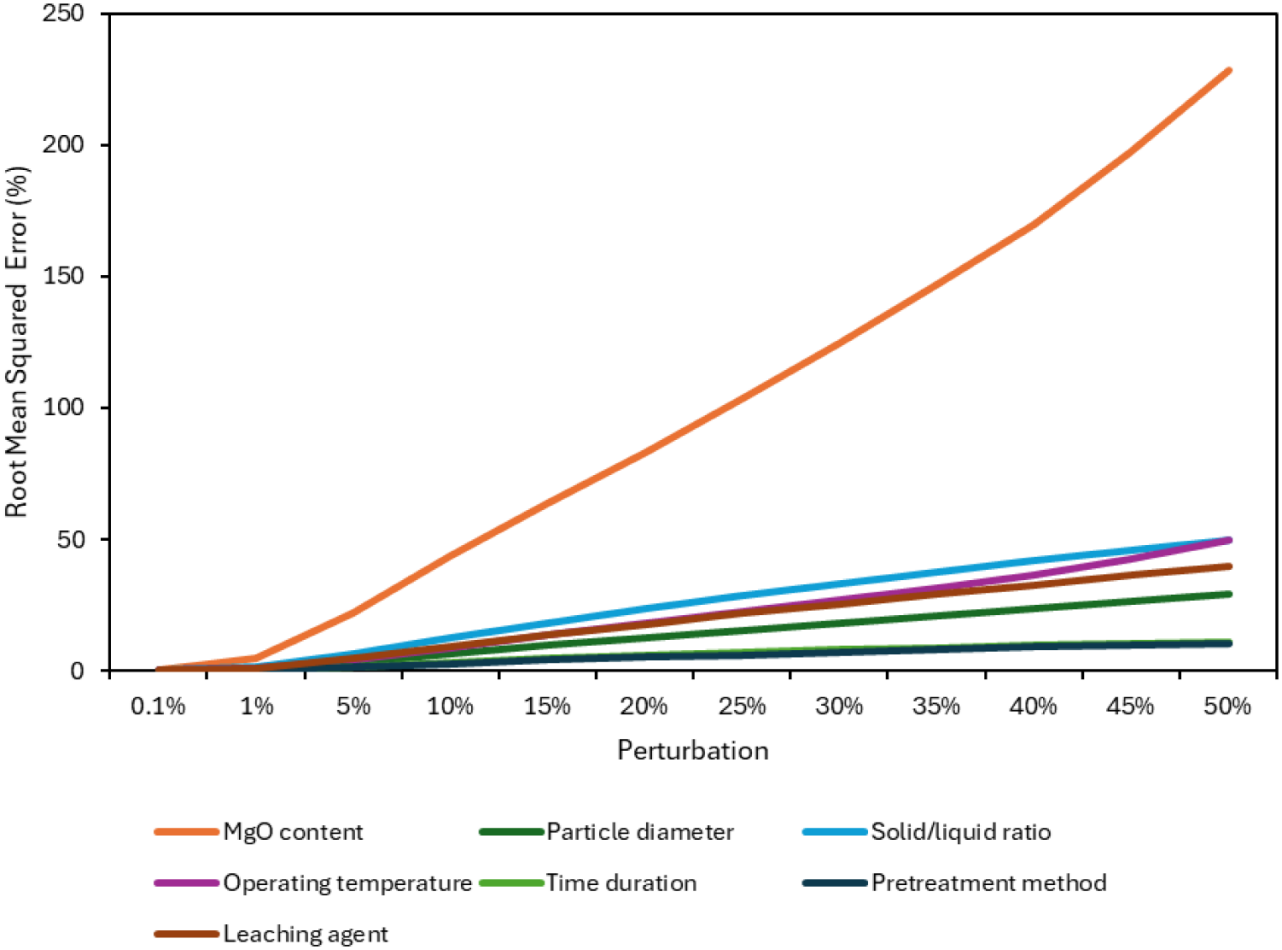
Extraction sensitivity analysis of the model using input perturbation technique.

#### 3.1.2 Full Factorial and Statistical Analysis Results

The weighted average factorial analysis, utilizing modular arithmetic, was conducted prior to the N-way ANOVA analysis. The weighted average study yielded results comparable to ANOVA when using 100% of the collected data due to the unrealistically wide ranges of data categories used. However, with more realistic data ranges, the weighted average method deviated from the more accurate ANOVA method when 80% of the data was used. The ANOVA method was subsequently used, as it was more suited to the data and provided greater accuracy in this case. The weighted average method is more specific to continuous data and focuses on the weight of individual factors, in contrast to ANOVA, where the *F*-value is based on group means and categorical data. The ANOVA method also allows for the determination of statistical significance and the discarding of nonstatistically relevant data points.^69^ Therefore, the results for the weighted average method were not analyzed further. Using ANOVA, the contributing effects of the main and interaction parameters on the extraction efficiency response were evaluated. The analysis was conducted for ranges where 100% (full range) and 80% (optimized range) of the data were used. The results of the ANOVA analysis with 100% and 80% of the data are presented in Tables S3 and S4, respectively.

After discarding the parameters with the *p*-values greater than 0.05, the normalized *F*-values (divided by the sum of all *F*-values) of the remaining main and interaction factors were plotted as shown in Figures 6 and 7.

**Figure 6:**
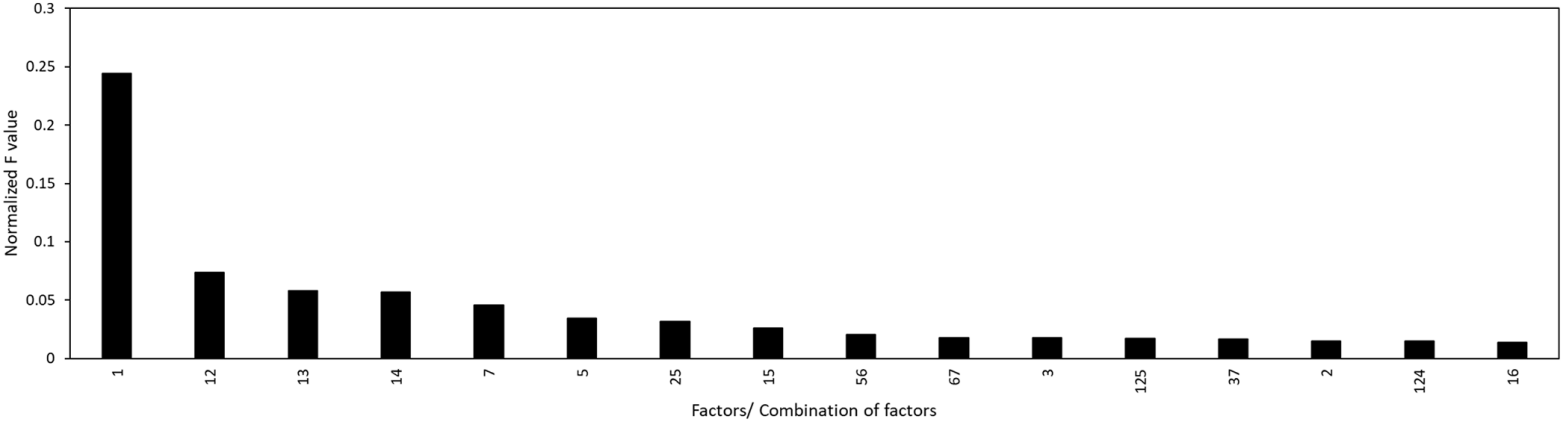
Effect of combined factors on the extraction efficiency with 100% data used for modeling, first 15 factors shown (full image available in the Supporting Information).

**Figure 7:**
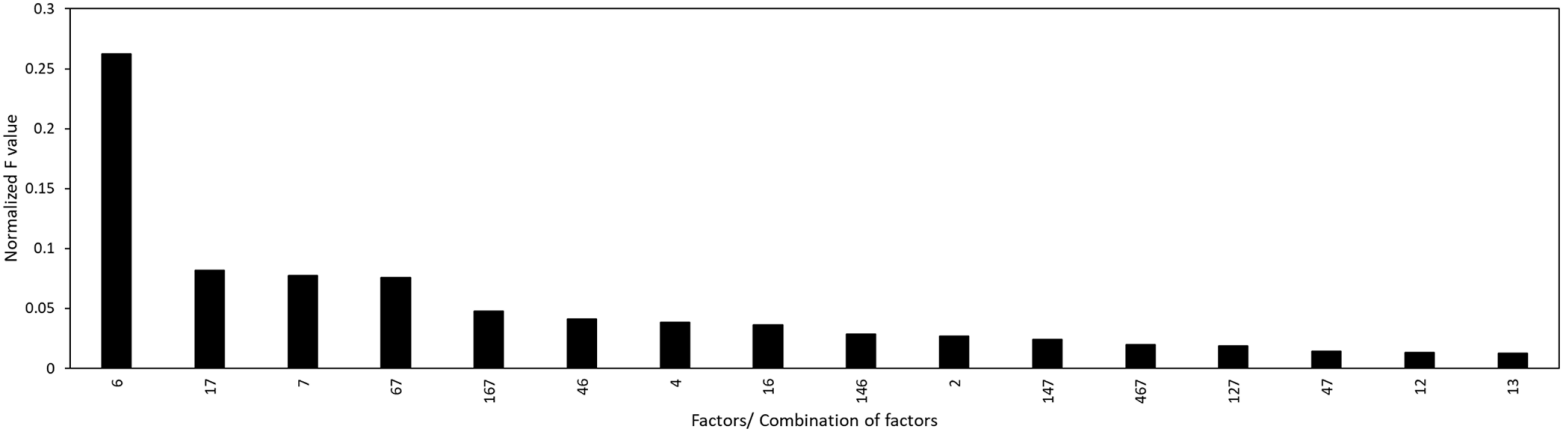
Effect of combined significant factors on the extraction efficiency with 80% data used for modeling, first 15 factors shown (full image available in the Supporting InformationI).

For 100% data used, the significance of the parameters and interaction of parameters on the extraction efficiency as from Figure 6 were in order: Factor 1 > Factors 1,2 > Factors 1,3 > Factors 1,4 > Factor 7 > Factor 5 > Factors 2,5 > Factors 1,5 > Factors 5,6 > Factors 6,7 > Factor 3 followed by all the other combinations. This means that Factor 1 (%MgO content) was the most important determining factor for extraction efficiency, followed by Factors 7 and 5 (extraction agent and time), when wide ranges of data were used. However, it is generally unlikely to encounter such a wide range of operational factors occurring simultaneously. Therefore, the next presented study, which used 80% of the data, was more applicable for the analysis conducted. The ranges used in these were also similar to those found in other parametric studies in the literature, ensuring consistency and comparability of results.^34,55,56,92,95,96,98,99,102,103,130^ For the 80% data used (Figure 7), among the individual influencing factors, pretreatment (Factor 6) had the greatest impact on extraction efficiency, followed by the extraction agent (Factor 7), the temperature (Factor 4), and then the particle size (Factor 2). This means that transitioning from no pretreatment (low level) to microwave pretreatment (high level) would result in the highest significance in extraction efficiency. Factor 5 was the least influential among the individual factors, in contrast to the results seen with the extreme ranges. The interaction of the factors was also observed to have a lower effect on extraction efficiency. The significance of the synergetic effects between factors on the extraction responses follows the order: Factors 1,7 > Factors 6,7 > Factors 1,6,7 > Factors 4,6 > Factors 1,4,6 > Factors 1,4,7 followed by all the other combinations. This observation aligns more closely with literature parametric studies compared to when using 100% of the data. The dominance of pretreatment using 80% of the data highlights its critical role in enhancing the reactivity of solid waste feedstocks for CO_2_ mineralization. This pronounced effect stems from pretreatment’s ability to reactivate alkaline serpentine-based components (e.g., MgO), thereby facilitating extraction and subsequent carbonation. The substantial gap between the first and second ranks underscores pretreatment as the primary driver of efficiency, warranting optimization in process design. From the literature, both heat and microwave pretreatment methods have demonstrated high extraction efficiencies, often exceeding 60%.^48,54,56,82,93,131^ Raschman et al.^132^ reported that calcined serpentine particles smaller than 315 μm exhibited increased magnesium dissolution rates when treated with different acids. Specifically, the dissolution rate increased by 30 times with HCl, 125 times with acetic acid, and 165 times with ammonium chloride, compared to noncalcined serpentine. Sanna et al.^56^ reported similar observation, noting an approximate 35% improvement in extraction efficiency for thermally pretreated serpentine. Ranked second significant synergy of extraction agent and MgO content (Factors 1,7) suggests that the effectiveness of the extraction agent (e.g., acid type or concentration) is highly dependent on the MgO content of the feedstock, likely due to MgO’s role as a key reactant in forming soluble magnesium ions for carbonation. The proximity of this interaction to the main effect of the extraction agent (rank 3) emphasizes their combined importance in process optimization. The importance of the extraction agent has also been highlighted in various studies. Stronger acid extraction agents, such as HCl, typically result in higher extraction efficiency compared to weaker extraction agents like NH_4_Cl.^94,96,97,99^ ^,^ The ranked fourth binary interaction between pretreatment and the extraction agent (Factors 6,7) indicates a moderate synergistic effect on extraction efficiency. This interaction likely reflects the enhanced dissolution of reactive components when pretreatment (e.g., thermal activation) is combined with an optimized extraction agent. Its significance, closely aligned with ranks 2 and 3, suggests that coordinating pretreatment and extraction agent selection could further improve efficiency in CO_2_ mineralization processes. A less pronounced significance of the tertiary interaction among pretreatment, MgO content, and extraction agent (Factors 1,6,7) (rank 5) demonstrates the combined influence of these factors creates a complex interplay, where the noticeable difference between ranks 4 and 5 indicates that higher-order interactions contribute less to variance than primary factors and their binary interactions. The binary interaction between pretreatment and temperature (Factors 4,6), ranked sixth, suggests that temperature modulates the effectiveness of pretreatment to a moderate degree. For instance, higher temperatures may enhance the kinetics of extraction following pretreatment, but this effect is less significant than the main effects or higher-ranked interactions. Temperature’s main effect, ranked seventh, indicates a moderate influence on extraction efficiency, likely due to its role in accelerating reaction kinetics during extraction or carbonation. Its lower ranking compared to the other main factors, including pretreatment and extraction agent, suggests that temperature plays a supporting role, with its effect being less pronounced in the absence of optimized pretreatment or the other extraction conditions. Sanna et al.^56^ conducted a parametric study on heat treated natural serpentine, varying factors such as the solid/liquid ratio, particle size, operating temperature, and NH_4_HSO_4_ concentration. They found that the operating temperature was the most influential parameter, leading to a 50% improvement in extraction efficiency when the temperature was increased from 50 to 140 °C. Gao et al.^94^ observed a similar trend, where increasing the temperature from 70 to 104 °C resulted in an increase in magnesium extraction efficiency from 11% to approximately 22% for natural heat-treated serpentine. This increase in magnesium extraction efficiency is significantly higher than the improvements observed with the other examined parameters, such as particle size, solid:liquid ratio, NH_4_Cl concentration, and time interval. Arce et al.^92^ conducted a parametric study on the extraction optimization of mining wastes, examining the effects of temperature, particle size, and HCl concentration. They found that the temperature had the most significant impact on extraction efficiency. Specifically, when the temperature was increased from 25 to 100 °C, the extraction efficiency improved significantly. At 100 °C, with a particle size of 300 μm and an HCl concentration of 1 M, an extraction efficiency of 97% was achieved. The binary interaction between pretreatment and MgO content (Factors 1,6; rank 8) reflects a moderate but less significant synergistic effect compared to the MgO content–extraction agent interaction (Factors 1,7). While pretreatment enhances the overall reactivity of the feedstock, its effect on MgO dissolution is less dependent on MgO content variations than the extraction agent’s effect. The extraction agent directly contributes to selective extraction of Mg^2+^ ions, making its interaction with MgO content more critical, whereas pretreatment influence on the extraction reactivity enhancement is less specific to MgO content. This explains the gap in rankings and highlights the extraction agent’s stronger influence on MgO reactivity in the extraction phase. The tertiary interaction among MgO content, temperature, and pretreatment (Factors 1,4,6) (rank 9) has a detectable but less significant effect on extraction efficiency compared to Factors 1,6. This tertiary interaction captures the combined influence of the given parameters, where pretreatment enhances feedstock reactivity, higher MgO content provides more reactive material, and temperature accelerates extraction kinetics. Thereby, this lower significance suggests that the temperature’s role is secondary and its additional effect in this synergy is marginal, contributing less than the MgO content and pretreatment interaction alone. There was a small difference between the significance of Factors 1, 4, 6 and 2 (particle size with rank 10) suggesting that the impact of particle size is overshadowed by the other main factors including pretreatment, extraction agent, and MgO content and is less critical than other factors. It is worth mentioning that the binary interaction between MgO content and temperature (Factors 1,4) demonstrated a very low significance on extraction efficiency (out of the first 10 ranks). It implies that temperature’s effect on MgO dissolution is not strongly dependent on MgO content alone and it is less pronounced in the absence of the other factors such as pretreatment. Due to the significant influence of pretreatment, temperature, particle size, and extraction agent on extraction efficiency, their surface plots were generated to illustrate the effects of these factors, as shown in Figure S2. These plots clearly illustrate the dramatic improvement in performance when the extraction agent (Factor 7) and pretreatment (Factor 6) parameters are optimized compared to when they are not. This significant change is more pronounced than the effects of other parameters, which is reflected in the high *F*-value shown in Figure 7. As specifically shown in Figure S2B–F, the extraction efficiency exhibits a generally high response (indicated by the green-to-red hue variation) when the pretreatment factor is optimized, moving from 0 to 2. This response is more pronounced than that of other parameters. Overall, the prioritization of different parameters depends on the ranges of those parameters used. However, Figure 7 provides a sufficient estimation of the priority of different parameters to maximize extraction efficiency as 80% of the data ranges used in the optimization are covered within this range.

### 3.2 CO_2_ Mineralization Analysis

#### 3.2.1 Literature Data ANN Modeling

A similar methodology was used for developing an ANN model based on the mineralization data gathered from the literature. For solely direct carbonation, the average *R* value and RMSE values were 0.9943 and about ±0.008487 g of CO_2_-sequestered/g-serpentine. For solely indirect carbonation, the average *R* value and RMSE values were 0.883 and about ±0.03631 g of CO_2_-sequestered/g-serpentine. The correlations between the training, testing, and validation data with the model are shown in Figure 8A,B.

**Figure 8:**
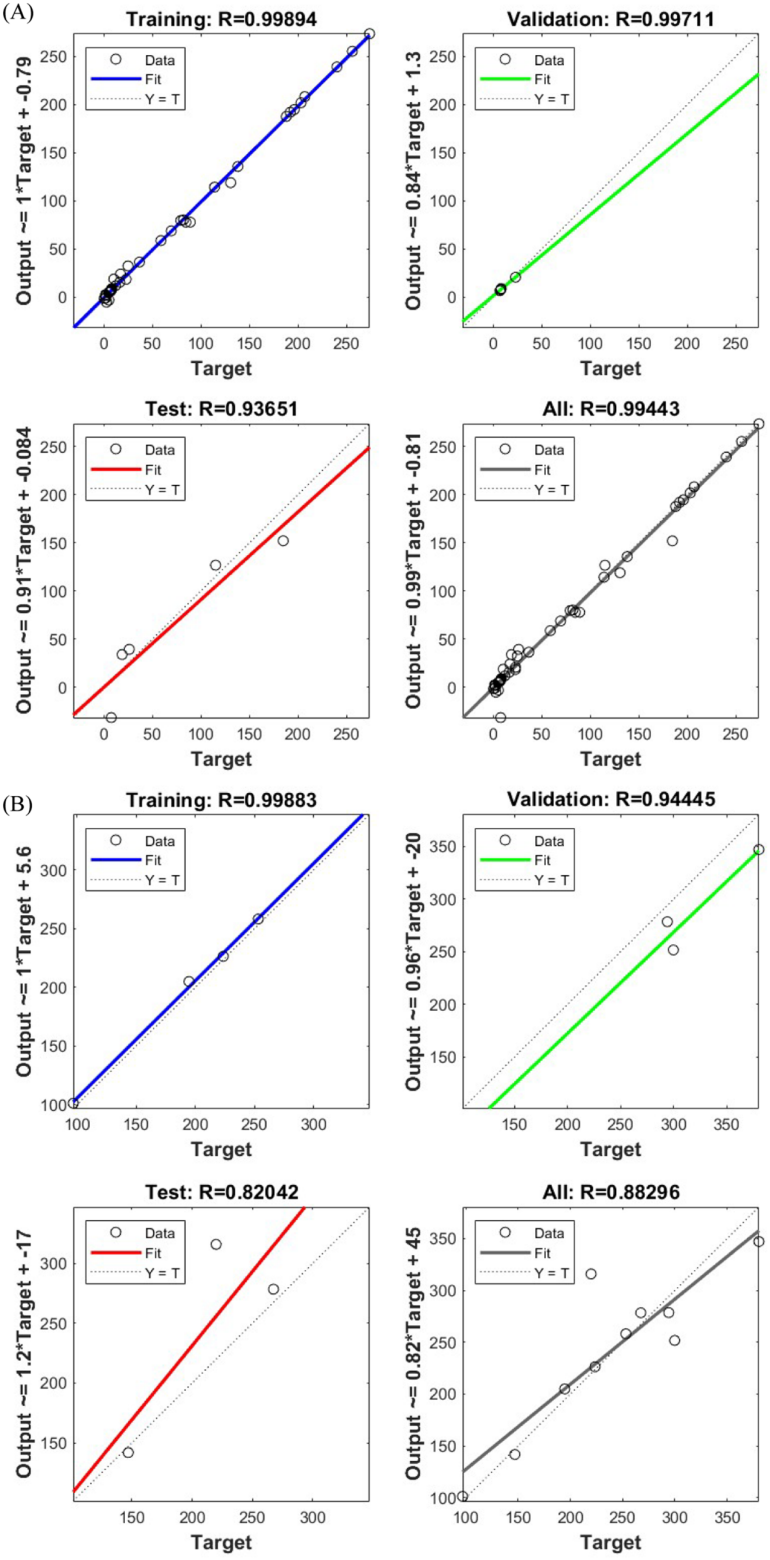
Model correlation performance for CO_2_ mineralization. Mineralization model (A) direct carbonation and (B) indirect carbonation.

A sensitivity analysis was also performed on the mineralization model developed for the existing experimental data (Figure 9), similar to the input perturbation algorithm described in the extraction section. For direct carbonation (Figure 9A), the model shows the highest sensitivity to variations in the CO_2_ pressure (Factor 6) and reaction pH (Factor 5). Factor 2, the particle size, is the most inconsequential factor to the model’s performance compared to the other factors. For indirect carbonation (Figure 9B), the model is most sensitive to Factors 3 and 5, namely, the reaction pH and the use of a carbonation assisting agent. Factor 4 (CO_2_ pressure) had the least effect.

**Figure 9:**
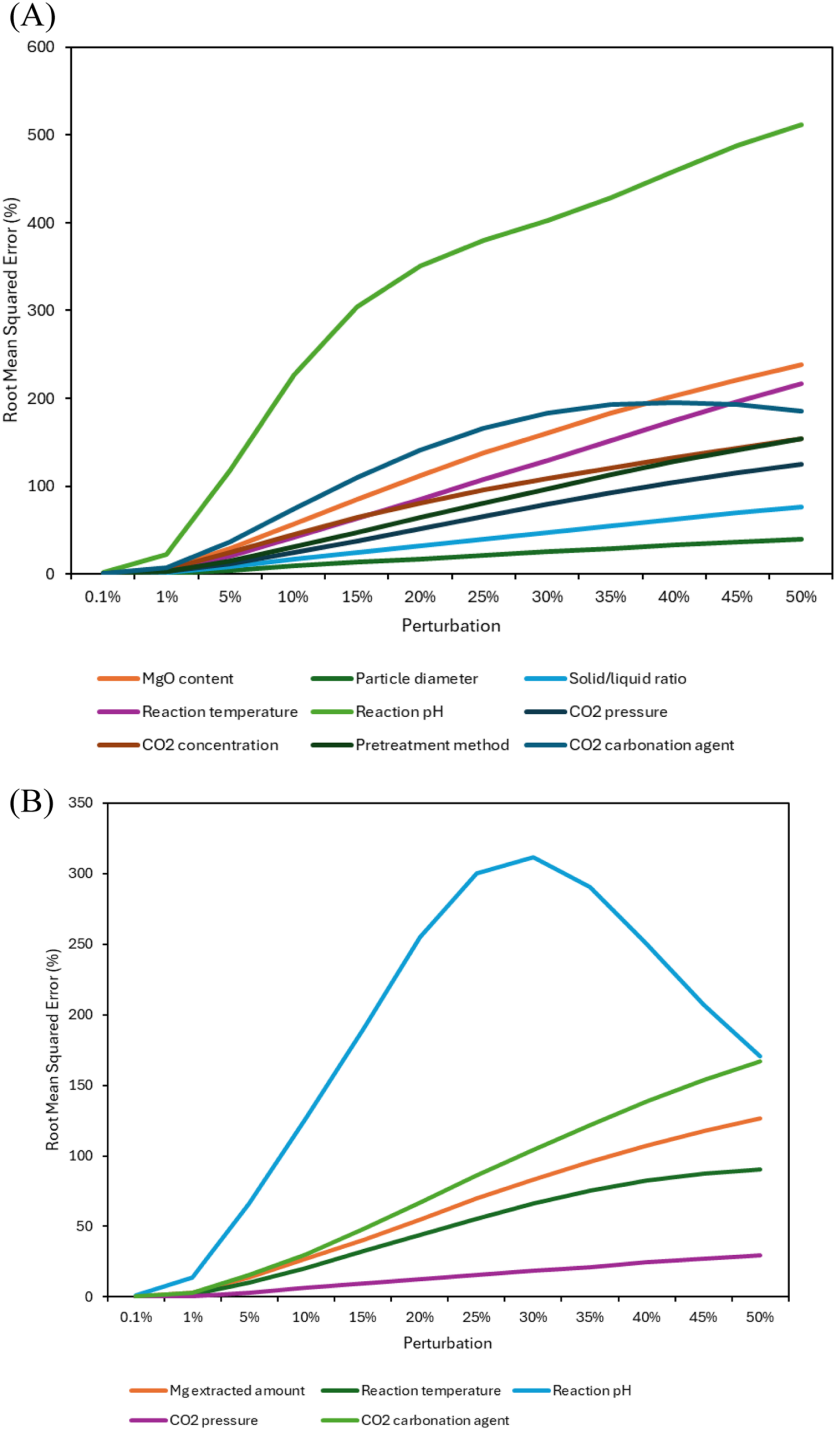
Sensitivity analysis of the mineralization model using the input perturbation method. (A) Direct mineralization model and (B) indirect carbonation.

#### 3.2.2 Full Factorial and Statistical Analysis Results

A similar ANOVA analysis methodology was applied to determine the effects of main and interaction parameters on the CO_2_ mineralization process with both the extreme data range (100% of the data points) and the conservative data range (80% of the data points). The results of the ANOVA analysis for direct and indirect CO_2_ carbonations are presented in Tables S5–S8. After discarding parameters with a *p*-value greater than 0.05, the normalized *F*-values of the remaining main and interaction factors are plotted in Figures 10 and 11. The figures show the first 100 factors with the highest normalized *F*-values.

**Figure 10:**
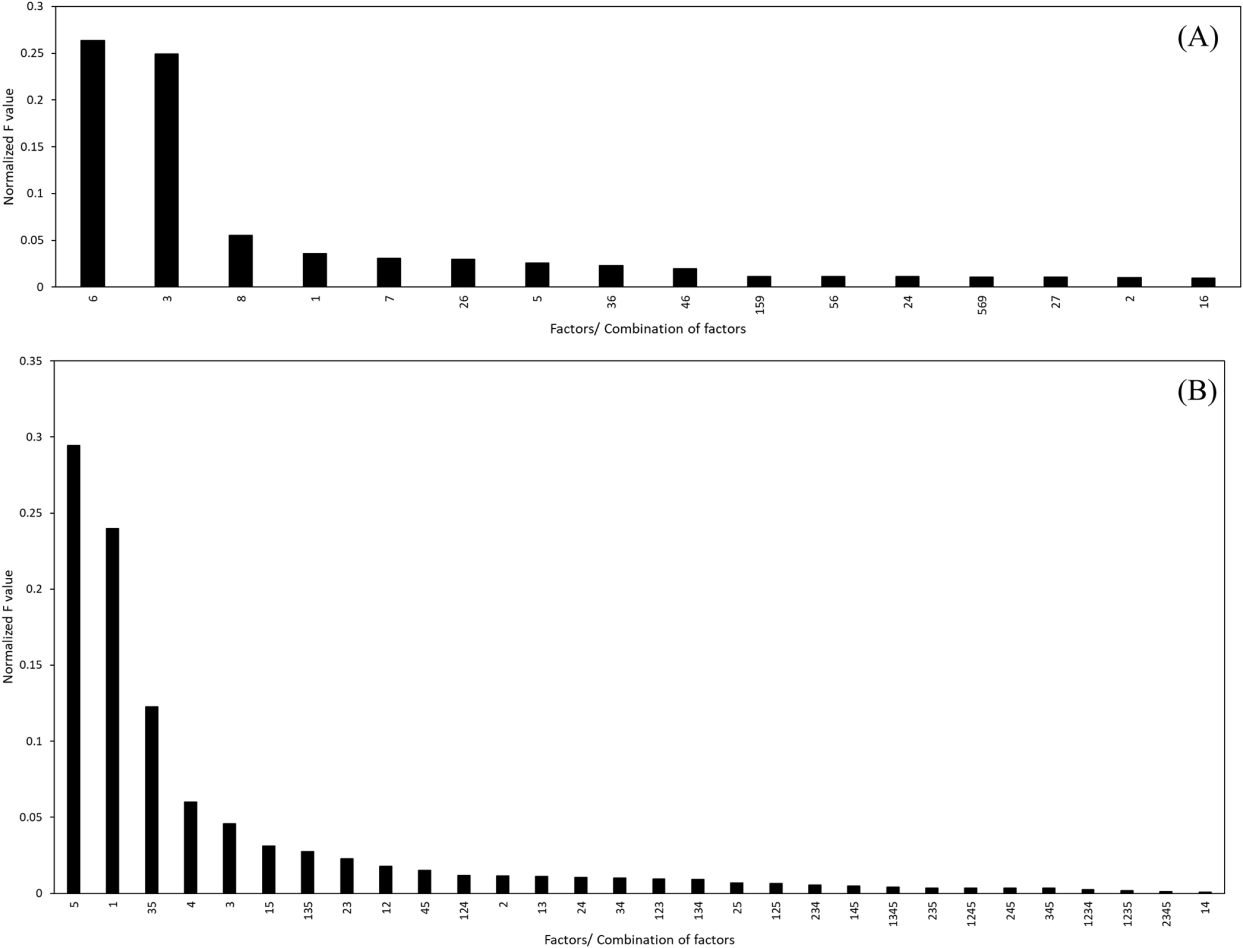
Effect of combined factors on CO_2_ sequestration capacity with 100% data used for modeling. (A) Direct carbonation, the first 15 factors shown (full image available in the Supporting Information) (B) indirect carbonation (full image available in the Supporting Information).

**Figure 11:**
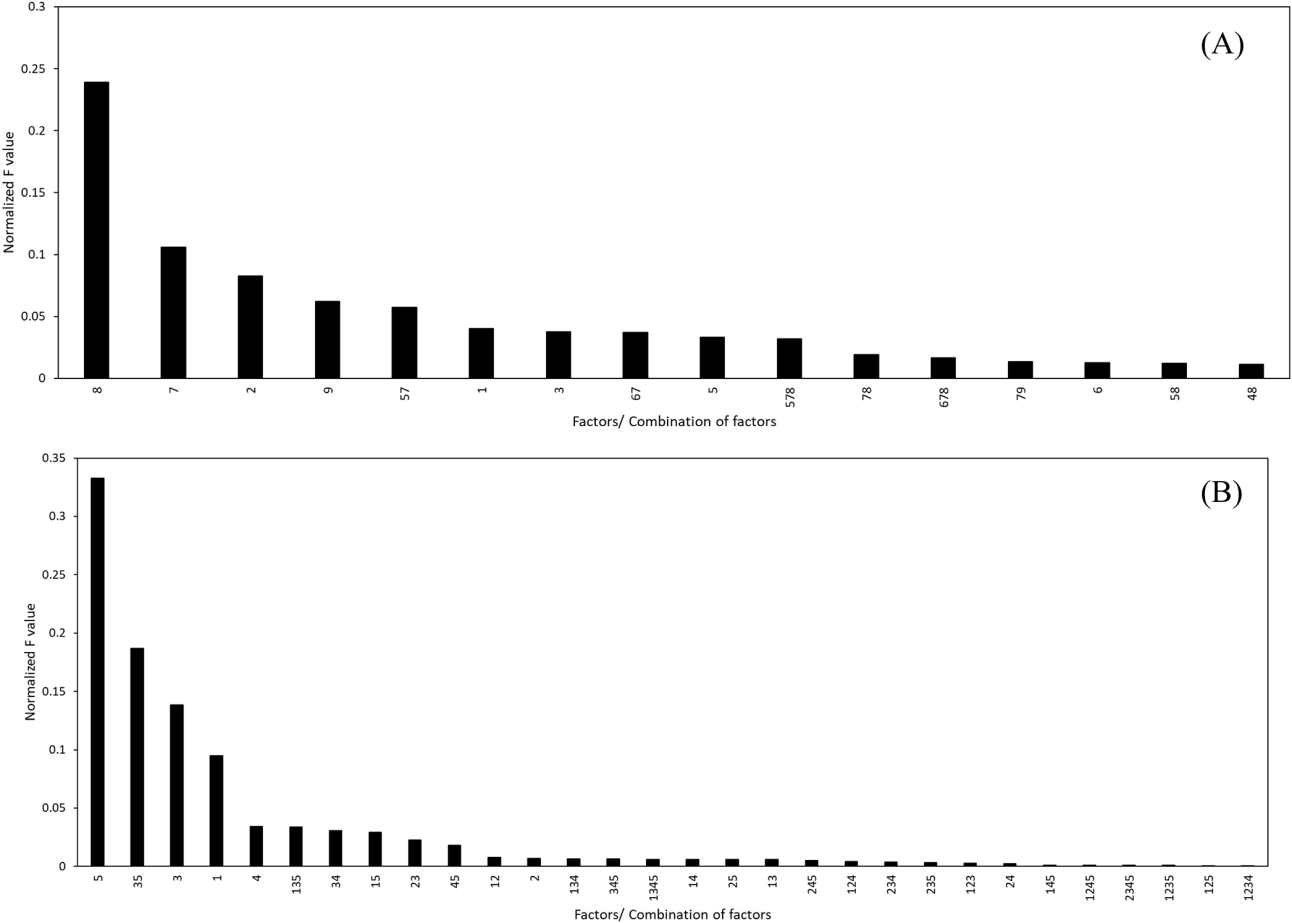
Effect of combined factors on CO_2_ sequestration capacity with 80% data used for modeling. (A) Direct carbonation—the first 15 factors shown (full image available in the Supporting Information) (B) Indirect carbonation (full image available in the Supporting Information).

For direct carbonation with 100% data used (Figure 10A), the effect of individual and combined factors on the CO_2_ sequestration capacity was in the order: Factor 6 > Factor 3 > Factor 8 > Factor 1 > Factor 7 > Factors 2,6 > Factor 5 > Factors 3,6 > Factors 4,6 > Factors 1,5,9 ≈ Factors 5,6 ≈ Factors 2,4 ≈ Factors 5,6,9 ≈ Factors 2,7 ≈ Factor 2 > Factors 1,6, followed by other combination and individual factors. This means that the CO_2_ gas pressure (Factor 6) was the most important determining factor for direct CO_2_ carbonation, followed by the solid/liquid ratio (Factor 3), feedstock pretreatment (Factor 8), and Mg content at feedstock (Factor 1), when the wide ranges of data were used. As discussed at extraction section, it is more realistic to develop analysis using 80% of data range, excluding outliers. With 80% data used (Figure 11A), the most significant factors for mineralization are in the following order: Factor 8 > Factor 7 > Factor 2 > Factor 9 > Factors 5,7 > Factor 1 > Factor 3 > Factors 6,7 > Factor 5 > Factors 5,7,8 > Factors 7,8 > Factors 6,7,8 > Factors 7,9 ≈ Factor 6 ≈ Factors 5,8 ≈ Factors 4,8, ≈ Factors 1,8,9 > Factors 1,6, followed by other combinations. Pretreatment of the feedstock solid emerged as the most significant factor (rank 1), with a greatly higher significance compared to all other factors. This primacy underscores pretreatment’s critical role in enhancing feedstock reactivity for carbonation. Techniques such as thermal activation increase the surface area and liberate reactive Mg-based alkaline components, promoting their extraction and reaction with the CO_2_ in solution. The substantial significance gap highlights pretreatment as the primary driver, necessitating its optimization to maximize the CO_2_ sequestration capacity. CO_2_ concentration in the reacting gas stream ranked second, significantly influencing sequestration capacity, though less than pretreatment. High CO_2_ concentrations (e.g., near 100 vol%) enhance carbonation kinetics by increasing CO_2_ availability at the feedstock surface, promoting carbonate formation. This effect is crucial in direct mineralization, where CO_2_ interacts with reactive components. The notable significance over the following ranks emphasizes the importance of elevated CO_2_ levels for efficient carbonation. Particle size ranked third, with a clear impact on sequestration capacity. Smaller particle sizes (e.g., <75 μm) increase the reactive surface area, enhancing dissolution rate and reaction kinetics during carbonation. This effect is pronounced in direct mineralization, where carbonation mainly occurs at solid–liquid interface.^20^ The significance advantage over lower ranks reinforces particle size as a key factor, although it is secondary to pretreatment and CO_2_ concentration. The carbonation assistant agent (e.g., NaHCO_3_) ranked fourth, contributing moderately to the sequestration capacity. These agents enhance carbonation by acting as a carbon source or pH buffer, maintaining the dissolution rate of metal ions, and facilitating the dissolution of CO_2_ and its reaction with metal ions. Its significance, slightly lower than particle size, suggests a supportive role in optimizing solution chemistry, critical in aqueous direct mineralization systems. The binary interaction between system pH and CO_2_ concentration ranked fifth, with a significance close to that of the carbonation assistant agent. This synergy arises because pH influences CO_2_ speciation (e.g., HCO_3_^–^, CO_3_^2–^) and metal ion solubility, while high CO_2_ concentrations increase carbonate availability. The proximity to rank 4 indicates that optimizing pH alongside the CO_2_ concentration enhances carbonation efficiency, particularly in aqueous systems. MgO content in the feedstock, ranked sixth, has a modest effect. MgO provides reactive Mg^2+^ ions for carbonation, but its lower ranking suggests that its availability is less limiting compared to physical factors (pretreatment, particle size) and CO_2_ availability. The solid/liquid ratio, ranked seventh, has a moderate impact, with significance comparable to that of ranks 6. The solid/liquid ratio affects ion concentration and mass transfer during carbonation, but its low significance indicates that it is secondary to factors controlling CO_2_ reaction kinetics and feedstock reactivity. The binary interaction between the CO_2_ pressure and the CO_2_ concentration, ranked eighth, reflects a modest synergy, with almost similar significance to ranks 6 and 7. Higher CO_2_ pressures enhance the CO_2_ solubility, complementing high CO_2_ concentrations to drive carbonation. Its lower ranking suggests that this synergy is less critical than main effects or the pH–CO_2_ concentration interaction (Factors 5,7). Reaction pH, ranked ninth, has less significance than other primary factors, such as feedstock preparation and CO_2_ gas concentration. While pH affects CO_2_ speciation and ion solubility, its low significance suggests that the carbonation assistant agent and CO_2_ concentration interaction already optimize the pH, reducing its independent significance. The tertiary interaction among reaction pH, CO_2_ concentration, and pretreatment, ranked tenth, has almost similar significance to reaction pH alone (rank 9). This complex synergy likely reflects pretreatment’s enhancement of reactive ion availability, modulated by pH and CO_2_ concentration, but its low ranking indicates its limited additional variance compared to main effects and binary interactions. The binary interaction between the CO_2_ concentration and pretreatment, ranked 11th, has a low significance. This minor synergy suggests that pretreatment’s enhancement of feedstock reactivity is largely independent of CO_2_ concentration variations, as pretreatment’s primary effect is captured in its main effect (rank 1). The tertiary interaction among the CO_2_ pressure, the CO_2_ concentration, and the pretreatment, ranked twelfth, has a minimal effect, closely aligned with rank 11. This synergy reflects the combined influence of CO_2_ availability and pretreatment, but its low ranking indicates that it contributes little additional variance compared to the main effects and simpler interactions. Factors and interactions beyond rank 12, such as the CO_2_ pressure and reaction temperature, exhibit low significance. CO_2_ pressure’s low ranking of the CO_2_ pressure suggests that solubility enhancements are less critical in direct mineralization, where carbonation occurs at the feedstock surface, mainly controlled by physical factors due to simultaneous extraction and carbonation. Low significance of temperature and its related interactions indicate that thermal effects are secondary. Most of direct CO_2_ mineralization studies available in the litearture investigated reaction performnace by changing critical parameters such as CO_2_ pressure and concentration, or operating tempearture, while other parameters like particle size and reaction pH were kept constant. The relative importance of those mentioned factors has already been addressed in the literature.^104,133^ Gadikota et al.^104^ found that increasing the CO_2_ partial pressure from 6.4 to 13.9 MPa improved the extent of carbonation by 46% when uisng optimal temperature, reaction time, and carbonation assisting agent concentration (NaCl and NaHCO_3_) with a pure CO_2_ source in the direct carbonation of olivine. Increasing the temperature from 90 to 185 °C resulted in an 83% improvement in carbonation at a constand CO_2_ pressure of 13.9 MPa and other identical conditions. It is important to note that this parametric study was conducted on very small samples (under 37 μm). The results confirmed the importance of both the reaction temperature and the CO_2_ pressure when all other parameters were optimized. Pronost et al.^133^ noted an increase in the amount of CO_2_ sequestered from 1.94 g/kg of serpentine to 22.46 g/kg of serpentine when direct carbonation was performed at 33 °C, and the CO_2_ concentration and pressure were increased from 4% and 0.004 MPa to 23% and 0.023 MPa.^133^ It was also noted that the incorporation of pretreatment led to an increased amount of leached Mg.^49,57,79,80,81,87,136^ ^,^ Therefore, it can be inferred that this would also lead to an increased amount of sequestered CO_2_ sequestered. Higher carbonation efficiencies were observed for previously thermally activated serpentine.^81,82^ Lastly, using a strong base carbonation assisting agent generally resulted in a higher amount of CO_2_ sequestered, around or above 200 g/kg-mineral,^97,104,112,117,130^ compared to below 100 g/kg-mineral when no carbonation agent was used.^14,122,133^ This is in agreement with the high normalized *F*-value observed for the carbonation assisting agent. Additionally, a high pH generally coincides with the use of a carbonation assisting agent, which is required for carbonation to occur after extraction (pH swing process).^7,53,98,102,127,128^

For indirect carbonation with 100% data (Figure 10B), the effect of individual and combined factors on the CO_2_ sequestration capacity was in the order: Factor 5 > Factor 1 > Factors 3,5 > Factor 4 > Factor 3 > Factors 1,5 > Factors 1,3,5 > Factors 2,3 > Factors 1,2, followed by other combination and individual factors. This indicates that the carbonation assistant agent (Factor 5) was the most critical determinant for indirect CO_2_ carbonation. This was followed by extracted Mg concentration alone (Factor 1), synergistic interaction between factors reaction pH and carbonation assistant agent (Factors 3,5), and the CO_2_ pressure (Factor 4), when considering the wide range of data. Considering the more realistic data range (80%) (Figure 11B), the most significant factors for mineralization are in the following order: Factor 5 > Factors 3,5 > Factor 3 > Factor 1 > Factor 4 ≈> Factors 1,3,5 > Factors 3,4 ≈ Factors 1,5 > Factors 2,3 > Factors 4,5 > Factors 1,2 > Factor 2, followed by other combination and individual factors. The carbonation assistant agent (e.g., NaOH and NaHCO_3_) emerged as the most significant factor, with a substantial significance gap. Agents like NaOH adjust pH and favor carbonate formations, while NaHCO_3_ provides carbon and has buffering effect by forming complexes. This primacy underscores the agent’s critical role in optimizing solution chemistry for aqueous carbonation. The binary interaction between solution pH and the carbonation assistant agent (Factors 3,5), ranked second, is highly significant but notably less so than the carbonation agent alone. This synergy reflects the agent’s modulation of pH, enhancing CO_2_ speciation (e.g., CO_3_^2–^) and ion reactivity at optimal pH levels (e.g., 8–11), reinforcing solution chemistry’s importance. Solution pH (Factor 3), ranked third, has a strong effect, with a sharp reduction in significance from rank 2. pH governs CO_2_ speciation and metal ion solubility, critical for carbonate precipitation (e.g., MgCO_3_, CaCO_3_). Its high ranking emphasizes the need for precise pH control, although it is secondary to the carbonation agent and their interaction. Initial Mg concentration in the leachate, ranked fourth, significantly influences sequestration capacity with a further sharp reduction in significance. Higher Mg^2+^ concentrations enhance MgCO_3_ formation, but its lower ranking suggests sufficient Mg^2+^ availability, making solution chemistry more limiting. CO_2_ pressure (Factor 4) and the ternary interaction of Mg concentration, pH, and carbonation assistant agent (Factors 1,3,5), both ranked fifth, have nearly equal significance. CO_2_ pressure enhances the CO_2_ solubility, while the ternary interaction reflects combined effects of Mg^2+^ availability, pH-driven speciation, and agent-enhanced reactivity. Their moderate significance indicates their supportive roles. The binary interaction between solution pH and CO_2_ pressure (Factors 3,4) and the interaction of Mg concentration and carbonation assistant agent (Factors 1,5), both ranked sixth, are nearly equal in significance to. This synergy reflects pH’s influence on CO_2_ speciation, modulated by CO_2_ solubility, but is less critical than higher-ranked factors. The binary interaction Factors 1,5 has a comparable effect to Factors 3,4, enhancing the Mg^2+^ reactivity through agent-driven solution chemistry. Its moderate significance suggests a secondary role. The binary interaction between reaction temperature and solution pH (Factors 2,3), ranked seventh, shows a reduced effect, reflecting temperature’s kinetic enhancement modulated by pH-dependent speciation. Its lower ranking indicates a supportive but noncritical role. The binary interaction between CO_2_ pressure and the carbonation assistant agent (Factors 4,5), ranked eighth, has a modest effect, suggesting that the agent’s effectiveness is less dependent on CO_2_ pressure variations. The binary interaction between Mg concentration and reaction temperature (Factors 1,2) and reaction temperature alone (Factor 2), both ranked ninth, has nearly equal, minimal significance. Temperature’s low impact suggests that kinetic enhancements are less critical than solution chemistry and CO_2_ solubility. The carbonation step of indirect CO_2_ mineralization is driven by solution chemistry (carbonation assisting agent, pH, and their interaction), with initial Mg concentration and CO_2_ pressure as moderate factors and temperature exerting minimal influence. Compared with direct CO_2_ mineralization, where pretreatment and CO_2_ concentration dominate, indirect carbonation emphasizes aqueous reaction conditions. These findings guide optimization by prioritizing carbonation agents, pH control, and CO_2_ pressure for indirect processes, enhancing the sequestration of CO_2_ from solid waste leachates. The surface plots corresponding to the interaction between critical factors and their influence on the CO_2_ sequestration capacity are presented in Figures S3 and S4, for direct and indirect carbonations, respectively. For direct carbonation, the dependence of the amount of CO_2_ mineralized on the CO_2_ concentration in the feed gas is clearly recognized (Figure S3A,C,D). There is a significant improvement in the amount mineralized when this percentage is over 70%, even compared to other parameters, as shown by the color contrasts. The use of an effective carbonation agent also has the potential to improve the amount of mineralized CO_2_ (Figure S3B,D)). However, it is evident that optimizing the CO_2_ concentration is more crucial than using a more effective carbonation agent. The effect of the pretreatment is also slightly visible, as shown in Figure S3C, especially when transitioning from heat pretreatment to microwave. However, since this figure represents only the ANN model outputs derived from 80% of the experimental data collected, it overemphasizes the CO_2_ concentration effect while under-representing the pretreatment influence. This limitation reinforces the necessity of conducting an ANOVA to accurately assess the relative importance of each experimental factor. For indirect carbonation, the generated surface plots are more extreme and limited due to the insufficient literature data available. Additionally, the model might not accurately represent the data, especially with a limited number of data points. This leads to an overestimation of the amount of CO_2_ sequestered while varying the influencing factors. The influence of changing and the necessity to optimize the pH from mid to high levels (9–11) on carbonation performance, while varying other parameters such as Mg concentration, CO_2_ pressure, and carbonation assisting agents, is illustrated in Figure S4A,C,D. The use of an effective carbonation assisting agent is shown to be important to a lesser extent, as shown in Figure S4D. The effect of optimizing (increasing) the Mg concentration extracted into solution is also evident in Figure S4A,B. Lastly, another major effect highlighted is the necessity of optimizing the CO_2_ pressure, as illustrated in Figure S4C,E.

While the 3^k^ full factorial design was employed to systematically evaluate the main and interaction effects of influencing process parameters, we recognize that some hypothetical parameter combinations (e.g., high temperature, low pH, and microwave treatment for direct carbonation) may be impractical or physically infeasible under real-world conditions. To address this, only experimentally validated data from the literature were used to train the ANN model, ensuring that predictive outputs remained grounded in a physically realistic behavior. The factorial design was used as a theoretical framework to explore parametric sensitivity and scenario prediction with the understanding that engineering constraints and practical limitations must be considered before implementation. In future work, integrating constraint-based filtering into the design space could further refine the prediction scope. Additionally, the developed ANN model predicts extraction efficiencies and CO_2_ sequestration capacities using literature-based data sets. While these models effectively capture the cumulative influence of process parameters on overall performance, they do not explicitly account for reaction kinetics or dynamic system behaviors. As such, the approach is best suited for process optimization and parametric analyses conducted on a steady-state or nontime-dependent basis. Future work incorporating time-series data could enhance the model’s capability to reflect kinetic phenomena and identify rate-limiting steps.

### 3.3 Other Considerations

Other than the factors discussed above, additional considerations must be made when designing CO_2_ mineralization experimental studies. One major consideration is the conditions under which magnesium carbonate phases can be formed, as this determines the quality and value of the MgCO_3_ product and affects the competitiveness of the carbonation process. Additional parameters to consider include the regenerability of the extraction and carbonation agents, the corrosivity and toxicity of the agents used, the environmental and life cycle impacts of the process, and the operation costs. All of these variables are crucial for selecting the appropriate carbonation process.

#### 3.3.1 Magnesium Carbonate Phases

There are generally five semistable and stable phases of magnesium carbonate (MgCO_3_) differentiated by their chemical and physical properties: anhydrous magnesite (MgCO_3_), nesquehonite (MgCO_3_·3H_2_O), lansfordite (MgCO_3_·5H_2_O), artinite (MgCO_3_·Mg­(OH)_2_·3H_2_O), and hydromagnesite ((MgCO_3_)_4_·Mg­(OH)_2_·4H_2_O).^7,129,138^ ^,^ Among these, magnesite, nesquehonite, and hydromagnesite phases are the most common Mg-bearing compounds reported in the literature as products of CO_2_ carbonation processes.^7,129^ ^,^ The most stable form of magnesium carbonate is anhydrous magnesite,^125,135,136^ which is also the most efficient carbonate product for CO_2_ sequestration due to its 1:1 Mg to CO_2_ molar ratio.^134^ The other hydrated forms of magnesium carbonate are prone to dehydration when the temperature is elevated. Magnesite is typically hard to form at ambient temperature and pressure; instead, hydromagnesite and nesquehonite are usually formed. Nesquehonite can be precipitated from Mg^2+^-containing aqueous solutions at room temperature to 52 °C and at moderate CO_2_ partial pressures (ambient to 1 bar).^137,138^ ,^139–145^ At temperatures above approximately 50–52 °C and low pressures, nesquehonite begins to transform into sphere-like sheets of hydromagnesite.^139,140,142,146^ This chemical alteration can usually occur either directly from nesquehonite to hydromagnesite or via a transitory mineral, dypingite.^53,140,145,147^ The chemical composition of dypingite and hydromagnesite is identical; however, their structures differ in terms of hydration levels.^138^ Magnesite, on the other hand, can be synthesized either by direct precipitation from a carbonation solution at high CO_2_ pressure or through the transformation of hydromagnesite to magnesite over time.^148^ Wolf et al.^149^ observed the direct precipitation of magnesite from the carbonation of raw serpentine at 150 °C and a CO_2_ partial pressure of 15 MPa in a NaCl–NaHCO_3_ system. Prigiobbe et al.^154^ observed the direct precipitation of magnesite from a MgCl_2_–Na_2_CO_3_ solution in the presence of supercritical CO_2_ at a partial pressure of 10 MPa and temperatures of 90 and 120 °C. Sayles et al.^137^ investigated the precipitation of magnesite from a MgCl_2_ solution and found that at low CO_2_ pressures (3.16 × 10^–5^ to 0.1 MPa) and temperatures below approximately 150 °C, the transition of hydromagnesite to magnesite occurs over the course of days. At higher temperatures (above 150 °C), the transition of hydromagnesite to magnesite typically occurs within hours. Zhang et al.^151^ observed a similar phenomenon in the precipitation of magnesium carbonate from hydromagnesite in a saturated NaCl solution at elevated temperatures and atmospheric pressure. They found that measurable magnesite precipitation required days to weeks at temperature below 200 °C (e.g., 25 days for 94% transformation at 110 °C and 20 h for 91% transformation at 150 °C). For temperatures above 200 °C, up to 96% of the hydromagnesite was converted to magnesite within 2.5 h. The authors also noted that at temperatures of room temperature to 110 °C, the transformation was too slow to record within 25 days. This slow transformation is due to the additional reaction of MgO present in hydromagnesite with CO_2_.^151^ Therefore, the transformation can be optimized at temperatures above 100 °C (to facilitate dehydration) and either significant CO_2_ partial pressures (above 100 bar) or long reaction times (about a month).^125,150–154^ Hänchen et al.^125^ reported a similar observation, noting a transformation time of 5–15 h when precipitating magnesite from a MgCl_2_ solution in the presence of CO_2_ and Na_2_CO_3_ at 120 °C, and a CO_2__2_ pressure of 3 bar. Overall, the transition of different magnesium carbonate phases is primarily temperature-dependent and does not rely only on the supersaturation level of the parent solution.^145,154^

Beyond the previously mentioned temperature conditions, it was reported that at temperatures between 0 and 10 °C and CO_2_ partial pressures greater than 3 × 10^–5^ MPa, lansfordite can form from a parent Mg­(HCO_3_)_2_ solution.^155^ Above 10 °C, nesquehonite becomes the main phase.^155^ Thermal analyses have also been extensively applied to characterize the phase transformations of nesquehonite, hydromagnesite, and magnesite versus the temperature change. Generally, nesquehonite’s dehydration occurs below 300–350 °C, and its decarbonation happens above 300–350 °C.^139,140,144,156–158^ Ren et al.^159^ reported that nesquehonite from a MgCl_2_–Na_2_CO_3_ system is thermally stable up to 104 °C, after which dehydration begins. Jauffret et al.^158^ found that nesquehonite’s dehydration occurs between 55 and 67 °C and 240–254 °C, with a mass loss corresponding to 2.5 mol of water. Decarbonation of nesquehonite, however, happened between 365 and 525 °C. For hydromagnesite, Ren et al.^159^ reported thermal stability up to 243 °C, where dehydration begins in a MgCl_2_–Na_2_CO_3_ system. Bhattacharjya et al.^160^ observed dehydration of hydromagnesite between 200 and 300 °C and decarbonation between 300 and 450 °C. Vágvölgyi et al.^144^ defined the primary thermal heating of hydromagnesite as dehydration and dehydroxylation steps, where decomposition of the OH group happens for the later one. The authors found that in a Mg­(NO_3_)_2_–HCO_3_^–^ system, hydromagnesite’s dehydration occurs at 25–181 °C, dehydroxylation at 181–274 °C, and decarbonation at 274–394 °C. Decarbonation of magnesium carbonate generally starts around 400–450 °C and above.^154,161,162^ At 700 °C, magnesite is usually completely decomposed to MgO.^162^ Variations in dehydration and decarbonation temperatures are attributed to experimental differences, including heating rate and decomposition atmosphere.^139,156,158^ In addition to affecting the decomposition of magnesium carbonates, the heating rate (°C/min) influences the quality of the precipitates. Guermech et al.^145^ showed that the grain size of both hydromagnesite and nesquehonite more than doubled when the heating rate was changed from 0.25 to 1 °C/min. This is an important consideration for scaling up to an industrial scale.

Solution pH is also a factor affecting the formation of different magnesium carbonate phases. A change in solution pH affects the balance between the HCO_3_^–^ and CO_3_²– ions, thereby influencing the solubility of the magnesium carbonate phase. A solution pH of 9 has been reported as optimal for the precipitation of highly pure hydromagnesite from a Mg-containing leachate obtained by processing serpentine with HNO_3_ and HCl acids.^97^ Zhang et al.^142^ studied the effect of pH on the transition temperature of Nesquehonite to hydromagnesite during a magnesium carbonate precipitation process at a temperature range of 45 to 95 °C. They found that at pH values below 9.5 and temperatures up to 65 °C, nesquehonite was the dominant carbonate phase. Nesquehonite remained the main product up to a maximum pH of 11.5 at a 45 °C system and up to a maximum pH of 10.5 at 65 °C, after which hydromagnesite became the major phase. Further increases in temperature and pH resulted in morphological changes in the carbonate product, from sheet-like to layer-like.^142^

Solution impurities can also influence the type of magnesium carbonate phase formation. Impurity ions with similar chemical properties to Mg, such as Ca, Na, and K, have the most impact as they typically compete with Mg during the carbonation process.^7^ In the presence of Ca ions, Mg can be trapped during carbonation, leading to the formation of complex magnesian calcite instead of hydromagnesite, thus affecting the final precipitate’s stability.^163^ However, Dong et al.^164^ found that the presence of K positively affects the formation of MgCO_3_ by increasing the hydration energy of Mg^2+^, shortening the presence of the hydromagnesite phase, and promoting the transformation into the anhydrous MgCO_3_ phase.^164^ Additionally, it was shown that a high presence of silica, about 6 mM, in serpentine-rich materials yields a higher amount of nesquehonite.^165^ All the factors discussed above influence the type and quality of the final magnesium carbonate formed and consequently the marketability of the product from the carbonation process. Magnesite is typically used in cosmetics, pharmaceuticals, food, and the rubber industry. It is also used as a flame retardant.^145^ Nesquehonite can be used for producing Reactive Magnesium Oxide Cement (RMC), which is an alternative to the more widely used Portland cement due to its less energy and CO_2_ emissions during production.^166^ The application of nesquehonite for making RMC is preferred over other carbonate phases because of its compact and interlocked needle-like structure.^166^ Lastly, hydromagnesite is mostly used industrially as a fire retardant.^97^

More attention should be given to the design of experiments and the respective operating conditions, considering the intended final carbonate product and its market demand.

#### 3.3.2 Process Economic and Environmental Considerations

The economic feasibility of ex situ CO_2_ mineralization of serpentine materials is crucial for large-scale implementation. While some carbonation technologies aim to generate revenue from valuable products, their large-scale application may be challenging. Utilizing abundant mining materials coupled with disposing of mineral carbonate products through a landfill or mine filling could improve process economics. Therefore, CO_2_ mineralization is expected to incur operational and capital expenses. The overall cost is influenced by various factors, including feedstock characteristics, process conditions, energy consumption, chemical reagents, and potential revenue streams. One of the primary cost drivers is feedstock preparation. The mineral feedstock, such as mine tailings, often requires pretreatment (e.g., grinding, heat activation) to enhance its reactivity, as confirmed by data analysis in Section 3 of this work. These pretreatment steps can significantly impact capital and operational expenditures, particularly when high energy inputs are required. The other key factors related to the reaction conditions include pressure, temperature, and solid-to-liquid ratio. High-pressure and high-temperature carbonation processes often not only accelerate reaction kinetics but also increase energy demands. Optimizing these parameters is essential to balance process efficiency and cost-effectiveness. Additionally, the selection and consumption of chemical reagents, such as acids for extraction and alkaline agents for carbonation, contribute to operating expenses. The use of recycled reagents or industrial waste streams can help mitigate these costs.

Tebbiche et al.^81^ conducted an energy analysis for direct mineral carbonation of thermally preactivated serpentine rocks at around 450 °C. This process aimed to capture 0.5 megatons of CO_2_ per year from a cement plant’s flue gas containing 30% CO_2_. They concluded that applying heat integration for solid preheat treatment reduced the process heat demand by 25%, resulting in an overall heat demand of 5.0 GJ per tonne of CO_2_ captured or 9.5 GJ per tonne of CO_2_ avoided.

In pH swing indirect CO_2_ carbonation, extraction chemicals are typically employed as extractive agents to enhance the dissolution of Mg^2+^ or Ca^2+^ prior to the carbonation stage. For the process to be economically viable, the regeneration of these extraction agents is essential.^3,97,132,171^ ^,^ The technique for regeneration is highly dependent on the nature of the extraction agent used. Extraction agents typically include inorganic acids, organic nonamino acids, amino acid-based agents, and ammonium-based salts. Among these, inorganic and organic acids are the most challenging to regenerate, as they necessitate additional steps such as distillation, evaporation, or electrolysis. Hydrochloric acid (HCl), nitric acid (HNO_3_), and sulfuric acid (H_2_SO_4_) are the widely applied inorganic acids for the CO_2_ mineralization route through the pH swing processes, where active metals are first extracted into the solution.

Teir et al.^52,97^ proposed a process scheme for producing hydromagnesite from serpentinite using HCl or HNO_3_, where excess acids are recovered and reused through an evaporation unit. They noted that while evaporating the excess extraction solvent requires significant heat, it is necessary to minimize additional chemical makeups. Due to the large demands for HCl or HNO_3_ (approximately 2.1 tonnes of HCl and 3.6 tonnes of HNO_3_ per tonne of CO_2_ sequestered), chemical recovery is essential. This can be achieved through electrolysis of salt byproducts, that is, NaCl or NaNO_3_, like commercially available chlor-alkali electrolyzer units. Additionally, about 2.4 tonnes of NaOH per tonne of CO_2_ sequestered are required. This results in higher costs for the makeup chemicals than the value of 1 tonne of CO_2_ carbon credit, regardless of the credit’s location. Their preliminary economic investigation raised questions about the feasibility of the process, as the chemicals used (HCl, HNO_3_, or NaOH) alone cost between 1300–1600 dollars/t-CO_2_ sequestered if not recovered.^97^ For the process including recovery steps, they estimated that the power consumed by the required equipment, specifically the electrolyzer and evaporator, would be approximately 11,800–15,700 MJ/t-CO_2_-sequestered and 1908 MJ/t-CO_2_-sequestered, respectively. For the electrolysis unit alone, this translates to average CO_2_ equivalent emissions of 2.7–3.6, 1.6–2.1, and 0.75–1.00 tonnes of CO_2_ per tonne of CO_2_ sequestered when using pulverized coal-fired, natural gas combined-cycle, and biomass power plants, respectively. When renewable energies are used, the emissions decrease significantly to 0.157–0.209, 0.078–0.105, and 0.039–0.052 tonnes of CO_2_ per tonne of CO_2_ sequestered for solar, hydro, and wind power plants, respectively.^168^ This highlights the critical importance of the energy source used in the recovery process, as it could potentially result in a larger carbon footprint than the amount of carbon being sequestered.

There are other works related to the recovery of HCl, where the HCl is recovered from a middle product, prior to the carbonation stage. For example, Goff and Lackner^169^ proposed recovering hydrochloric acid from a hydrated magnesium chloride solution using multistage evaporation. During this process, the MgCl_2_ containing solution is hydrolyzed at 200–250 °C to form Mg­(OH)Cl and HCl. Upon further cooling, Mg­(OH)Cl dissociates into magnesium chloride and magnesium hydroxide.

Other popular extraction agents used for the extraction of metals include acetic, citric, lactic, formic, carbonic, and oxalic acids. Although there are no studies available on the recovery of such organic acids in the mineralization processes of serpentine-based feedstocks, some researchers have proposed processes for the recovery of these acids for other applications. Kakizawa et al.^170^ investigated the indirect CO_2_ mineralization of natural calcium silicate using acetic acid. They proposed that acetic acid can be recovered during the carbonation stage, where calcium carbonate is precipitated out by introducing CO_2_ gas, resulting in the reproduction of acetic acid. Teir et al.^171^ proposed a similar acid recovery process for carbonating of iron and steel slags containing magnesium silicates. Eloneva et al.^172^ investigated accelerating calcium carbonate formation from blast furnace slag-derived calcium acetate by adding sodium hydroxide during the carbonation stage. They proposed recovering excess acetic acid at 120 °C through evaporation and condensation. However, they noted that the process would likely produce more CO_2_ than it sequesters.

Regarding the application of ammonium-based chemicals, Wang and Maroto-Valer^99,117^ proposed a process for extracting magnesium from naturally sourced serpentine using ammonium bisulfate, which is considered one of the most efficient ammonium-based solvents. This process is further coupled with the carbonation of the magnesium solution derived using ammonium bicarbonate (NH_4_HCO_3_) and ammonia (NH_3_). In the proposed system, the NH_4_HSO_4_ extraction agent was regenerated from the (NH_4_)_2_SO_4_ solution (from the carbonation stage) through two stages: evaporation at ∼60 °C, followed by thermal decomposition at ∼300–330 °C. As noted by the authors, the process requires approximately 8.48 tonnes of NH_4_HSO_4_, 7.48 tonnes of NH_4_HCO_3_, and 0.8 tonnes of NH_3_ to sequester 1 tonne of CO_2_ without a chemical recovery step. The consumption of NH_4_HSO_4_ and NH_3_ decreased to 0.12 and 0.04 tonnes, respectively, when the chemical recovery stage was included. The authors claimed that the cost of chemicals in their process is significantly lower than the cost associated with using inorganic acids, such as HCl. Sanna et al.^173^ investigated a methanol-based liquid–liquid extraction technique to reduce the energy penalty of producing (NH_4_)_2_SO_4_. They achieved over 90% separation of ammonium sulfate from water at 25 °C for 10 min, 1 bar, and a 200 g/L S/L ratio using 70% methanol. The authors noted that the extraction process required 2908 kWh of heating energy, while water evaporation needed 4465 kWh to sequester 1 tonne of CO_2_, reducing energy consumption by 35%. With all that being said, more analysis on the application of serpentine-based materials for CO_2_ mineralization is needed to make conclusive comparisons with different chemical agents and their impact on the environmental and economic aspects of the processes

Few economic analysis studies have thoroughly assessed the viability of carbonation, considering both operational costs (Opex) and capital costs (Capex). Narahisetti et al.^167^ conducted an extensive economic study on direct aqueous carbonation processes using minerals like wollastonite, olivine, and serpentine, considering operating conditions and various chemicals used. They emphasized selecting abundant, low-cost mineral feedstock and strategies to minimize electricity consumption, such as reducing the operational pressure and temperature requirements. The authors concluded that wollastonite is the most viable mineral for CO_2_ carbonation. Their study found that, depending on the minerals and scenarios analyzed, the Opex ranges from 172 to 245 dollars per tonne of CO_2_ avoided with natural gas as the power source, and the production cost is between 343 and 504 dollars per tonne avoided if both Opex and Capex are included. Their estimated Opex data was comparable with literature data, such as the study by Hitch and Dipple,^174^ which reported an operating cost of 28 dollars to 238 dollars per tonne of CO_2_ sequestrated for a high-temperature and pressure industrial mineral carbonation facility integrated into a nickel mine site in Northern BC, Canada. This site contained abundant magnesium silicate minerals in its waste rock. The cost associated with Capex in their study was about 90 dollars per tonne of CO_2_ avoided. Pasquier et al.^175^ conducted an economic study on direct aqueous carbonation of serpentine-based mining residues using raw flue gas with 18% CO_2_. They conducted a detailed analysis by examining the energy sources for heat activation and precipitation independently of the electricity sources used for the other units of the process. This approach allowed them to explore various options, including biomass, natural gas, hydro, and coal, for each specific need. By doing so, they aimed to identify the most efficient and sustainable energy sources for each part of the process. The authors found that hydrogenerated electricity resulted in fewer GHG emissions compared with natural gas or coal. Using biomass for heat also significantly improved the CO_2_ balance. Their results showed that 7.8 GJ/t of CO_2_ is required to sequester 234 kg of CO_2_ per tonne of rocks. Depending on the source of electricity and heat, the storage costs ranged from 146 to 1049 dollars per tonne of CO_2_ avoided. When hydroelectricity is used as the electricity source, the storage cost remains below $250 per tonne of CO_2_ avoided. Under optimal heat and power scenarios (hydro and biomass for electrical and heat demands, respectively), the storage cost was estimated at 146 dollars per tonne of CO_2_ avoided.

Environmental life cycle assessment is also an important study required for mineralization processes as part of their integration into Carbon Capture and Storage (CCS) technologies. Giannoulakis et al.^176^ conducted a study on the environmental effects and life cycle analysis of integrating mineral carbonation into fossil-fueled (natural gas and coal) power generation plants. They investigated the impact on six environmental burdens: human toxicity, particulate matter formation, terrestrial acidification, marine eutrophication, urban land occupation, and photochemical oxidant formation. The study analyzed three types of waste for mineral carbonation: wollastonite, serpentine, and olivine for direct aqueous-based carbonation and serpentine for direct gas–solid carbonation. Implementing mineral carbonation in fossil-fueled power generation reduced life cycle GHG emissions by 15–64% compared to electricity production without CCS. However, the energy and solvent requirements of the carbonation processes significantly contribute to GHG emissions. Among the natural minerals, wollastonite was the most favorable, both environmentally and economically. Olivine was better than serpentine environmentally, but serpentine offered a lower power generation cost. Nduagu et al.^177^ studied CO_2_ sequestration from a coal power plant using magnesium silicate rock. CO_2_ carbonation occurred through gas–solid reactions at high temperatures or direct aqueous-based processes. Exergy calculations showed that 3.4 GJ is needed to mineralize 1 tonne of CO_2_ using direct aqueous-based CO_2_ carbonation with a heat recovery configuration. The life cycle assessment indicated approximately 683 kg of CO_2e_ emissions per tonne of CO_2_ sequestered.

## 4 Conclusion

This study provides a comprehensive analysis of CO_2_ mineralization in magnesium-based mine wastes, utilizing an extensive data library compiled from the existing literature. Both direct and indirect mineralization approaches, including extraction and carbonation, were evaluated to understand the interactions of key process parameters. The combination of artificial neural networks (ANN) and a 3^k^ full-factorial design enabled a detailed investigation of the nonlinear relationships and the statistical significance of influencing factors. The analysis revealed the following key insights:

•For the extraction process, feedstock material pretreatment, extraction agent, temperature, and particle size are the most important factors for optimizing the extraction process crucial for enhancing extraction efficiency and play a vital role in the efficiency of the process.

•For direct CO_2_ carbonation, the findings guide optimization by prioritizing pretreatment, increasing the CO_2_ concentration, and particle size reduction, with solution chemistry (carbonation assistance agent, reaction pH, and reaction temperature) as a secondary consideration, to enhance CO_2_ sequestration from solid waste feedstocks.

•For the indirect carbonation approach, carbonation assisting agents and solution pH are dominant, reflecting the primacy of solution chemistry in aqueous carbonation. pH controls CO_2_ speciation (e.g., CO_3_^2–^ availability), while agents like NaOH enhance CO_2_ dissolution and ion reactivity. The reaction conditions (CO_2_ pressure and temperature) and the concentration of Mg ions extracted into the solution play a moderate to minimal role, in contrast to the direct carbonation stage, where physical factors (pretreatment, particle size, and CO_2_ concentration) are emphasized. These differences reflect the aqueous nature of indirect carbonation versus the solid-phase dominance in direct carbonation, guiding tailored optimization strategies for enhanced CO_2_ sequestration from solid wastes.

These findings offer valuable insights into process optimization, guiding future experimental research and industrial-scale implementation of CO_2_ mineralization for sustainable waste management and carbon capture. By understanding the most influential factors, researchers and industry professionals can better design and optimize processes to achieve higher efficiency and sustainability.

## Supplementary Material



## Notes

S1. Details of the developed ANN model. Figure S1. Flowchart of the ANN analysis developed in this work. Figure S2. Contour plots of interaction between critical affecting factors on magnesium extraction efficiency, created with 80% data used for modeling; (A) temperature (Factor 4) vs particle size (Factor 2), (B) pretreatment (Factor 6) vs particle size (Factor 2), (C) extraction agent (Factor 6) vs particle size (Factor 2), (D) pretreatment (Factor 6) vs temperature (Factor 4), (E) extraction agent (Factor 7) vs temperature (Factor 4), (F) extraction agent (Factor 7) vs pretreatment (Factor 6). Figure S3. Contour plots of interaction between critical factors affecting direct CO_2_ mineralization capacity, created with 80% data used for modeling; (A) particle size (Factor 2) vs CO_2_ concentration (Factor 7), (B) particle size (Factor 2) vs carbonation assisting agent (Factor 9), (C) CO_2_ concentration (Factor 7) vs pretreatment (Factor 8), (D) CO_2_ concentration (Factor 7) vs carbonation assisting agent (Factor 9). Figure S4. Contour plots of interaction between critical factors affecting indirect CO_2_ mineralization capacity, created with 80% data used for modeling; (A) extracted Mg concentration (Factor 1) vs solution pH (Factor 3), (B) extracted Mg concentration (Factor 1) vs carbonation assisting agent (Factor 5), (C) solution pH (Factor 3) vs CO_2_ pressure (Factor 4), (D) solution pH (Factor 3) vs carbonation assisting agent (Factor 5), (E) CO_2_ pressure (Factor 4) vs carbonation assisting agent (Factor 5). Figure S5. Effect of combined factors on the extraction efficiency with 100% data used for modeling (noncropped version). Figure S6. Effect of combined factors on the extraction efficiency with 80% data used for modeling (noncropped version). Figure S7. Effect of combined factors on CO_2_ sequestration capacity with 100% data used for modeling. (A) direct carbonation (B) indirect carbonation (noncropped version). Figure S8. Effect of combined factors on CO_2_ sequestration capacity with 80% data used for modeling. (A) direct carbonation (B) indirect carbonation (noncropped version). Table S1. ANN network architecture summary. Table S2. Article library used for detailed analysis. Table S3. Results for ANOVA Statistical Analysis with 100% of extraction efficiency data. Table S4. Results for ANOVA statistical analysis with 80% of extraction efficiency data. Table S5. Results for ANOVA statistical analysis with 100% of CO_2_ sequestration capacity data-direct carbonation. Table S6. Results for ANOVA statistical analysis with 80% of CO_2_ sequestration capacity data-direct carbonation. Table S7. Results for ANOVA statistical analysis for 100% of CO_2_ sequestration capacity data-indirect carbonation. Table S8. Results for ANOVA statistical analysis with 80% of CO_2_sequestration capacity data- indirect carbonation (PDF)
