# Supplementary material for: Application of Artificial Neural Networks and Factorial Design Analysis for Predicting the Interaction of Influencing Process Parameters in CO2 Mineralization of Magnesium-Rich Mining Materials
Source: ACS Omega. 2025 Oct 9;10(41):48614–41. doi: 10.1021/acsomega.5c06358 (PMC12547545; doi:10.1021/acsomega.5c06358)
Supplement: Supplementary file 1 [file ao5c06358_si_001.pdf]

# Supporting information

# Application of artificial neural networks and factorial design analysis for predicting interaction of influencing process parameters in $CO_2$ mineralization of magnesium-rich mining materials

Iris Samputu, Hamid Radfarnia*, Kourosh Zanganeh

Natural Resources Canada, CanmetENERGY, 1 Hannel Drive, Ottawa, ON K1A 1M1, Canada

* Corresponding author

E-mail: hamid.radfarnia@nrcan-rncan.gc.ca

**S1. Details of the developed ANN model**

An ANN model generally consists of three main layers; input, output, and one or more hidden layers, as shown in Figure S1[1]. This work specifically examines the application of a multi-layer feed- forward backpropagation network. Multi-layer feed-forward networks have been predominantly applied in modelling non-linear processes for the optimization of multiple parameters[2,3]. Each layer in the neural network consists of neurons characterized by specific weights and biases. These weights and biases are updated through network training, using backpropagation method. This process generates a model that is validated and assessed for optimization. With the backpropagation training algorithm, the neural network learns and adjusts the comparative weights and biases for each node in each layer, aiming to minimize the error of the predicted outputs. Eq.(1) describes the mathematical algorithm applied to neurons in the developed ANN model.

$$N_{in} = \sum_{i=1}^{n}(W_i X_i) + b \qquad (1)$$

where, $N_{in}$, $W_i$, $X_i$, and $b$ are the calculated input for each neuron in one layer, the weight, the corresponding input and the associated bias, respectively. The associated bias is used to decrease variance by adjusting the predicted output when fed into the activated function. Additionally, (*i)* and (*n*) represent the index and the number of inputs (factors/parameters), respectively. Using the calculated weights and biases, the calculated input ($N_{in}$) is then processed in the output layer with an activation function, resulting in the first output as shown in Eq.(2).

$$N_{out} = f(N_{in}) \qquad (2)$$

where $N_{out}$ is the resulting output of each neuron and $f(N_{in})$ is the activation function. The activation function introduces non-linearity in the input-output data relationship. Among the

activation functions, log and tan sigmoid functions are most frequently used because they reduce the computational burden during training[4,5]. In this work, a log sigmoid function was used as the activation, as it adequately fit the data. The output variable generated has a value between 0 and 1 and is then fed into a linear transfer function to generate a continuous value as the final output. Multiple hidden layers were also used, adding multiple levels of complexity, as each layer contains different neurons with individual weights and biases[2,3].

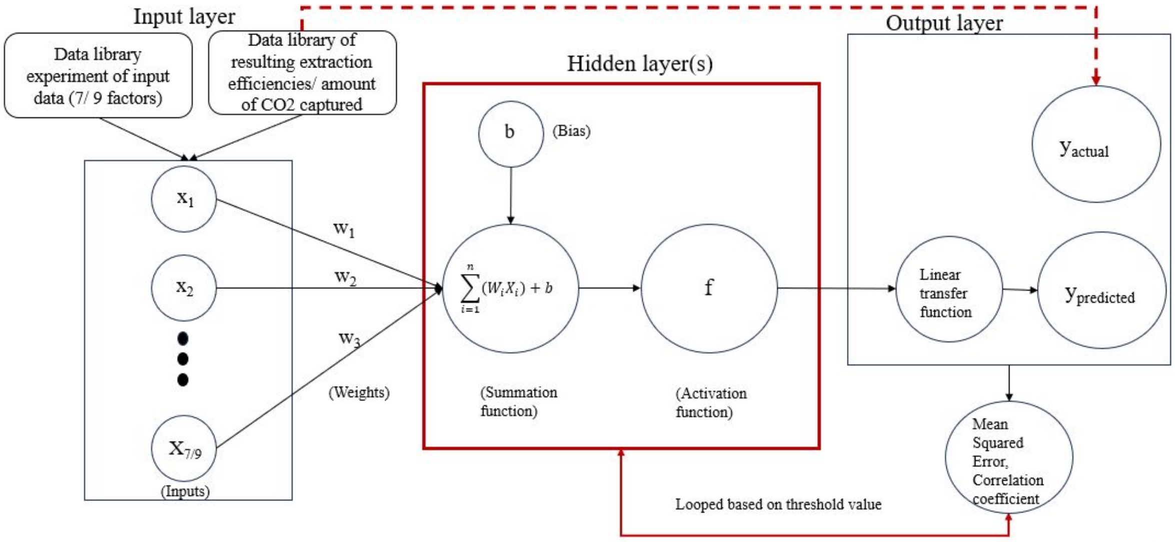


Figure S1. Flowchart of the ANN analysis developed in this work.

The network architecture used in this work had the following features:

Table S1. ANN network architecture summary

| Architecture | Features |
|---|---|
| Input layer | Variable number of neurons depending on the specific model (7 neurons for extraction, 9 neurons for direct carbonation, 5 neurons for indirect carbonation) |
| Hidden layers | Multiple configurations tested (10, 20, 50, and 100 neurons), with optimal performance achieved using networks with 50 hidden |

| | |
|---|---|
| | neurons for direct and 100 for indirect carbonation and 100 for extraction |
| Output layer | Single neuron for prediction of extraction efficiency or $CO_2$ sequestration capacity |
| Network topology | Fully connected multilayer perceptron (MLP) architecture |
| Activation function- Hidden Layers | Hyperbolic tangent sigmoid (tansig) function |
| Activation function- Output Layers | Linear activation function for regression output |
| Training Algorithm | Levenberg-Marquardt backpropagation algorithm was selected as the optimal training method after comparing seven different algorithms including BFGS quasi-Newton, scaled conjugate gradient, and Fletcher-Powell conjugate gradient. The Levenberg-Marquardt algorithm demonstrated superior performance. |
| Performance analysis | Lowest mean squared error, Highest correlation coefficient |

References


1 Uddameri, V. Using Statistical and Artificial Neural Network Models to Forecast Potentiometric Levels at a Deep Well in South Texas. *Environ. Geol.* **2007**, *51* (6), 885–895. https://doi.org/10.1007/s00254-006-0452-5.

2 Paturi, U. M. R.; Cheruku, S.; Reddy, N. S. The Role of Artificial Neural Networks in Prediction of Mechanical and Tribological Properties of Composites—A Comprehensive Review. *Arch. Comput. Methods Eng.* **2022**, *29* (5), 3109–3149. https://doi.org/10.1007/s11831-021-09691-7.

3 Multilayer Shallow Neural Network Architecture - MATLAB & Simulink. https://www.mathworks.com/help/deeplearning/ug/multilayer-neural-network-architecture.html (accessed June 2025).

4 Fausett, L. V. *Fundamentals of Neural Networks: Architectures, Algorithms and Applications*; Pearson Education, 2006.

5 Pratiwi, H.; Windarto, A. P.; Susliansyah, S.; Aria, R. R.; Susilowati, S.; Rahayu, L. K.; Fitriani,Y.; Merdekawati, A.; Rahadjeng, I. R. Sigmoid Activation Function in Selecting the Best Model of Artificial Neural Networks. *J. Phys. Conf. Ser.* **2020**, *1471* (1), 012010. https://doi.org/10.1088/1742-6596/1471/1/012010.

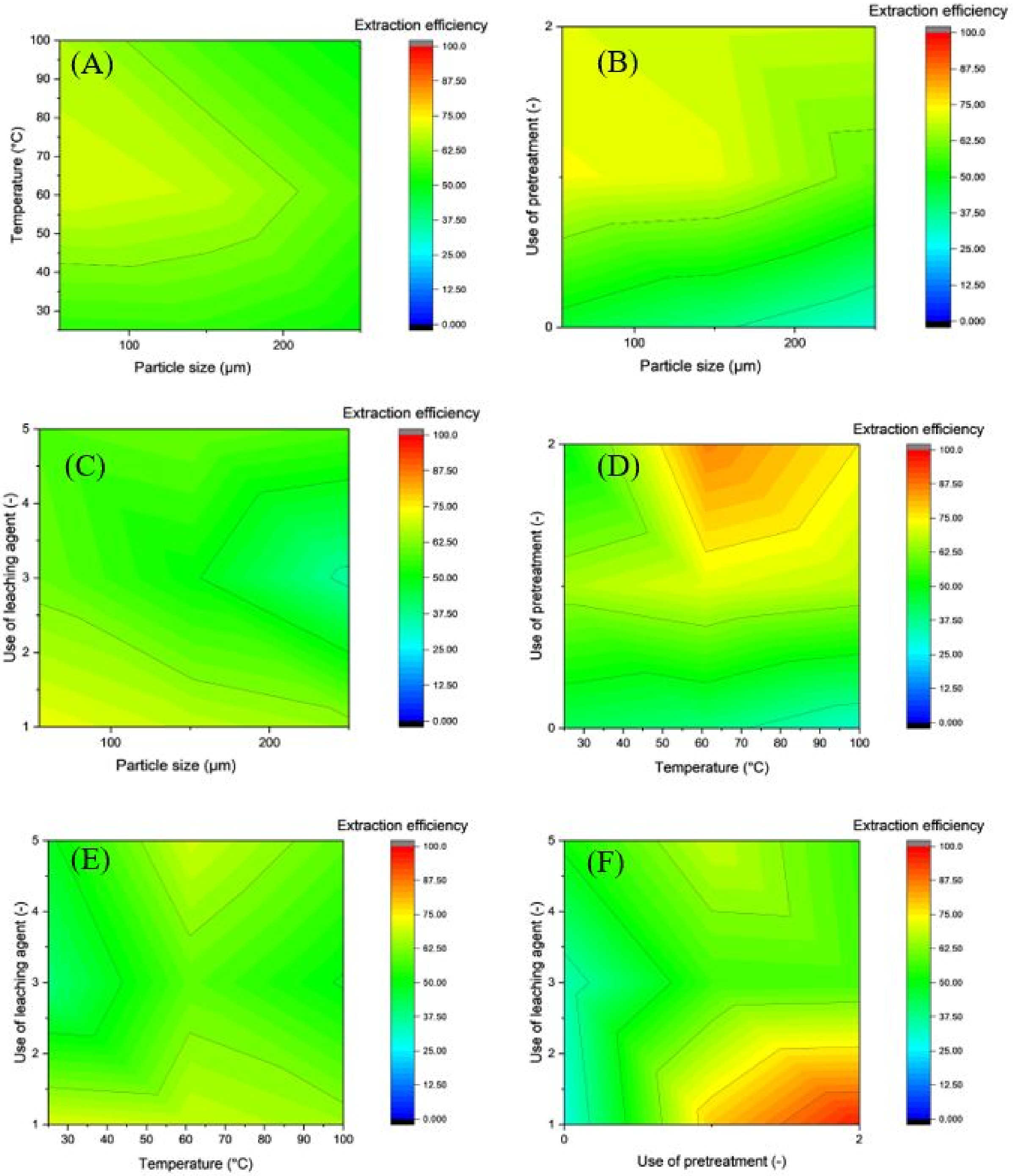


Figure S2. Contour plots of interaction between critical affecting factors on magnesium extraction efficiency, created with 80% data used for modeling; (A) temperature (Factor 4) vs. particle size (Factor 2), (B) pretreatment(Factor 6) vs. particle size (Factor 2), (C) extraction agent(Factor 6) vs. particle size (Factor 2), (D) pretreatment(Factor 6) vs. temperature(Factor 4), (E) extraction agent (Factor 7) vs. temperature(Factor 4), (F) extraction agent (Factor 7) vs. pretreatment (Factor 6). For the extraction agent (1- inorganic acids, 2-strong acidic salts of ammonia and sodium; ($NH_4$HSO4 and NaHSO4), 3- organic acids and weak acidic salts of ammonia ($NH_4H_2SO_4$, acetic, formic acid), 4- other reagents (carbonic acid, chelating agents), 5- water alone).

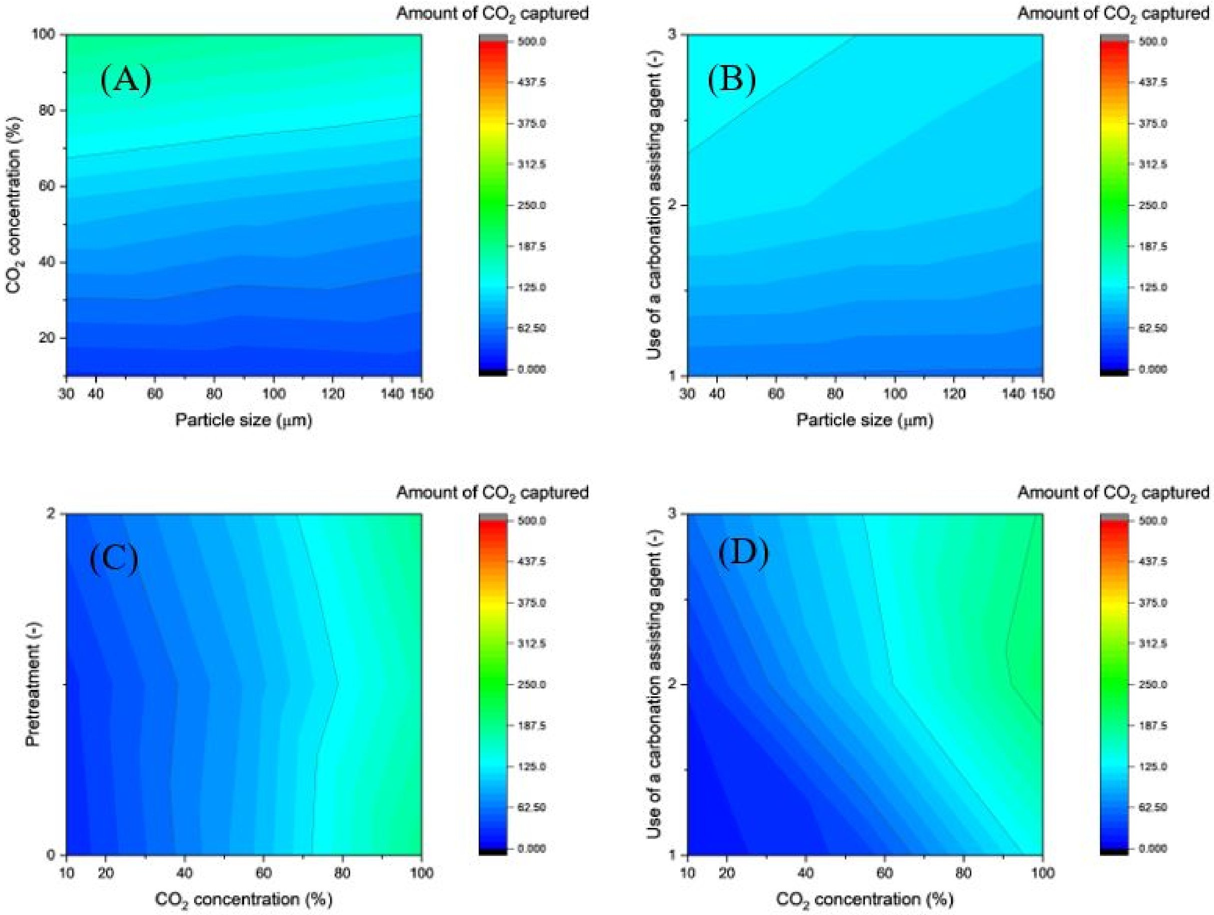


Figure S3. Contour plots of interaction between critical factors affecting direct $CO_2$ mineralization capacity, created with 80% data used for modeling; (A) particle size (Factor 2) vs. $CO_2$ concentration (Factor 7), (B) particle size (Factor 2) vs. carbonation assistant agent (Factor 9), (C) $CO_2$ concentration (Factor 7) vs. pretreatment (Factor 8), (D) $CO_2$ concentration (Factor 7) vs. carbonation assisting agent (Factor 9). For the carbonation assisting agent (1- carbon source/buffer added ($NaHCO_3$+NaCl) or (EDTA), 2-$NaHCO_3$, 3- water alone).

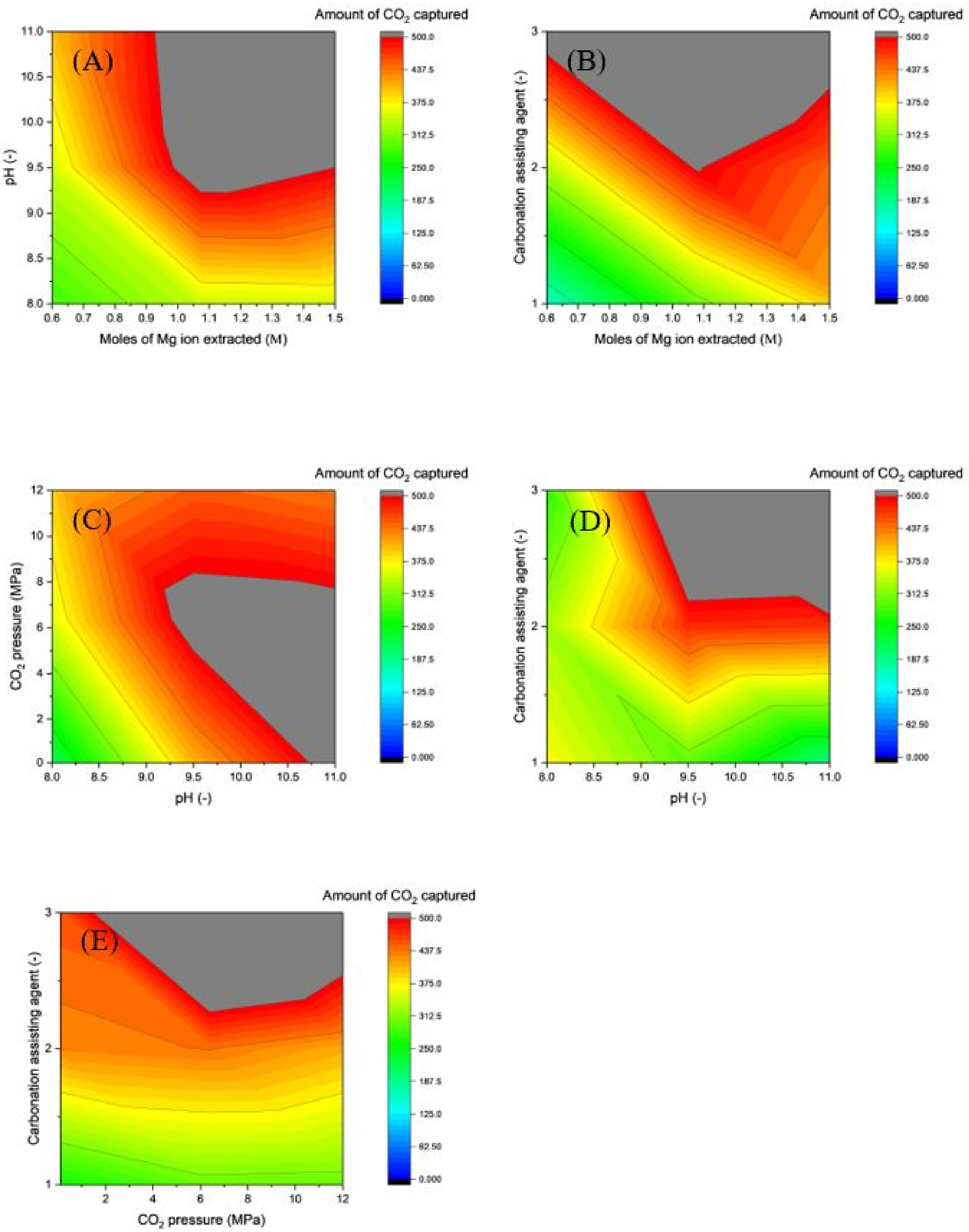


Figure S4. Contour plots of interaction between critical factors affecting indirect $CO_2$ mineralization capacity, created with 80% data used for modeling; (A) extracted Mg concentration (Factor 1) vs. solution pH (Factor 3), (B) extracted Mg concentration (Factor 1) vs. carbonation assistant agent (Factor 5), (C) solution pH (Factor 3) vs. $CO_2$ pressure (Factor 4), (D) solution pH

(Factor 3) vs. carbonation assisting agent (Factor 5), (E) $CO_2$ pressure (Factor 4) vs. carbonation assisting agent (Factor 5). For the carbonation assisting agent (1-strong base (NaOH), 2-Buffer/chelating agent and carbon source or amine agent (NaHCO3, with NaCl, Tiron, triethylamine etc.), 3- no additive (water)).

Table S2. Article library used for detailed analysis

| Article Index | Used for extraction study | Used for carbonation study | Reference |
|---|---|---|---|
| 1 | | ✓ | I. M. Power, G. M. Dipple, P. M. D. Bradshaw, and A. L. Harrison, "Prospects for $CO_2$ mineralization and enhanced weathering of ultramafic mine tailings from the Baptiste nickel deposit in British Columbia, Canada," International Journal of Greenhouse Gas Control, vol. 94, p. 102895, Mar. 2020, doi: 10.1016/j.ijggc.2019.102895. |
| 2 | | ✓ | C. M. Woodall, X. Lu, G. Dipple, and J. Wilcox, "Carbon Mineralization with North American PGM Mine Tailings—Characterization and Reactivity Analysis," Minerals, vol. 11, no. 8, p. 844, Aug. 2021, doi: 10.3390/min11080844. |
| 3 | | ✓ | G. P. Assima, F. Larachi, J. Molson, and G. Beaudoin, "Emulation of ambient carbon dioxide diffusion and carbonation within nickel mining residues," Minerals Engineering, vol. 59, pp. 39–44, May 2014, doi: 10.1016/j.mineng.2013.09.002. |
| 4 | ✓ | | R. C. P. Eusebio, B. J. B. Razote, H. J. T. Del Pilar, R. D. Alorro, A. B. Beltran, and A. H. Orbecido, "Evaluation of the leaching characteristics of low-grade nickel laterite waste rock for indirect carbon sequestration application," Geosystem Engineering, vol. 23, no. 4, pp. 205–215, Jul. 2020, doi:10.1080/12269328.2020.1745694. |
| 5a* | | ✓ | G. P. Assima, F. Larachi, J. Molson, and G. Beaudoin, "Comparative study of five Québec ultramafic mining residues for use in direct ambient carbon dioxide mineral sequestration," Chemical Engineering Journal, vol. 245, pp. 56–64, Jun. 2014, doi: 10.1016/j.cej.2014.02.010. |
| 5b* | | ✓ | G. P. Assima, F. Larachi, J. Molson, and G. Beaudoin, "Comparative study of five Québec ultramafic mining residues for use in direct ambient carbon dioxide mineral sequestration," Chemical Engineering Journal, vol. 245, pp. 56–64, Jun. 2014, doi: 10.1016/j.cej.2014.02.010. |
| 6 | ✓ | | S. Teir, S. Eloneva, C.-J. Fogelholm, and R. Zevenhoven, "Fixation of carbon dioxide by producing hydromagnesite from serpentinite," Applied Energy, vol. 86, no. 2, pp. 214–218, Feb. 2009, doi:10.1016/j.apenergy.2008.03.013. |
| 7 | | ✓ | J. Pronost et al., "Carbon Sequestration Kinetic and Storage Capacity of Ultramafic Mining Waste," Environ. Sci. Technol., vol. 45, no. 21, pp. 9413–9420, Nov. 2011, doi:10.1021/es203063a. |
| 8 | ✓ | ✓ | S. Teir, R. Kuusik, C.-J. Fogelholm, and R. Zevenhoven, "Production of magnesium carbonates from serpentinite for long-term storage of CO2," International Journal of Mineral Processing, vol. 85, no. 1–3, pp.1–15, Dec. 2007, doi: 10.1016/j.minpro.2007.08.007. |
| 9a* | | ✓ | F. Wang and D. Dreisinger, "An integrated process of CO2 mineralization and selective nickel and cobalt recovery from olivine and laterites," Chemical Engineering Journal, vol. 451, p. 139002, Jan. 2023, doi:10.1016/j.cej.2022.139002. |

| | | | |
|---|---|---|---|
| 10 | | ✓ | M. Werner, S. B. Hariharan, A. V. Bortolan, D. Zingaretti, R. Baciocchi, and M. Mazzotti, “Carbonation of Activated Serpentine for Direct Flue Gas Mineralization,” Energy Procedia, vol. 37, pp. 5929–5937, 2013, doi:10.1016/j.egypro.2013.06.519. |
| 11 | | ✓ | W.K. O’Connor G.E. Rush D.C. Dahlin S.P. Reidel V.G. Johnson Geological Sequestration of CO2 in the Columbia River Basalt Group 2003 Clearwater, Florida. |
| 12 | | ✓ | F. Bodénan et al., “Ex situ mineral carbonation for CO2 mitigation: Evaluation of mining waste resources, aqueous carbonation processability and life cycle assessment (Carmex project),” Minerals Engineering, vol. 59, pp. 52–63, May 2014, doi: 10.1016/j.mineng.2014.01.011. |
| 13 | ✓ | | F. Wang, D. Dreisinger, and Y. Xiao, “Pre-treatment through reductive calcination for CO2 mineralization and selective battery metal extraction from laterites,” Separation and Purification Technology, vol. 340, p. 126818, Jul. 2024, doi: 10.1016/j.seppur.2024.126818. |
| 14 | ✓ | ✓ | I. Tebbiche, L.-C. Pasquier, G. Mercier, J.-F. Blais, and S. Kentish, “Thermally activated serpentine leaching under flue gas conditions in a bubble column reactor operated at ambient pressure and temperature,” Hydrometallurgy, vol. 195, p. 105391, Aug. 2020, doi: 10.1016/j.hydromet.2020.105391. |
| 15 | ✓ | | Park, A.H.A., Jadhav, R. and Fan, L.S. (2003) 'CO2 mineral sequestration: chemically enhanced aqueous carbonation of serpentine', The Canadian Journal of Chemical Engineering, 81(3-4), pp. 885-890. |
| 16 | ✓ | | Park, A.-H.A. and Fan, L.-S. (2004) 'CO2 mineral sequestration: physically activated dissolution of serpentine and pH swing process', Chemical Engineering Science, 59(22-23), pp. 5241-5247. |
| 17 | ✓ | | Sanna, A., Wang, X., Lacinska, A., Styles, M., Paulson, T. and Maroto-Valer, M.M. (2013) 'Enhancing Mg extraction from lizardite-rich serpentine for CO2 mineral sequestration', Minerals Engineering, 49, pp. 135-144. |
| 18 | ✓ | ✓ | Steel, K.M., Alizadehhesari, K., Balucan, R.D. and Bašić, B. (2013) 'Conversion of CO2 into mineral carbonates using a regenerable buffer to control solution pH' |
| 19 | ✓ | ✓ | Hemmati, A., Shayegan, J., Bu, J., Yeo, T.Y. and Sharratt, P. (2014) 'Process optimization for mineral carbonation in aqueous phase', International Journal of Mineral Processing, 130, pp. 20-27 |
| 20 | ✓ | | Arce Ferrufino, G.L.A., Okamoto, S., Dos Santos, J.C., de Carvalho, J.A., Avila, I., Romero Luna, C.M. and Gomes Soares Neto, T. (2018) 'CO2 sequestration by pHswing mineral carbonation based on HCl/NH4OH system using iron-rich lizardite 1T', Journal of CO2 Utilization, 24, pp. 164-173. |
| 21 | ✓ | ✓ | E. R. Bobicki, Q. Liu, and Z. Xu, “Mineral carbon storage in pre-treated ultramafic ores,” Minerals Engineering, vol. 70, pp. 43–54, Jan. 2015, doi: 10.1016/j.mineng.2014.08.009. |

| | | | |
|---|---|---|---|
| 22 | ✓ | ✓ | L.-C. Pasquier, G. Mercier, J.-F. Blais, E. Cecchi, and S. Kentish, "Reaction Mechanism for the Aqueous-Phase Mineral Carbonation of Heat-Activated Serpentine at Low Temperatures and Pressures in Flue Gas Conditions," Environ. Sci. Technol., vol. 48, no. 9, pp. 5163–5170, May 2014, doi:10.1021/es405449v. |
| 23 | ✓ | | G. L. A. F. Arce, T. G. Soares Neto, I. Ávila, C. M. R. Luna, and J. A. Carvalho,"Leaching optimization of mining wastes with lizardite and brucite contents for use in indirect mineral carbonation through the pH swing method," Journal of Cleaner Production, vol. 141, pp. 1324–1336, Jan. 2017, doi:10.1016/j.jclepro.2016.09.204. |
| 24 | ✓ | | Rim, G.; Marchese, A. K.; Stallworth, P.; Greenbaum, S. G.; Park, A.-H. A. 29Si Solid State MAS NMR Study on Leaching Behaviors and Chemical Stability of Different Mg-Silicate Structures for CO2 Sequestration. Chemical Engineering Journal 2020, 396, 125204. https://doi.org/10.1016/j.cej.2020.125204. |
| 25 | ✓ | | Wang, X.; Maroto-Valer, M. M. Dissolution of Serpentine Using Recyclable Ammonium Salts for CO2 Mineral Carbonation. Fuel 2011, 90 (3), 1229–1237. https://doi.org/10.1016/j.fuel.2010.10.040. |
| 26 | ✓ | | Teir, S.; Revitzer, H.; Eloneva, S.; Fogelholm, C.-J.; Zevenhoven, R. Dissolution of Natural Serpentinite in Mineral and Organic Acids. International Journal of Mineral Processing 2007, 83 (1), 36–46. https://doi.org/10.1016/j.minpro.2007.04.001. |
| 27 | ✓ | | Razote, B. J. B.; Maranan, M. K. M.; Eusebio, R. C. P.; Alorro, R. D.; Beltran, A. B.; Orbecido, A. H. Determination of the Carbon Dioxide Sequestration Potential of a Nickel Mine Mixed Dump through Leaching Tests. Energies 2019, 12 (15), 2877. https://doi.org/10.3390/en12152877. |
| 28 | ✓ | ✓ | Sanna, A.; Steel, L.; Maroto-Valer, M. M. Carbon Dioxide Sequestration Using NaHSO4 and NaOH: A Dissolution and Carbonation Optimisation Study. Journal of Environmental Management 2017, 189, 84–97. https://doi.org/10.1016/j.jenvman.2016.12.029. |
| 29 | ✓ | | Sanna, A.; Maroto-Valer, M. M. CO2 Sequestration Using a Novel Na-Salts pH Swing Mineral Carbonation Process. Energy Procedia 2014, 63, 5897–5903. https://doi.org/10.1016/j.egypro.2014.11.624. |
| 30 | ✓ | | Gao, W.; Wen, J.; Li, Z. Dissolution Kinetics of Magnesium from Calcined Serpentine in NH4Cl Solution. Ind. Eng. Chem. Res. 2014, 53 (19), 7947–7955. https://doi.org/10.1021/ie4043533. |
| 31 | ✓ | ✓ | Galina, N.R.; Arce, G.L.A.F.; Maroto-Valer, M.; Ávila, I. Experimental Study on Mineral Dissolution and Carbonation Efficiency Applied to pH-Swing Mineral Carbonation for Improved CO2 Sequestration. Energies 2023, 16, 2449. |
| 32 | ✓ | | Farhang, F.; Rayson, M.; Brent, G.; Hodgins, T.; Stockenhuber, M.; Kennedy, E. Insights into the Dissolution Kinetics of Thermally Activated Serpentine for CO2 Sequestration. Chemical Engineering Journal 2017, 330, 1174–1186. https://doi.org/10.1016/j.cej.2017.08.073. |

| | | | |
|---|---|---|---|
| 33 | ✓ | ✓ | Sanna, A.; Lacinska, A.; Styles, M.; Maroto-Valer, M. M. Silicate Rock Dissolution by Ammonium Bisulphate for pH Swing Mineral CO2 Sequestration. Fuel Processing Technology 2014, 120, 128–135. https://doi.org/10.1016/j.fuproc.2013.12.012. |
| 34 | | ✓ | Wang, X.; Maroto-Valer, M. M. Integration of CO2 Capture and Mineral Carbonation by Using Recyclable Ammonium Salts. ChemSusChem 2011, 4 (9), 1291–1300. https://doi.org/10.1002/cssc.201000441. |
| 35 | ✓ | | Lu, X.; Dipple, G. M.; Pawlik, M. A Surface Charge Approach to Characterize Serpentine Reactivity for Carbon Mineralization. Applied Clay Science 2025, 270, 107777. https://doi.org/10.1016/j.clay.2025.107777. |
| 36 | | ✓ | Katre, S.; Ochonma, P.; Mamidala, A.; Sahu, S.; Nair, A. M.; Ravi, K.; Gadikota, G. Organic Ligands and CO2 Unlock the Potential for Energy Relevant Metals Recovery and Carbon Mineralization from Mafic Rocks. Sci Rep 2025, 15 (1), 10882. https://doi.org/10.1038/s41598-025-94153-4. |
| 37 | | ✓ | Campione, M.; Corti, M.; D'Alessio, D.; Capitani, G.; Lucotti, A.; Yivlialin, R.; Tommasini, M.; Bussetti, G.; Malaspina, N. Microwave-Driven Carbonation of Brucite. Journal of CO2 Utilization 2024, 80, 102700. https://doi.org/10.1016/j.jcou.2024.102700. |
| 38 | ✓ | | Zhao, D.; Sun, H.; Peng, T.; Zeng, L.; Wu, M. The Acid-Leaching Process and Structural Changes of Lizardite, Chlorite and Talc in Sulfuric Acid Medium. Clay Minerals 2024, 59 (4), 298–309. https://doi.org/10.1180/clm.2024.19. |
| 39 | | ✓ | Abu Fara, A.; Rayson, M. R.; Brent, G. F.; Oliver, T. K.; Stockenhuber, M.; Kennedy, E. M. Effect of NaHCO3 on the Magnesite Yield in Direct Aqueous Carbonation of Thermally-Activated Lizardite. Ind. Eng. Chem. Res. 2024, 63 (3), 1314–1320. https://doi.org/10.1021/acs.iecr.3c03576. |
| 40 | | ✓ | Abu Fara, A.; Rayson, M. R.; Brent, G. F.; Oliver, T. K.; Stockenhuber, M.; Kennedy, E. M. Direct Aqueous Carbonation of Heat-Activated Lizardite; Effect of Particle Size and Solids Loading on Magnesite Yield. Minerals 2025, 15 (2), 155. https://doi.org/10.3390/min15020155. |
| 41 | ✓ | | Kim, D.-J.; and Chung, H.-S. Effect of Grinding on the Structure and Chemical Extraction of Metals from Serpentine. Particulate Science and Technology 2002, 20 (2), 159–168. https://doi.org/10.1080/02726350215336. |
| 42 | ✓ | | Saleem, M. H.; Rashid, M. I.; Khan, S. A.; Waleed, M.; Shahzad, M. U. Magnesium Extraction from Serpentine for Carbon Capture and Storage. Journal of the Pakistan Institute of Chemical Engineers 2024, 52 (2). https://doi.org/10.54693/piche.05226. |
| 43 | | ✓ | Benhelal, E.; Rashid, M. I.; Rayson, M. S.; Prigge, J.-D.; Molloy, S.; Brent, G. F.; Cote, A.; Stockenhuber, M.; Kennedy, E. M. Study on Mineral Carbonation of Heat Activated Lizardite at Pilot and Laboratory |

| | |
|---|---|
| | Scale. Journal of CO2 Utilization 2018, 26, 230–238. https://doi.org/10.1016/j.jcou.2018.05.015. |

[*]a only saprolite waste used

Table S3. Results for ANOVA Statistical Analysis with 100% of extraction efficiency data*

| Factor | SS** | D.f.*** | F-value | p-value |
|---|---|---|---|---|
| Factor1 | 435229.18 | 2 | 314.92 | 0.00 |
| Factor1:Factor2 | 263033.80 | 4 | 95.16 | 0.00 |
| Factor1:Factor3 | 207731.53 | 4 | 75.15 | 0.00 |
| Factor1:Factor4 | 202361.84 | 4 | 73.21 | 0.00 |
| Factor2:Factor5 | 112776.79 | 4 | 40.80 | 0.00 |
| Factor1:Factor2:Factor5 | 123972.16 | 8 | 22.43 | 0.00 |
| Factor1:Factor5 | 93105.34 | 4 | 33.68 | 0.00 |
| Factor1:Factor2:Factor4 | 107182.80 | 8 | 19.39 | 0.00 |
| Factor7 | 81201.43 | 2 | 58.75 | 0.00 |
| Factor1:Factor4:Factor5 | 89122.33 | 8 | 16.12 | 0.00 |
| Factor5:Factor6 | 73866.70 | 4 | 26.72 | 0.00 |
| Factor5 | 61262.75 | 2 | 44.33 | 0.00 |
| Factor2:Factor4:Factor5 | 82751.33 | 8 | 14.97 | 0.00 |
| Factor6:Factor7 | 63793.48 | 4 | 23.08 | 0.00 |
| Factor3:Factor7 | 59367.47 | 4 | 21.48 | 0.00 |
| Factor1:Factor2:Factor4:Factor5 | 92620.64 | 16 | 8.38 | 0.00 |
| Factor1:Factor2:Factor4:Factor7 | 81828.79 | 16 | 7.40 | 0.00 |
| Factor1:Factor6 | 50198.47 | 4 | 18.16 | 0.00 |
| Factor4:Factor6 | 41030.36 | 4 | 14.84 | 0.00 |
| Factor2:Factor4:Factor6 | 50256.19 | 8 | 9.09 | 0.00 |
| Factor4:Factor5 | 38453.34 | 4 | 13.91 | 0.00 |
| Factor3 | 31806.11 | 2 | 23.01 | 0.00 |
| Factor4:Factor7 | 36055.24 | 4 | 13.04 | 0.00 |
| Factor2 | 27121.18 | 2 | 19.62 | 0.00 |
| Factor2:Factor4 | 29555.78 | 4 | 10.69 | 0.00 |
| Factor5:Factor7 | 27468.62 | 4 | 9.94 | 0.00 |
| Factor1:Factor3:Factor4 | 34890.91 | 8 | 6.31 | 0.00 |
| Factor4 | 21709.56 | 2 | 15.71 | 0.00 |
| Factor6 | 21216.50 | 2 | 15.35 | 0.00 |
| Factor1:Factor2:Factor6 | 31845.00 | 8 | 5.76 | 0.00 |
| Factor2:Factor4:Factor7 | 30164.44 | 8 | 5.46 | 0.00 |
| Factor3:Factor6 | 22165.33 | 4 | 8.02 | 0.00 |
| Factor1:Factor5:Factor6 | 29467.35 | 8 | 5.33 | 0.00 |
| Factor1:Factor2:Factor4:Factor6 | 39783.50 | 16 | 3.60 | 0.00 |
| Factor1:Factor3:Factor4:Factor5 | 39581.05 | 16 | 3.58 | 0.00 |
| Factor1:Factor3:Factor5 | 27261.14 | 8 | 4.93 | 0.00 |
| Factor1:Factor2:Factor7 | 26928.13 | 8 | 4.87 | 0.00 |
| Factor3:Factor5 | 19508.21 | 4 | 7.06 | 0.00 |
| Factor2:Factor3:Factor5 | 26042.18 | 8 | 4.71 | 0.00 |
| Factor1:Factor4:Factor6 | 25416.55 | 8 | 4.60 | 0.00 |
| Factor2:Factor3:Factor5:Factor6 | 36758.77 | 16 | 3.32 | 0.00 |
| Factor4:Factor5:Factor7 | 24874.96 | 8 | 4.50 | 0.00 |

| | | | | |
|---|---|---|---|---|
| Factor1:Factor2:Factor3:Factor4 | 36553.34 | 16 | 3.31 | 0.00 |
| Factor3:Factor5:Factor6 | 24779.68 | 8 | 4.48 | 0.00 |
| Factor1:Factor2:Factor6:Factor7 | 34941.94 | 16 | 3.16 | 0.00 |
| Factor3:Factor4 | 16523.67 | 4 | 5.98 | 0.00 |
| Factor1:Factor2:Factor4:Factor5:Factor7 | 53298.66 | 32 | 2.410324 | 0.000281 |
| Factor2:Factor5:Factor7 | 21877.18 | 8 | 3.96 | 0.00 |
| Factor3:Factor6:Factor7 | 21868.63 | 8 | 3.96 | 0.00 |
| Factor4:Factor6:Factor7 | 21790.29 | 8 | 3.94 | 0.00 |
| Factor3:Factor4:Factor6 | 21481.66 | 8 | 3.89 | 0.00 |
| Factor1:Factor2:Factor3:Factor7 | 32226.39 | 16 | 2.91 | 0.00 |
| Factor3:Factor5:Factor7 | 20032.48 | 8 | 3.62 | 0.00 |
| Factor1:Factor3:Factor5:Factor6 | 29144.61 | 16 | 2.64 | 0.00 |
| Factor1:Factor4:Factor5:Factor6 | 28674.74 | 16 | 2.59 | 0.00 |
| Factor1:Factor4:Factor6:Factor7 | 28032.07 | 16 | 2.54 | 0.00 |
| Factor1:Factor3:Factor4:Factor5:Factor6 | 46121.45 | 32 | 2.085749 | 0.002126 |
| Factor2:Factor3 | 12253.90 | 4 | 4.43 | 0.00 |
| Factor2:Factor3:Factor4 | 17787.55 | 8 | 3.22 | 0.00 |
| Factor1:Factor4:Factor5:Factor7 | 27447.18 | 16 | 2.48 | 0.00 |
| Factor1:Factor2:Factor4:Factor6:Factor7 | 45492.07 | 32 | 2.057287 | 0.002529 |
| Factor2:Factor3:Factor4:Factor5 | 26511.26 | 16 | 2.40 | 0.00 |
| Factor2:Factor3:Factor6:Factor7 | 26290.96 | 16 | 2.38 | 0.00 |
| Factor1:Factor3:Factor7 | 16249.23 | 8 | 2.94 | 0.00 |
| Factor2:Factor6 | 10829.90 | 4 | 3.92 | 0.00 |
| Factor1:Factor5:Factor6:Factor7 | 25581.03 | 16 | 2.31 | 0.01 |
| Factor4:Factor5:Factor6 | 14855.47 | 8 | 2.69 | 0.01 |
| Factor1:Factor3:Factor5:Factor7 | 23559.99 | 16 | 2.13 | 0.01 |
| Factor1:Factor3:Factor6:Factor7 | 23333.72 | 16 | 2.11 | 0.01 |
| Factor1:Factor6:Factor7 | 14321.54 | 8 | 2.59 | 0.01 |
| Factor2:Factor3:Factor6 | 14282.64 | 8 | 2.58 | 0.01 |
| Factor1:Factor2:Factor5:Factor7 | 22866.12 | 16 | 2.07 | 0.01 |
| Factor1:Factor2:Factor4:Factor5:Factor6 | 38356.02 | 32 | 1.734574 | 0.016862 |
| Factor2:Factor4:Factor5:Factor7 | 22205.78 | 16 | 2.01 | 0.02 |
| Factor2:Factor3:Factor5:Factor6:Factor7 | 37640.76 | 32 | 1.702227 | 0.020211 |
| Factor1:Factor2:Factor3:Factor6 | 21478.37 | 16 | 1.94 | 0.02 |
| Factor1:Factor2:Factor5:Factor6 | 20399.09 | 16 | 1.85 | 0.03 |
| Factor1:Factor2:Factor3 | 11952.24 | 8 | 2.16 | 0.03 |
| Factor1:Factor7 | 7134.42 | 4 | 2.58 | 0.04 |

* Only the statistically significant (p-value < 0.05), out of the 127 different combinations of individual and interaction parameters. ** SS: Sum of squares; *** D.f.: degree of freedom

Table S4. Results for ANOVA statistical analysis with 80% of extraction efficiency data*

| Factor | SS** | D.f.*** | F-value | p-value |
|---|---|---|---|---|
| Factor6 | 463867.2 | 2 | 8406.88 | 0.00 |
| Factor1:Factor7 | 289700.4 | 4 | 2625.19 | 0.00 |
| Factor7 | 136904.2 | 2 | 2481.18 | 0.00 |
| Factor6:Factor7 | 267926.5 | 4 | 2427.88 | 0.00 |
| Factor1:Factor6:Factor7 | 337788.6 | 8 | 1530.47 | 0.00 |
| Factor4:Factor6 | 145517.1 | 4 | 1318.64 | 0.00 |
| Factor4 | 67646.83 | 2 | 1225.99 | 0.00 |
| Factor1:Factor6 | 129238.1 | 4 | 1171.12 | 0.00 |
| Factor1:Factor4:Factor6 | 203489.3 | 8 | 921.98 | 0.00 |
| Factor2 | 47723.59 | 2 | 864.92 | 0.00 |
| Factor1:Factor4:Factor7 | 171302 | 8 | 776.15 | 0.00 |
| Factor4:Factor6:Factor7 | 139053.3 | 8 | 630.03 | 0.00 |
| Factor1:Factor2:Factor7 | 131738.3 | 8 | 596.89 | 0.00 |
| Factor4:Factor7 | 49869.7 | 4 | 451.91 | 0.00 |
| Factor1:Factor2 | 46696.53 | 4 | 423.15 | 0.00 |
| Factor1:Factor3 | 45259.06 | 4 | 410.13 | 0.00 |
| Factor1:Factor4:Factor6:Factor7 | 161699.4 | 16 | 366.32 | 0.00 |
| Factor2:Factor7 | 37396.47 | 4 | 338.88 | 0.00 |
| Factor3 | 18620.09 | 2 | 337.46 | 0.00 |
| Factor1 | 17743.78 | 2 | 321.58 | 0.00 |
| Factor3:Factor6:Factor7 | 65379.75 | 8 | 296.23 | 0.00 |
| Factor1:Factor2:Factor4:Factor6:Factor7 | 257127.9 | 32 | 291.25 | 0.00 |
| Factor1:Factor2:Factor6 | 61551.57 | 8 | 278.88 | 0.00 |
| Factor1:Factor4 | 30262.1 | 4 | 274.23 | 0.00 |
| Factor3:Factor4 | 28373.97 | 4 | 257.12 | 0.00 |
| Factor1:Factor2:Factor4:Factor7 | 103340.3 | 16 | 234.11 | 0.00 |
| Factor2:Factor6:Factor7 | 44274.93 | 8 | 200.60 | 0.00 |
| Factor2:Factor4:Factor7 | 41120.75 | 8 | 186.31 | 0.00 |
| Factor1:Factor3:Factor6 | 38470.99 | 8 | 174.31 | 0.00 |
| Factor1:Factor2:Factor4 | 38209.08 | 8 | 173.12 | 0.00 |
| Factor2:Factor6 | 18394.92 | 4 | 166.69 | 0.00 |
| Factor1:Factor2:Factor6:Factor7 | 72907.1 | 16 | 165.17 | 0.00 |
| Factor2:Factor4:Factor6:Factor7 | 70593.84 | 16 | 159.93 | 0.00 |
| Factor2:Factor4:Factor6 | 30530.52 | 8 | 138.33 | 0.00 |
| Factor2:Factor4 | 13488.58 | 4 | 122.23 | 0.00 |
| Factor2:Factor3:Factor6 | 24643.54 | 8 | 111.66 | 0.00 |
| Factor1:Factor3:Factor7 | 21306.48 | 8 | 96.54 | 0.00 |
| Factor3:Factor7 | 8886.915 | 4 | 80.53 | 0.00 |
| Factor1:Factor2:Factor4:Factor6 | 27488.86 | 16 | 62.27 | 0.00 |
| Factor1:Factor3:Factor4:Factor7 | 23641.26 | 16 | 53.56 | 0.00 |
| Factor1:Factor3:Factor6:Factor7 | 22730.54 | 16 | 51.49 | 0.00 |
| Factor1:Factor3:Factor4 | 11349.42 | 8 | 51.42 | 0.00 |

| | | | | |
|---|---|---|---|---|
| Factor3:Factor4:Factor6:Factor7 | 17466.28 | 16 | 39.57 | 0.00 |
| Factor1:Factor2:Factor3:Factor4:Factor7 | 34830.82 | 32 | 39.45 | 0.00 |
| Factor3:Factor4:Factor7 | 8504.327 | 8 | 38.53 | 0.00 |
| Factor5:Factor6:Factor7 | 8371.773 | 8 | 37.93 | 0.00 |
| Factor2:Factor3 | 4149.739 | 4 | 37.60 | 0.00 |
| Factor1:Factor3:Factor4:Factor6:Factor7 | 30880.67 | 32 | 34.98 | 0.00 |
| Factor1:Factor3:Factor4:Factor6 | 14480.53 | 16 | 32.80 | 0.00 |
| Factor2:Factor3:Factor7 | 7182.077 | 8 | 32.54 | 0.00 |
| Factor3:Factor6 | 3363.712 | 4 | 30.48 | 0.00 |
| Factor1:Factor2:Factor3:Factor4:Factor6:Factor7 | 51138.67 | 64 | 28.96 | 0.00 |
| Factor2:Factor3:Factor6:Factor7 | 12313.14 | 16 | 27.89 | 0.00 |
| Factor2:Factor3:Factor4:Factor6 | 11279.72 | 16 | 25.55 | 0.00 |
| Factor2:Factor3:Factor4:Factor6:Factor7 | 17731.9 | 32 | 20.09 | 0.00 |
| Factor1:Factor2:Factor3:Factor4 | 8403.042 | 16 | 19.04 | 0.00 |
| Factor2:Factor3:Factor4:Factor7 | 8125.047 | 16 | 18.41 | 0.00 |
| Factor2:Factor3:Factor4 | 3735.507 | 8 | 16.93 | 0.00 |
| Factor1:Factor2:Factor3:Factor7 | 6936.264 | 16 | 15.71 | 0.00 |
| Factor1:Factor2:Factor3:Factor6:Factor7 | 13755.17 | 32 | 15.58 | 0.00 |
| Factor5 | 841.8371 | 2 | 15.26 | 0.00 |
| Factor3:Factor4:Factor6 | 3350.744 | 8 | 15.18 | 0.00 |
| Factor1:Factor2:Factor3:Factor6 | 6443.41 | 16 | 14.60 | 0.00 |
| Factor1:Factor2:Factor3:Factor4:Factor6 | 11485.4 | 32 | 13.01 | 0.00 |
| Factor2:Factor4:Factor5 | 2839.326 | 8 | 12.86 | 0.00 |
| Factor1:Factor5:Factor6 | 2616.883 | 8 | 11.86 | 0.00 |
| Factor1:Factor5 | 1213.708 | 4 | 11.00 | 0.00 |
| Factor4:Factor5 | 1118.445 | 4 | 10.14 | 0.00 |
| Factor1:Factor4:Factor5 | 2183.909 | 8 | 9.89 | 0.00 |
| Factor5:Factor7 | 934.1856 | 4 | 8.47 | 0.00 |
| Factor1:Factor4:Factor5:Factor7 | 3692.728 | 16 | 8.37 | 0.00 |
| Factor1:Factor2:Factor3 | 1682.86 | 8 | 7.62 | 0.00 |
| Factor1:Factor3:Factor5:Factor6 | 3138.2 | 16 | 7.11 | 0.00 |
| Factor1:Factor5:Factor6:Factor7 | 3059.189 | 16 | 6.93 | 0.00 |
| Factor1:Factor5:Factor7 | 1386.761 | 8 | 6.28 | 0.00 |
| Factor1:Factor2:Factor4:Factor5:Factor7 | 5012.384 | 32 | 5.68 | 0.00 |
| Factor2:Factor5:Factor6 | 1249.799 | 8 | 5.66 | 0.00 |
| Factor3:Factor5:Factor7 | 1226.209 | 8 | 5.56 | 0.00 |
| Factor2:Factor5:Factor7 | 1208.689 | 8 | 5.48 | 0.00 |
| Factor1:Factor4:Factor5:Factor6:Factor7 | 4249.765 | 32 | 4.81 | 0.00 |
| Factor3:Factor5 | 516.5563 | 4 | 4.68 | 0.00 |
| Factor1:Factor3:Factor5 | 1028.426 | 8 | 4.66 | 0.00 |
| Factor4:Factor5:Factor7 | 918.5111 | 8 | 4.16 | 0.00 |
| Factor1:Factor3:Factor5:Factor7 | 1753.38 | 16 | 3.97 | 0.00 |
| Factor1:Factor2:Factor4:Factor5 | 1548.484 | 16 | 3.51 | 0.00 |
| Factor3:Factor4:Factor5:Factor6 | 1474.037 | 16 | 3.34 | 0.00 |
| Factor1:Factor3:Factor5:Factor6:Factor7 | 2928.165 | 32 | 3.32 | 0.00 |

| | | | | |
|---|---|---|---|---|
| Factor3:Factor4:Factor5:Factor7 | 1388.846 | 16 | 3.15 | 0.00 |
| Factor1:Factor2:Factor4:Factor5:Factor6:Factor7 | 5372.848 | 64 | 3.04 | 0.00 |
| Factor1:Factor2:Factor5:Factor7 | 1313.751 | 16 | 2.98 | 0.00 |
| Factor3:Factor5:Factor6 | 655.0486 | 8 | 2.97 | 0.00 |
| Factor5:Factor6 | 321.9784 | 4 | 2.92 | 0.02 |
| Factor3:Factor5:Factor6:Factor7 | 1216.106 | 16 | 2.76 | 0.00 |
| Factor4:Factor5:Factor6:Factor7 | 1112.096 | 16 | 2.52 | 0.00 |
| Factor2:Factor4:Factor5:Factor7 | 1033.374 | 16 | 2.34 | 0.00 |
| Factor1:Factor2:Factor5:Factor6:Factor7 | 1995.094 | 32 | 2.26 | 0.00 |
| Factor1:Factor3:Factor4:Factor5:Factor6 | 1976.679 | 32 | 2.24 | 0.00 |
| Factor2:Factor3:Factor4:Factor5 | 956.3998 | 16 | 2.17 | 0.01 |
| Factor2:Factor5 | 234.3263 | 4 | 2.12 | 0.08 |
| Factor1:Factor4:Factor5:Factor6 | 883.8573 | 16 | 2.00 | 0.02 |

[*] Only the first 100 data points are shown, out of the 127 different combinations of individual and interaction parameters. [**]SS: Sum of squares; [***] D.f.: degree of freedom

Table S5: Results for ANOVA statistical analysis with 100% of $CO_2$ sequestration capacity data-direct carbonation*

| Factor | SS* | D.f.*** | F-value | p-value |
|---|---|---|---|---|
| Factor6 | 190882600 | 2 | 33307 | 0.0000 |
| Factor3 | 180536100 | 2 | 31502 | 0.0000 |
| Factor8 | 40308590 | 2 | 7034 | 0.0000 |
| Factor1 | 25885200 | 2 | 4517 | 0.0000 |
| Factor7 | 22448400 | 2 | 3917 | 0.0000 |
| Factor2:Factor6 | 43256760 | 4 | 3774 | 0.0000 |
| Factor5 | 18885520 | 2 | 3295 | 0.0000 |
| Factor3:Factor6 | 33210160 | 4 | 2897.447 | 0.0000 |
| Factor4:Factor6 | 28719010 | 4 | 2506 | 0.0000 |
| Factor1:Factor5:Factor9 | 33382840 | 8 | 1456.257 | 0.0000 |
| Factor5:Factor6 | 16439460 | 4 | 1434 | 0.0000 |
| Factor2:Factor4 | 16220360 | 4 | 1415.159 | 0.0000 |
| Factor5:Factor6:Factor9 | 31531720 | 8 | 1376 | 0.0000 |
| Factor2:Factor7 | 15626510 | 4 | 1363 | 0.0000 |
| Factor2 | 7339357 | 2 | 1281 | 0.0000 |
| Factor1:Factor6 | 14218490 | 4 | 1241 | 0.0000 |
| Factor1:Factor2:Factor5 | 27268130 | 8 | 1189.515 | 0.0000 |
| Factor9 | 6440066 | 2 | 1124 | 0.0000 |
| Factor6:Factor8 | 10497210 | 4 | 916 | 0.0000 |
| Factor3:Factor8 | 8813374 | 4 | 769 | 0.0000 |
| Factor2:Factor5 | 8589872 | 4 | 749 | 0.0000 |
| Factor3:Factor7 | 7816980 | 4 | 682 | 0.0000 |
| Factor5:Factor7 | 7537181 | 4 | 658 | 0.0000 |
| Factor1:Factor5 | 6881666 | 4 | 600 | 0.0000 |
| Factor4:Factor7 | 6064679 | 4 | 529 | 0.0000 |
| Factor4:Factor5 | 5916404 | 4 | 516.1815 | 0.0000 |
| Factor5:Factor8 | 5752920 | 4 | 502 | 0.0000 |
| Factor1:Factor8 | 5637216 | 4 | 491.8236 | 0.0000 |
| Factor1:Factor5:Factor8 | 10966700 | 8 | 478.3993 | 0.0000 |
| Factor6:Factor8:Factor9 | 10427960 | 8 | 455 | 0.0000 |
| Factor3:Factor5:Factor9 | 9860423 | 8 | 430 | 0.0000 |
| Factor6:Factor7 | 4898769 | 4 | 427.3971 | 0.0000 |
| Factor3:Factor5:Factor6:Factor9 | 17611960 | 16 | 384 | 0.0000 |
| Factor4:Factor9 | 3862189 | 4 | 337 | 0.0000 |
| Factor1:Factor5:Factor6 | 7545201 | 8 | 329.1436 | 0.0000 |
| Factor1:Factor3:Factor5 | 7305198 | 8 | 318.674 | 0.0000 |
| Factor6:Factor9 | 3422175 | 4 | 298.5704 | 0.0000 |
| Factor1:Factor5:Factor6:Factor9 | 13461520 | 16 | 294 | 0.0000 |
| Factor3:Factor4:Factor9 | 6459642 | 8 | 282 | 0.0000 |
| Factor3:Factor6:Factor8 | 6365387 | 8 | 278 | 0.0000 |
| Factor5:Factor9 | 3076252 | 4 | 268 | 0.0000 |

| | | | | |
|---|---|---|---|---|
| Factor3:Factor5:Factor6 | 5683196 | 8 | 248 | 0.00 |
| Factor1:Factor5:Factor7 | 5593864 | 8 | 244.0206 | 0.00 |
| Factor1:Factor7 | 2751942 | 4 | 240 | 0.0000 |
| Factor2:Factor8 | 2614349 | 4 | 228 | 0.0000 |
| Factor5:Factor6:Factor7 | 5136683 | 8 | 224.0771 | 0.0000 |
| Factor1:Factor4:Factor6 | 4989739 | 8 | 218 | 0.0000 |
| Factor2:Factor5:Factor9 | 4532990 | 8 | 197.7422 | 0.0000 |
| Factor1:Factor2:Factor5:Factor9 | 8720492 | 16 | 190.2066 | 0.0000 |
| Factor2:Factor6:Factor7 | 4299898 | 8 | 188 | 0.0000 |
| Factor3:Factor4:Factor6 | 4234039 | 8 | 184.7011 | 0.0000 |
| Factor1:Factor6:Factor9 | 4167053 | 8 | 182 | 0.0000 |
| Factor2:Factor4:Factor6 | 4090154 | 8 | 178.4244 | 0.0000 |
| Factor1:Factor2:Factor3 | 3860426 | 8 | 168 | 0.0000 |
| Factor2:Factor3 | 1854251 | 4 | 162 | 0.0000 |
| Factor3:Factor6:Factor9 | 3503358 | 8 | 153 | 0.0000 |
| Factor4 | 770040.2 | 2 | 134 | 0.0000 |
| Factor2:Factor5:Factor6 | 3014119 | 8 | 131.4846 | 0.0000 |
| Factor8:Factor9 | 1490980 | 4 | 130 | 0.0000 |
| Factor4:Factor5:Factor6 | 2957902 | 8 | 129.0323 | 0.0000 |
| Factor1:Factor9 | 1440526 | 4 | 126 | 0.0000 |
| Factor1:Factor3:Factor5:Factor9 | 5727403 | 16 | 124.923 | 0.00 |
| Factor7:Factor8:Factor9 | 2751173 | 8 | 120 | 0.0000 |
| Factor3:Factor9 | 1339804 | 4 | 116.8923 | 0.0000 |
| Factor5:Factor6:Factor8:Factor9 | 5354998 | 16 | 116.8003 | 0.0000 |
| Factor1:Factor4:Factor7 | 2586089 | 8 | 113 | 0.0000 |
| Factor3:Factor5 | 1232761 | 4 | 108 | 0.0000 |
| Factor3:Factor4 | 1194883 | 4 | 104.2486 | 0.0000 |
| Factor1:Factor7:Factor9 | 2385633 | 8 | 104.0683 | 0.0000 |
| Factor1:Factor5:Factor8:Factor9 | 4691497 | 16 | 102.3284 | 0.0000 |
| Factor3:Factor5:Factor7 | 2195324 | 8 | 96 | 0.0000 |
| Factor1:Factor3:Factor5:Factor6 | 4177290 | 16 | 91.11278 | 0.0000 |
| Factor1:Factor5:Factor7:Factor9 | 4015318 | 16 | 87.57992 | 0.0000 |
| Factor1:Factor4:Factor5:Factor6 | 3893720 | 16 | 84.92769 | 0.0000 |
| Factor1:Factor3:Factor4 | 1925221 | 8 | 84 | 0.0000 |
| Factor1:Factor7:Factor8 | 1876199 | 8 | 82 | 0.0000 |
| Factor2:Factor9 | 932763.7 | 4 | 81.37973 | 0.0000 |
| Factor2:Factor3:Factor6 | 1855784 | 8 | 80.9547 | 0.0000 |
| Factor2:Factor7:Factor9 | 1821491 | 8 | 79 | 0.0000 |
| Factor1:Factor2:Factor6 | 1786334 | 8 | 78 | 0.0000 |
| Factor2:Factor3:Factor5 | 1773278 | 8 | 77 | 0.0000 |
| Factor2:Factor8:Factor9 | 1737496 | 8 | 75.79463 | 0.0000 |
| Factor1:Factor2:Factor5:Factor6 | 3337668 | 16 | 72.79938 | 0.0000 |
| Factor3:Factor7:Factor9 | 1630340 | 8 | 71 | 0.0000 |
| Factor5:Factor6:Factor7:Factor9 | 3229116 | 16 | 70.4317 | 0.0000 |
| Factor1:Factor4:Factor5 | 1600523 | 8 | 69.81949 | 0.0000 |

| | | | | |
|---|---|---|---|---|
| Factor1:Factor2:Factor3:Factor5 | 3118172 | 16 | 68 | 0.0000 |
| Factor1:Factor3:Factor5:Factor6:Factor9 | 6002901 | 32 | 65.46599 | 0.00 |
| Factor2:Factor3:Factor7 | 1448065 | 8 | 63.16882 | 0.0000 |
| Factor1:Factor2:Factor5:Factor7 | 2739040 | 16 | 59.74244 | 0.0000 |
| Factor1:Factor3:Factor6:Factor9 | 2689141 | 16 | 58.65407 | 0.0000 |
| Factor2:Factor4:Factor5 | 1337114 | 8 | 58.32879 | 0.0000 |
| Factor1:Factor2:Factor9 | 1308302 | 8 | 57 | 0.0000 |
| Factor1:Factor2:Factor5:Factor6:Factor9 | 5214825 | 32 | 56.87145 | 0.0000 |
| Factor2:Factor3:Factor5:Factor6 | 2567403 | 16 | 55.9988 | 0.0000 |
| Factor1:Factor2:Factor4 | 1264291 | 8 | 55 | 0.0000 |
| Factor1:Factor4:Factor5:Factor9 | 2528033 | 16 | 55.14008 | 0.0000 |
| Factor2:Factor5:Factor7 | 1262031 | 8 | 55.05347 | 0.0000 |
| Factor1:Factor3 | 629132.2 | 4 | 55 | 0.0000 |
| Factor1:Factor5:Factor6:Factor7 | 2422330 | 16 | 52.83453 | 0.0000 |

* Only the first 100 data points are shown, out of the 511 different combinations of individual and interaction parameters. **SS: Sum of squares; *** D.f.: degree of freedom

Table S6. Results for ANOVA statistical analysis with 80% of $CO_2$ sequestration capacity data-direct carbonation

| Factor | SS* | D.f.*** | F-value | p-value |
|---|---|---|---|---|
| Factor8 | 85335070 | 2 | 333235.5 | 0.00 |
| Factor7 | 37802070 | 2 | 147618.0 | 0.00 |
| Factor2 | 29549680 | 2 | 115392.2 | 0.00 |
| Factor9 | 22230490 | 2 | 86810.61 | 0.00 |
| Factor5:Factor7 | 40848490 | 4 | 79757.17 | 0.00 |
| Factor1 | 14374630 | 2 | 56133.29 | 0.00 |
| Factor3 | 13407920 | 2 | 52358.27 | 0.00 |
| Factor6:Factor7 | 26472310 | 4 | 51687.52 | 0.00 |
| Factor5 | 11880130 | 2 | 46392.21 | 0.00 |
| Factor5:Factor7:Factor8 | 45717500 | 8 | 44631.99 | 0.00 |
| Factor7:Factor8 | 13575940 | 4 | 26507.19 | 0.00 |
| Factor6:Factor7:Factor8 | 23623530 | 8 | 23062.61 | 0.00 |
| Factor7:Factor9 | 9767763 | 4 | 19071.68 | 0.00 |
| Factor6 | 4551241 | 2 | 17772.71 | 0.00 |
| Factor5:Factor8 | 8653136 | 4 | 16895.35 | 0.00 |
| Factor4:Factor8 | 8193512 | 4 | 15997.93 | 0.00 |
| Factor1:Factor8:Factor9 | 16118340 | 8 | 15735.63 | 0.00 |
| Factor1:Factor6 | 6676516 | 4 | 13035.98 | 0.00 |
| Factor1:Factor7:Factor8 | 11789470 | 8 | 11509.54 | 0.00 |
| Factor1:Factor9 | 5734670 | 4 | 11197.01 | 0.00 |
| Factor1:Factor5:Factor6 | 10177910 | 8 | 9936.25 | 0.00 |
| Factor1:Factor8 | 5046609 | 4 | 9853.57 | 0.00 |
| Factor4:Factor8:Factor9 | 9572205 | 8 | 9344.92 | 0.00 |
| Factor1:Factor7 | 4613818 | 4 | 9008.54 | 0.00 |
| Factor1:Factor4 | 4419991 | 4 | 8630.09 | 0.00 |
| Factor1:Factor5:Factor9 | 8672756 | 8 | 8466.83 | 0.00 |
| Factor5:Factor6 | 4301355 | 4 | 8398.45 | 0.00 |
| Factor8:Factor9 | 3467153 | 4 | 6769.66 | 0.00 |
| Factor6:Factor7:Factor9 | 6924800 | 8 | 6760.38 | 0.00 |
| Factor1:Factor7:Factor8:Factor9 | 13332380 | 16 | 6507.91 | 0.00 |
| Factor6:Factor8:Factor9 | 5519522 | 8 | 5388.47 | 0.00 |
| Factor5:Factor6:Factor7 | 5152307 | 8 | 5029.97 | 0.00 |
| Factor1:Factor5 | 2488669 | 4 | 4859.16 | 0.00 |
| Factor6:Factor8 | 2453200 | 4 | 4789.90 | 0.00 |
| Factor1:Factor4:Factor6 | 3949398 | 8 | 3855.62 | 0.00 |
| Factor4:Factor5 | 1942074 | 4 | 3791.92 | 0.00 |
| Factor1:Factor4:Factor7 | 3740597 | 8 | 3651.78 | 0.00 |
| Factor1:Factor5:Factor7 | 3464429 | 8 | 3382.17 | 0.00 |
| Factor4:Factor6:Factor7 | 3446894 | 8 | 3365.05 | 0.00 |
| Factor4 | 843273 | 2 | 3293.00 | 0.00 |
| Factor3:Factor6:Factor7:Factor22 | 6028450 | 16 | 2942.66 | 0.00 |

| | | | | |
|---|---|---|---|---|
| Factor7:Factor8:Factor9 | 2687499 | 8 | 2623.69 | 0.00 |
| Factor4:Factor7 | 1332796 | 4 | 2602.30 | 0.00 |
| Factor5:Factor9 | 1317383 | 4 | 2572.21 | 0.00 |
| Factor6:Factor9 | 1291085 | 4 | 2520.86 | 0.00 |
| Factor1:Factor6:Factor7:Factor8 | 3968377 | 16 | 1937.08 | 0.00 |
| Factor1:Factor6:Factor7 | 1796526 | 8 | 1753.87 | 0.00 |
| Factor1:Factor7:Factor9 | 1721553 | 8 | 1680.68 | 0.00 |
| Factor3:Factor4:Factor8 | 1706092 | 8 | 1665.58 | 0.00 |
| Factor4:Factor6 | 801323 | 4 | 1564.59 | 0.00 |
| Factor1:Factor5:Factor8 | 1461758 | 8 | 1427.05 | 0.00 |
| Factor5:Factor8:Factor9 | 1430868 | 8 | 1396.89 | 0.00 |
| Factor4:Factor5:Factor8 | 1398391 | 8 | 1365.19 | 0.00 |
| Factor2:Factor5:Factor7 | 1329261 | 8 | 1297.70 | 0.00 |
| Factor5:Factor6:Factor9 | 1326191 | 8 | 1294.70 | 0.00 |
| Factor4:Factor9 | 653405 | 4 | 1275.78 | 0.00 |
| Factor1:Factor4:Factor7:Factor8 | 2529320 | 16 | 1234.63 | 0.00 |
| Factor1:Factor5:Factor7:Factor9 | 2506830 | 16 | 1223.65 | 0.00 |
| Factor2:Factor5 | 616341 | 4 | 1203.41 | 0.00 |
| Factor5:Factor6:Factor8 | 1188699 | 8 | 1160.47 | 0.00 |
| Factor2:Factor3 | 594068 | 4 | 1159.93 | 0.00 |
| Factor1:Factor4:Factor9 | 1183498 | 8 | 1155.40 | 0.00 |
| Factor2:Factor7 | 576594 | 4 | 1125.81 | 0.00 |
| Factor1:Factor4:Factor8 | 1059612 | 8 | 1034.45 | 0.00 |
| Factor2:Factor6:Factor7 | 1054033 | 8 | 1029.01 | 0.00 |
| Factor1:Factor6:Factor9 | 1038542 | 8 | 1013.88 | 0.00 |
| Factor1:Factor5:Factor6:Factor8 | 1979574 | 16 | 966.29 | 0.00 |
| Factor1:Factor6:Factor8:Factor9 | 1964735 | 16 | 959.04 | 0.00 |
| Factor1:Factor5:Factor6:Factor7 | 1768105 | 16 | 863.06 | 0.00 |
| Factor3:Factor7 | 436733 | 4 | 852.73 | 0.00 |
| Factor1:Factor3:Factor9 | 854707.2 | 8 | 834.41 | 0.00 |
| Factor3:Factor6:Factor7:Factor147 | 3377264 | 32 | 824.27 | 0.00 |
| Factor3:Factor4 | 407973 | 4 | 796.57 | 0.00 |
| Factor1:Factor4:Factor6:Factor7 | 1619633 | 16 | 790.59 | 0.00 |
| Factor2:Factor7:Factor8 | 809171.2 | 8 | 789.96 | 0.00 |
| Factor3:Factor6:Factor7:Factor26 | 1522996 | 16 | 743.42 | 0.00 |
| Factor1:Factor4:Factor8:Factor9 | 1477908 | 16 | 721.41 | 0.00 |
| Factor3:Factor6:Factor7:Factor23 | 1464333 | 16 | 714.78 | 0.00 |
| Factor3:Factor6:Factor7:Factor92 | 2904319 | 32 | 708.84 | 0.00 |
| Factor3:Factor6:Factor7:Factor20 | 1441115 | 16 | 703.45 | 0.00 |
| Factor3:Factor5:Factor7:Factor8 | 1435375 | 16 | 700.65 | 0.00 |
| Factor3:Factor6:Factor7:Factor12 | 1430948 | 16 | 698.49 | 0.00 |
| Factor1:Factor3 | 351044 | 4 | 685.42 | 0.00 |
| Factor3:Factor6:Factor7:Factor18 | 1372160 | 16 | 669.79 | 0.00 |
| Factor4:Factor6:Factor8 | 655002.2 | 8 | 639.45 | 0.00 |
| Factor3:Factor5:Factor8 | 608733 | 8 | 594.28 | 0.00 |

| | | | | |
|---|---|---|---|---|
| Factor3:Factor8 | 299507 | 4 | 584.79 | 0.00 |
| Factor2:Factor4 | 276060 | 4 | 539.01 | 0.00 |
| Factor4:Factor7:Factor8 | 524569.8 | 8 | 512.11 | 0.00 |
| Factor1:Factor4:Factor5:Factor7 | 1044193 | 16 | 509.70 | 0.00 |
| Factor1:Factor5:Factor7:Factor8 | 1012689 | 16 | 494.32 | 0.00 |
| Factor1:Factor5:Factor8:Factor9 | 974795 | 16 | 475.82 | 0.00 |
| Factor1:Factor6:Factor8 | 486166.9 | 8 | 474.62 | 0.00 |
| Factor3:Factor6:Factor7:Factor95 | 1814688 | 32 | 442.90 | 0.00 |
| Factor1:Factor5:Factor6:Factor9 | 857446.6 | 16 | 418.54 | 0.00 |
| Factor1:Factor3:Factor7:Factor8 | 837647.4 | 16 | 408.88 | 0.00 |
| Factor1:Factor6:Factor7:Factor9 | 827363 | 16 | 403.86 | 0.00 |
| Factor3:Factor6:Factor7:Factor11 | 792869.1 | 16 | 387.02 | 0.00 |
| Factor3:Factor6:Factor7:Factor81 | 1560649 | 32 | 380.90 | 0.00 |
| Factor1:Factor4:Factor5:Factor9 | 779631.1 | 16 | 380.56 | 0.00 |

* Only the first 100 data points are shown, out of the 511 different combinations of individual and interaction parameters. **SS: Sum of squares; *** D.f.: degree of freedom

Table S7: Results for ANOVA statistical analysis for 100% of $CO_2$ sequestration capacity data-indirect carbonation*

| Factor | SS* | D.f.*** | F-value | p-value |
|---|---|---|---|---|
| Factor5 | 2462357 | 2 | 216 | 0.000 |
| Factor1 | 2004652 | 2 | 176 | 0.000 |
| Factor3:Factor5 | 2050431 | 4 | 90 | 0.000 |
| Factor4 | 501861.2 | 2 | 44 | 0.000 |
| Factor3 | 382238.4 | 2 | 34 | 0.000 |
| Factor1:Factor5 | 518567.4 | 4 | 23 | 0.000 |
| Factor1:Factor3:Factor5 | 924965.7 | 8 | 20.27173 | 0.000 |
| Factor2:Factor3 | 380177.7 | 4 | 17 | 0.000 |
| Factor1:Factor2 | 297813.4 | 4 | 13 | 0.000 |
| Factor4:Factor5 | 255991.3 | 4 | 11 | 0.000 |
| Factor1:Factor2:Factor4 | 393311.8 | 8 | 9 | 0.000 |
| Factor2 | 97218.95 | 2 | 9 | 0.001 |
| Factor1:Factor3 | 186261.1 | 4 | 8 | 0.000 |
| Factor2:Factor4 | 175282.7 | 4 | 8 | 0.000 |
| Factor3:Factor4 | 171322.7 | 4 | 8 | 0.000 |
| Factor1:Factor2:Factor3 | 313216.1 | 8 | 7 | 0.000 |
| Factor1:Factor3:Factor4 | 308597.4 | 8 | 7 | 0.000 |
| Factor2:Factor5 | 113677.5 | 4 | 5 | 0.003 |
| Factor1:Factor2:Factor5 | 219164.1 | 8 | 5 | 0.001 |
| Factor2:Factor3:Factor4 | 185610.9 | 8 | 4 | 0.002 |
| Factor1:Factor4:Factor5 | 161062.8 | 8 | 4 | 0.005 |
| Factor1:Factor3:Factor4:Factor5 | 283819 | 16 | 3.110117 | 0.003 |
| Factor2:Factor3:Factor5 | 123416.4 | 8 | 2.704818 | 0.021 |
| Factor1:Factor2:Factor4:Factor5 | 246003.9 | 16 | 3 | 0.008 |
| Factor2:Factor4:Factor5 | 120602.2 | 8 | 3 | 0.024 |
| Factor3:Factor4:Factor5 | 117350 | 8 | 3 | 0.027 |

* Only the statistically significant (p-value < 0.05), out of the 31 different combinations of individual and interaction parameters. ** SS: Sum of squares; *** D.f.: degree of freedom

Table S8: Results for ANOVA statistical analysis with 80% of $CO_2$ sequestration capacity data-indirect carbonation*

| Factor | SS* | D.f.*** | F-value | p-value |
|---|---|---|---|---|
| Factor5 | 2988836 | 2 | 634.64 | 0.00 |
| Factor3:Factor5 | 3355059 | 4 | 356.20 | 0.00 |
| Factor3 | 1244928 | 2 | 264.35 | 0.00 |
| Factor1 | 850389 | 2 | 180.57 | 0.00 |
| Factor4 | 306534 | 2 | 65.09 | 0.00 |
| Factor1:Factor3:Factor5 | 1222297 | 8 | 64.88 | 0.00 |
| Factor3:Factor4 | 554852 | 4 | 58.91 | 0.00 |
| Factor1:Factor5 | 525251 | 4 | 55.77 | 0.00 |
| Factor2:Factor3 | 404795 | 4 | 42.98 | 0.00 |
| Factor4:Factor5 | 326285.6 | 4 | 34.64 | 0.00 |
| Factor1:Factor2 | 142892 | 4 | 15.17 | 0.00 |
| Factor2 | 63235 | 2 | 13.43 | 0.00 |
| Factor1:Factor3:Factor4 | 233484.7 | 8 | 12.39 | 0.00 |
| Factor3:Factor4:Factor5 | 227949 | 8 | 12.10 | 0.00 |
| Factor1:Factor3:Factor4:Factor5 | 435809 | 16 | 11.57 | 0.00 |
| Factor1:Factor4 | 106488 | 4 | 11.31 | 0.00 |
| Factor2:Factor5 | 106245 | 4 | 11.28 | 0.00 |
| Factor1:Factor3 | 104713 | 4 | 11.12 | 0.00 |
| Factor2:Factor4:Factor5 | 181693 | 8 | 9.65 | 0.00 |
| Factor1:Factor2:Factor4 | 146462 | 8 | 7.77 | 0.00 |
| Factor2:Factor3:Factor4 | 134200 | 8 | 7.12 | 0.00 |
| Factor2:Factor3:Factor5 | 114091 | 8 | 6.06 | 0.00 |
| Factor1:Factor2:Factor3 | 100852 | 8 | 5.35 | 0.00 |
| Factor2:Factor4 | 39805 | 4 | 4.23 | 0.01 |
| Factor1:Factor4:Factor5 | 45467 | 8 | 2.41 | 0.04 |
| Factor1:Factor2:Factor4:Factor5 | 85561 | 16 | 2.27 | 0.02 |
| Factor2:Factor3:Factor4:Factor5 | 84710 | 16 | 2.25 | 0.02 |

* Only the statistically significant (p-value < 0.05), out of the 31 different combinations of individual and interaction parameters. ** SS: Sum of squares; *** D.f.: degree of freedom

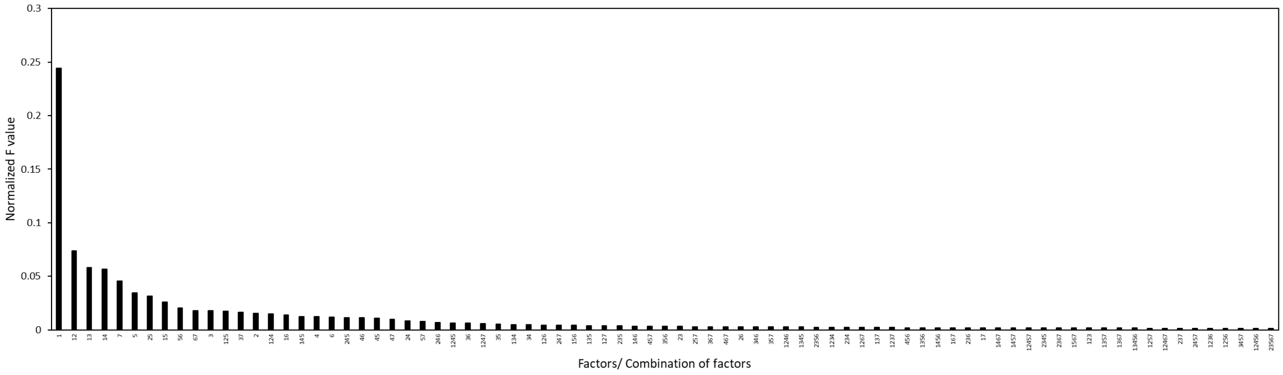


Figure S5. Effect of combined factors on the extraction efficiency with 100% data used for modelling.

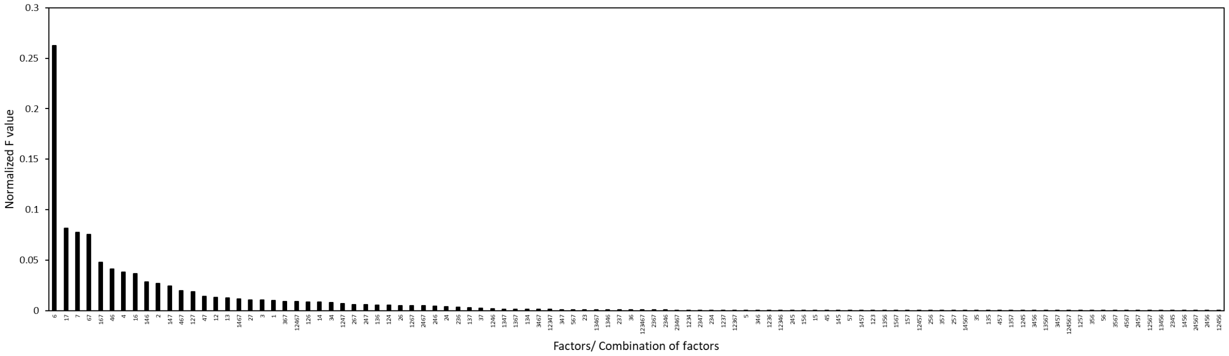


Figure S6. Effect of combined significant factors on the extraction efficiency with 80% data used for modelling.

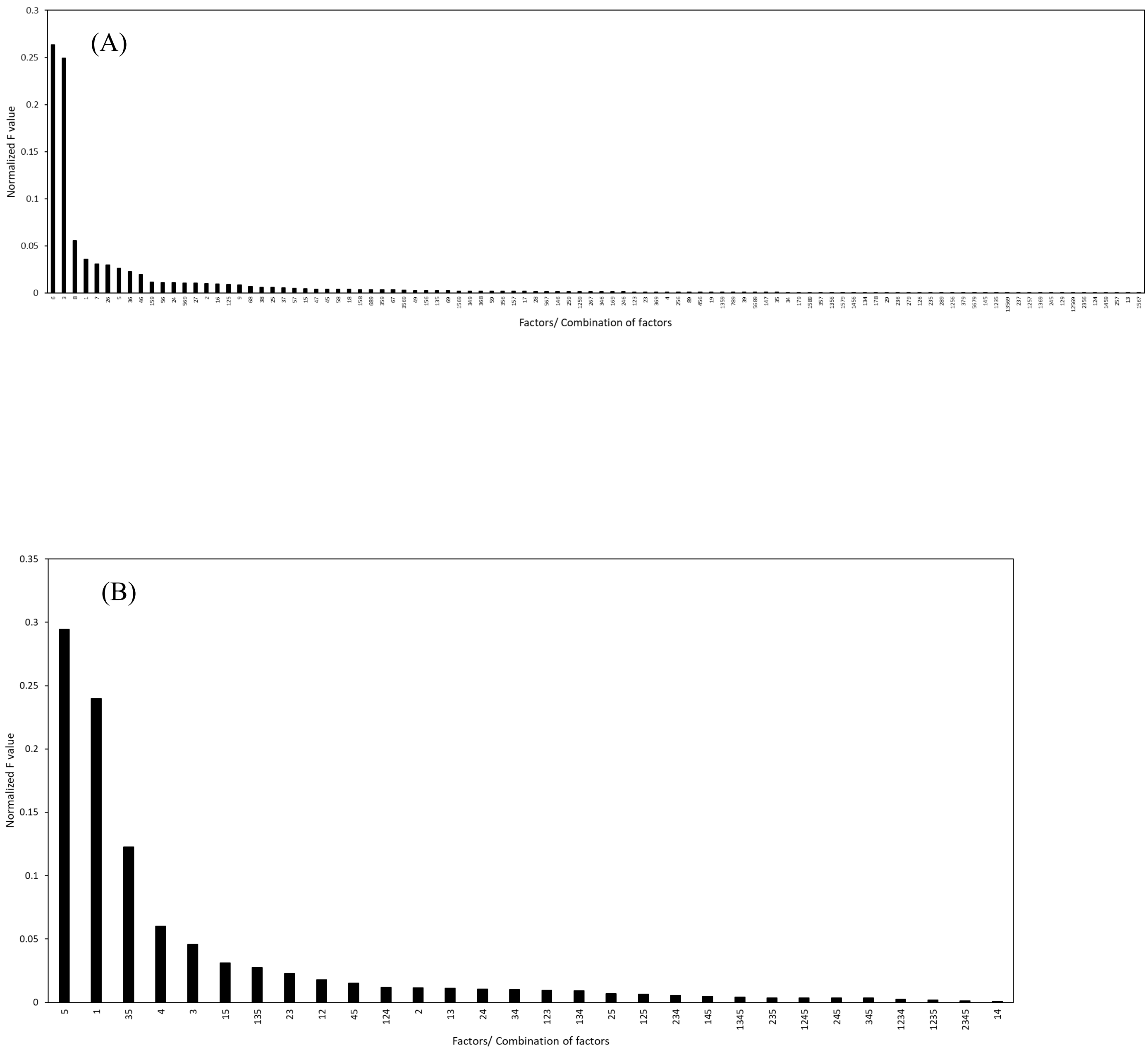


Figure S7. Effect of combined factors on $CO_2$ sequestration capacity with 100% data used for modelling. (A) direct carbonation (B) indirect carbonation.

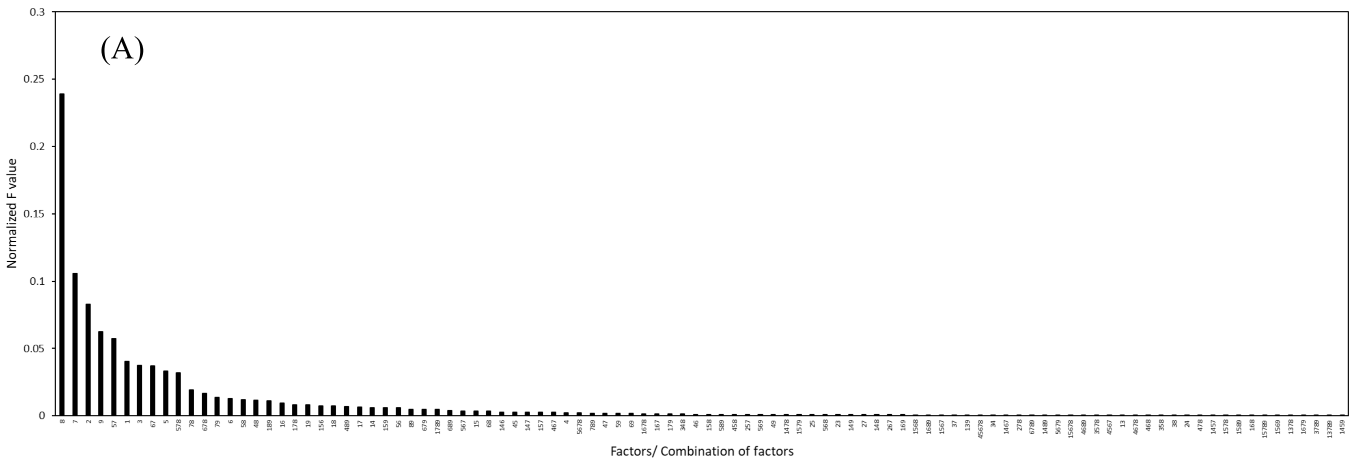


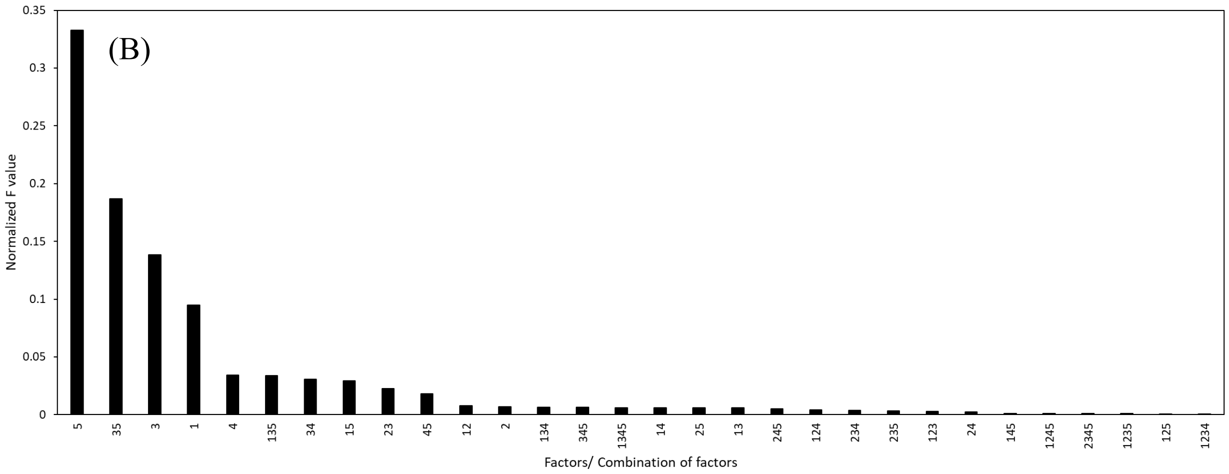


Figure S8. Effect of combined factors on $CO_2$ sequestration capacity with 80% data used for modelling. (A) direct carbonation (B) indirect carbonation.
